# Comparative studies on similarities and differences of cyclodipeptide oxidases for installation of C–C double bonds at the diketopiperazine ring

**DOI:** 10.1007/s00253-020-10392-7

**Published:** 2020-01-27

**Authors:** Lena Mikulski, Johanna Schäfer, Kirsten Brockmeyer, Rixa Kraut, Shu-Ming Li

**Affiliations:** Institut für Pharmazeutische Biologie und Biotechnologie, Philipps-Universität Marburg, Robert-Koch-Straße 4, 35037 Marburg, Germany

**Keywords:** Cyclodipeptides and derivatives, Cyclodipeptide oxidase, Diketopiperazines, Dehydrogenation

## Abstract

**Abstract:**

Cyclodipeptide oxidases (CDOs) perform dehydrogenations on diketopiperazines and play an important role in the cyclodipeptide diversification. In this study, we investigated the two known CDOs AlbA/B and Ndas_1146/7 and one new member, CDO-Np. LC-MS monitoring of 32 cyclodipeptide biotransformations in *E. coli* revealed good consumption of cyclodipeptides containing aromatic amino acids. Cyclodipeptides consisting solely of aliphatic amino acids were poor substrates. In vitro assays of 34 substrates with crude enzyme extracts and product identification proved that the CDO-Np-containing extract catalyzes the formation of two C–C double bonds in many cases. The extracts containing the two other enzymes had lower activities and catalyzed mainly didehydrogenations. For didehydrogenation, the phenylalanyl or tyrosyl site was usually preferred. No or very low acceptance of benzodiazepinediones and a 2,6-diketopiperazine proved the importance of the 2,5-diketopiperazine ring. N-Methylation at the diketopiperazine ring or prenylation of the tryptophan-containing cyclodipeptides influences the enzyme activity and product spectrum.

**Key points:**

*• Comparison of catalytic activities of three enzymes; Diverse cyclodipeptides and derivatives as substrates; Determination of double bond formation using*^*2*^*H-labeled substrates; Product identification also by interpretation of MS*^*2*^*fragmentation pattern.*

**Electronic supplementary material:**

The online version of this article (10.1007/s00253-020-10392-7) contains supplementary material, which is available to authorized users.

## Introduction

Cyclodipeptides (CDPs) containing a 2,5-diketopiperazine (DKP) backbone represent an important group of precursors in drug development (Borthwick 2012). They are mainly produced by microorganisms in various environments and are known to exhibit diverse biological and pharmaceutical activities (Borthwick 2012; Huang et al. 2010). Assembly of the DKP backbone through condensation of two amino acids can be catalyzed by non-ribosomal peptide synthetases (NRPSs) or cyclodipeptide synthases (CDPSs) (Borgman et al. 2019; Canu et al. 2020). These cyclodipeptides (CDPs) can undergo several tailoring modifications such as methylation, hydroxylation, prenylation, dimerization/coupling, and dehydrogenation, which can enhance their biological and pharmacological activities (Borgman et al. 2019; Canu et al. 2020; Giessen and Marahiel 2014; Li et al. 2014; Yu et al. 2018).

**Fig. 1:**
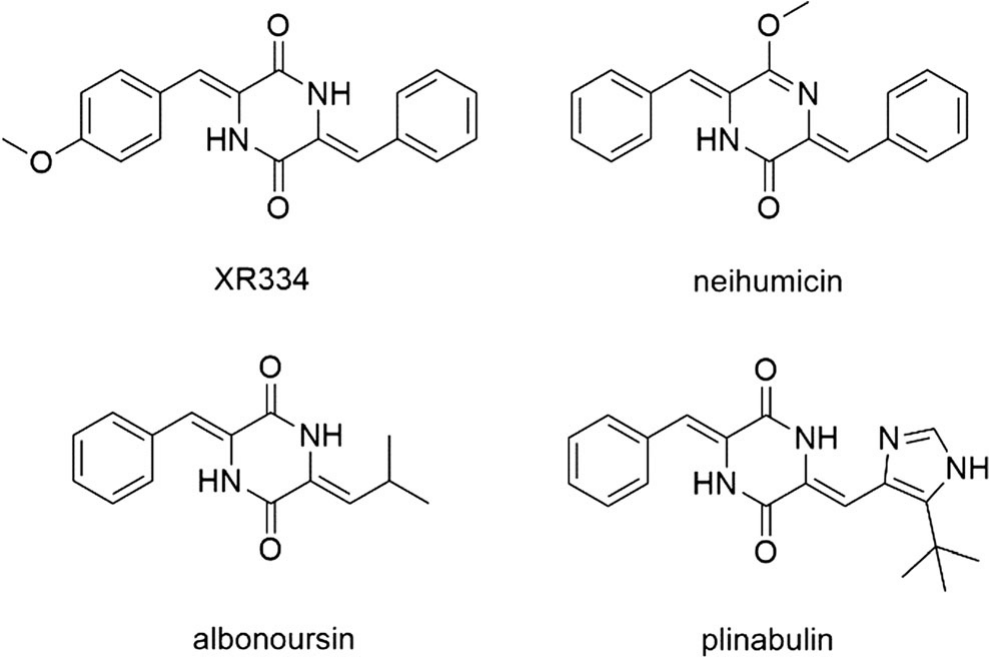
Examples of dehydrogenated cyclodipeptides

Several examples for dehydrogenated pharmacologically active compounds are listed in Fig. 1. The phenylalanyl-containing dehydrogenated CDPs XR334 and neihumicin are reported to exhibit cytotoxic and antibacterial properties (Fu et al. 2011; Giessen et al. 2013b; Zhang et al. 2013). The *cyclo*-∆Phe-∆Leu albonoursin showed inhibitory activity toward cell division (Kanzaki et al. 1999). The semi-synthetic *cyclo*-∆Phe-∆His derivative plinabulin acts as a potent microtubule inhibitor and reached phase III in clinical trials (Kanzaki et al. 2002; Yamazaki et al. 2012).

Dehydrogenation of CDPs, assembled by CDPS, and installation of C–C double bonds *exo* the DKP rings are often catalyzed by cyclodipeptide oxidases (CDOs). This was observed first for *cyclo*-l-Phe-l-Leu in *Streptomyces* sp. KO-2388 (Kanzaki et al. 1999) and 2 years later in *Streptomyces noursei* (Gondry et al. 2001). The responsible enzyme consists of two subunits, AlbA and AlbB, which are organized in the genome with an overlapping sequence of 56 bps and are located in the neighborhood of the CDPS gene *albC* (Lautru et al. 2002). The second known CDO comprising the two subunits Ndas_1146 and Ndas_1147, also located together with a CDPS gene *ndas_1148*, was identified in *Nocardiopsis dassonvillei* and was proven to be responsible for α,β-dehydrogenation in the biosynthesis of the nocazine family (Giessen et al. 2013b). CDOs are multimeric flavoenzymes with molecular weights of more than 2000 kDa and an estimated ratio between subunit B and A of 1:10 (Gondry et al. 2001; Lautru et al. 2002). Expression of subunit A without B did not result in dehydrogenation of cyclodipeptides, indicating that both subunits are necessary for enzyme activity (Belin et al. 2012). Enzyme assays under anaerobic conditions did not lead to dehydrogenated products; thus, the reaction is oxygen-dependent and the formation of H_2_O_2_ was also reported (Giessen et al. 2013b; Gondry et al. 2001; Lautru et al. 2002). Activity testing of CDOs from *Streptomyces albulus*, *S. noursei*, and *N. dassonvillei* revealed good acceptance of aromatic amino acid–containing substrates, while CDPs consisting of two aliphatic amino acids were poor substrates (Giessen et al. 2013a; Gondry et al. 2001; Kanzaki et al. 2000b; Kanzaki et al. 2004). Dehydrogenation of isoprenylated *cyclo-*Phe-His by a cell free extract of *S. albulus* showed that CDOs have the potential of accepting more complex structures than simple CDPs (Kanzaki et al. 2004). However, other modified CDPs or d-configured CDPs were not yet tested. Therefore, a systematic investigation and comparison of different CDOs toward various CDPs and derivatives will provide new insights into the catalytic features of this enzyme group, e.g., substrate and product spectra.

During our investigation on CDP-forming enzymes, we identified a CDPS from *Nocardiopsis prasina*, CDPS-Np, being responsible for the formation of tyrosine-containing DKPs with *cyclo-*l-Phe-l-Tyr as the main product (Brockmeyer and Li 2017). Two overlapping open reading frames coding for the two putative CDO (termed CDO-Np) subunits are located in direct proximity to the CDPS gene. To learn more about the catalytic properties of CDOs, we overproduced CDO-Np, together with the two known CDOs, AlbA/B and Ndas_1146/7, in *Escherichia coli* (Table S2, Supplementary Information) and used them for the biotransformation of 32 CDPs (**1a**–**3a**, **5a**–**21a**, **24a**–**25a**, and **27a**–**36a**, Fig. S1, Table S2). Based on the results of feeding experiments, 34 CDPs and derivatives (**1a**–**23a**, **25a**–**26a**, **30a**, **36a**–**37a**, **39a**–**44a**, Figs. 3, S1, and S4–S7) were chosen for enzymatic conversion with crude protein extracts. The di- and tetradehydrogenated products were identified by UV, NMR, and MS analyses.

## Materials and methods

### Chemicals

*Cyclo-*l-Phe-l-Tyr-*d*_*2*_ (**37a**) and *cyclo-*l-Trp-l-Tyr-*d*_*2*_ (**38a**) were synthesized from Boc-l-Phe and Boc-l-Trp with l-[3,3-d_2_]-Tyr methyl ester as described previously (Jeedigunta et al. 2000; Nie et al. 2012). Compounds **42a**−**44a** were synthesized as described in the literature (Dawidowski and Turlo 2014; Zeng et al. 2005). Preparation of prenylated CDPs using prenyltransferases has been reported before (Fan and Li 2013; Wunsch et al. 2015; Yin et al. 2013; Yin et al. 2007). All other cyclodipeptides used in this study were obtained from Bachem (Bubendorf, Switzerland) or synthesized as reported previously (Wollinsky et al. 2012; Yu et al. 2013). Stock solutions of 20 mM cyclodipeptides were prepared in DMSO.

### Strains and culture conditions

*N. prasina* NRRL B-16235 was kindly provided by the Agricultural Research Service Culture Collection (NRRL, Peoria, IL, USA). *S. noursei* ATCC 11455 was purchased from the American Type Culture Collection (ATCC, Manassas, VA, USA). They were cultivated according to a previously published protocol (Brockmeyer and Li 2017).

For plasmid construction, *E. coli* XL1-Blue MRF′ (GE, Amsterdam, Netherlands) was used. *E. coli* M15 [pREP4], SG 13009 [pREP4] (Quiagen, Heidelberg, Germany), and BL21 (DE3) pLysS (Invitrogen, Karlsruhe, Germany) were used for protein overproduction. Lysogeny broth (LB) medium (10 g L^−1^ tryptone, 5 g L^−1^ yeast extract, and 10 g L^−1^ sodium chloride) or Terrific Broth (TB) medium (12 g L^−1^ tryptone, 24 g L^−1^ yeast extract, 4.5 g L^−1^ glycerol, 17 mM KH_2_PO_4_, and 72 mM K_2_HPO_4_) supplemented with 50 μg mL^−1^ carbenicillin, 25 μg mL^−1^ kanamycin, or 12.5 μg mL^−1^ tetracycline was used for cultivation of recombinant *E. coli* strains.

### DNA isolation

DNA isolation and manipulation in *E. coli* were carried out as described previously (Sambrook and Russell 2001). For genomic DNA isolation from *N. prasina* and *S. noursei*, cells of 7-day-old cultures were collected and washed with TSE buffer (25 mM Tris-HCl, 10% sucrose, 25 mM ethylenediaminetetraacetic acid, pH 8.0). Isolation of genomic DNA was carried out as described previously (Brockmeyer and Li 2017).

### Construction of the expression plasmids

The nucleotide sequences encoding AlbA/B (accession number AAN0790 for AlbA and AAN07908 for AlbB) and CDO-Np (WP_017544373 for CDO-NpA and WP_026129216 for CDO-NpB) were amplified by using the primers listed in Table S1. The amplified DNA fragments encoding AlbA/B and CDO-Np were cloned into the pGEM-T Easy vector (Promega, Mannheim, Germany), resulting in plasmids pKB78 (CDO-Np) and pKB89 (AlbA/B), which were verified by sequencing (SEQLAB, Göttingen, Germany). The inserts were released afterwards by using the restriction enzymes *Nco*I and *Bam*HI, and cloned at the *Nco*I and *Bam*HI sites of pQE-60 (Qiagen, Heidelberg, Germany), resulting in pKB81 (CDO-Np) and pKB90 (AlbA/B), respectively. The plasmid Ndas_1146/7 containing the coding sequence of the CDO from *N. dassonvillei* DSM 43111 was kindly provided by Prof. Mohamed Marahiel.

### Biotransformation

Biotransformation was carried out as single experiments in Lysogeny broth (LB) medium. The expression of the respective CDOs was carried out in *E. coli* M15 cells and was induced with 1 mM IPTG for 2 h prior to feeding 0.1 mM of the respective CDPs to the cultures. After incubation for further 16 h, the cultures were extracted twice with the same volume of ethyl acetate. The ethyl acetate extracts were evaporated to dryness, dissolved in methanol, and subjected to LC-MS analysis.

### Preparation of crude protein extracts for enzyme assays

The recombinant CDOs were overproduced in *E. coli* harboring the expression plasmids. *E. coli* SG 13009 cells were used for the expression of CDO-Np and AlbA/B, and *E. coli* BL21 for Ndas_1146/7. The cells were cultivated in Terrific Broth (TB) medium with the respective antibiotics at 37 °C and 250 rpm to an absorption of 0.6 at 600 nm. Gene expression was induced by addition of IPTG to a final concentration of 0.1 mM. After cultivation for further 16 h, the cells were harvested by centrifugation at 3000 rpm for 15 min and resuspended in lysis buffer (50 mM sodium dihydrogen phosphate, 300 mM sodium chloride, 10 mM imidazole, 5 μg mL^−1^ RNAse, 10 μg mL^−1^ DNAse, and 0.5% Triton-X 100, pH 8.0) and incubated for 30 min at 4 °C. Subsequently, the cells were lysed using a Branson sonifier 250 (Thermo Fisher Scientific, Waltham, MA, USA) and the debris was separated from the cell lysate by centrifugation at 13000 rpm for 30 min. The lysate was desalted using a Sephadex G25 NAP-5 column (GE-Healthcare, Solingen, Germany) and a storage buffer (50 mM Tris-HCl pH 7.5 at 4 °C, 15% (*w*/*v*) glycerol), and subsequently stored at − 80 °C. The total protein content of the CDO-enriched crude extracts was determined by using the Bradford method (Bradford 1976).

### In vitro enzyme assays

Enzyme reactions for activity determination were carried out in 25 μl Tris-HCl buffer (50 mM, pH 7.5) with 1 mM cyclodipeptide, 1 mM phenazine methosulfate (PMS) (Sigma Aldrich, Darmstadt, Germany), and 40 μg crude enzyme extracts at 40 °C. Assays were evaporated and the residue dissolved in methanol. The assays for product isolation in 5–10 mL contained 2.5–5.0 mg crude enzyme extracts and were incubated at 60 °C for 30 min–8 h. The reaction was terminated with the equal volume of methanol for analysis or extracted three times with the equal volume of ethyl acetate for isolation. The reaction mixtures were centrifuged at 13000 rpm and then subjected to LC-MS analysis. Enzyme assays were performed in duplicate for time curves and incubation with dehydrogenated substrates. The ethyl acetate phases were evaporated and the extracts were subjected to isolation on HPLC.

### HPLC analysis and product isolation

Cyclodipeptides and derivatives were analyzed on an Agilent 1200 HPLC system equipped with an Eclipse XDB-C18 (5 μm, 4.6 × 150 mm) column (Agilent Technologies, Santa Clara, USA). A flow rate of 1 mL min^−1^ with solvents A (water) and B (acetonitrile) was used as mobile phase with a linear gradient of 20 to 60% B over 15 min. For isolation of didehydrogenated cyclodipeptides, an Eclipse XDB-C18 (5 μm, 9.4 × 250 mm) column (Agilent Technologies) was used with a flow rate of 4.5 mL min^−1^. Didehydrogenated CDO products were isolated isocratically with 25% B for **1b** and **1c**, **2b** and **2c**, 30% B for **3b**, and 45% of B for **5b**. Tetradehydrogenated products were isolated using gradients of 30 to 60% B in 20 min for **1d**, 20 to 60% B in 20 min for **2d**, 40 to 60% B in 25 min for **3d**, and 50 to 70% B in 25 min for **5d**. 1.6 mg of **1b** and 0.9 mg of **1c** were isolated as white powders from a 10 mL reaction mixture of **1a** with CDO-Np. 1.1 mg and 0.8 mg of **2b** were obtained as white powder from 10 mL reaction mixtures of **2a** with AlbA/B and 10 mL with CDO-Np. The reaction mixture of **2a** with CDO-Np also delivered 1 mg of **2c** as a white powder. 1.9 mg and 2.9 mg of **3b** as a brownish powder were isolated from 10 mL reaction mixture of **3a** with AlbA/B and 10 mL with CDO-Np. Purification of the reaction mixtures of **5a** with CDO-Np and AlbA/B yielded in 1.2 and 2.8 mg of **5b** as brownish powder, respectively. 0.9, 1.2, 1.4, and 2.3 mg of **1d**, **2d**, **3d**, and **5d** were isolated as yellow powders from 5 mL reaction mixtures of CDO-Np assays with **1a**, **2a**, **3a**, and **5a**, respectively. **3d** and **5d** were not soluble in water or ethyl acetate and thus the assays were centrifuged for 15 min at 5000 rpm and the supernatant was discarded. Subsequently, the pellet was solved in a mixture of DMSO and methanol (2:1) and centrifuged for 10 min at 13000 rpm prior to HPLC isolation.

### LC-MS analysis

LC-MS analysis was performed on an Agilent 1260 series HPLC system equipped with a Multospher 120 RP-18 column (250 × 2 mm, 5 μm, CS-Chromatographie Service, Langerwehe, Germany). Solvents A (water) and B (acetonitrile), each substituted with 0.1% formic acid, were used as mobile phases with a flow rate of 0.5 mL min^−1^ with a linear gradient of 5 to 100% B in 10 min for analysis of the in vitro assays. For analysis of the samples of the biotransformation experiments, a flow rate of 0.25 mL min^−1^ with a linear gradient of 5 to 100% B in 40 min was used. Positive ion mode electrospray ionization (ESI) in a micrOTOF-Q III mass spectrometer by Bruker Daltonics (Bremen, Germany) was used for mass detection. The parameters were set as follows: capillary voltage of 4.5 kV and a collision energy of 8.0 eV. LC-MS data were processed using Bruker Compass Data Analysis version 4.2 (Build 383.1) software.

### NMR spectroscopy

For structure elucidation, the isolated compounds were dissolved in DMSO-d_6_ and ^1^H-spectra were taken on an ECA500 spectrometer (JEOL, Tokyo, Japan). The spectra were processed with MestReNova version 6.0.2.2-5475. Chemical shifts are referenced to that of the solvent signal.

## Results

### Sequence analysis of CDO-Np and comparison with known CDOs

WP_017544373 for CDO-NpA and WP_026129216 for CDO-NpB from *N. prasina* comprise 185 and 105 amino acids, respectively. Their coding sequences overlap with each other by 20 bps. Sequence comparison revealed that CDO-NpA shares a sequence identity of 41% with AlbA and 68% with Ndas_1146 on the amino acid level. The sequence identities of the small subunit CDO-NpB with AlbB and Ndas_1147 were found to be 48 and 66%, respectively. It can therefore be expected that the holoenzyme CDO-Np also catalyzes the dehydrogenation of CDPs. The coding sequences of CDO-NpA and CDO-NpB were amplified together from the genomic DNA of *N. prasina* and cloned into pQE-60, resulting in pKB81. Feeding of *cyclo*-l-Phe-l-Tyr into *E. coli* cells carrying the expression construct pKB81 and LC-MS monitoring of the biotransformation revealed clear formation of di- and tetradehydrogenated products (Fig. S2, Table S3).

### Biotransformation of 32 substrates revealed a broad substrate specificity of CDOs toward CDPs containing aromatic amino acids

After successful confirmation of the CDO-Np function, AlbA/B and Ndas_1146/7 were also overproduced in *E. coli* strains by induction with IPTG at a final concentration of 1.0 mM for 2 h. A total of 32 CDPs with final concentrations of 0.1 mM were then fed to the *E. coli* cells. After cultivation for further 16 h, the cultures were extracted with ethyl acetate. Monitoring of successful biotransformation was carried out on HPLC equipped with a high-resolution mass spectrometer. The [M+H]^+^ ions of the substrates, and the di- and tetradehydrogenated products were used for detection and quantification. In general, aromatic amino acid–containing CDPs were much better converted than those consisting exclusively of aliphatic amino acids. As given in Table S2, several CDPs containing at least one aromatic amino acid, e.g., *cyclo*-l-Phe-l-Leu (**1a**), *cyclo*-l-Phe-l-Tyr (**2a**), *cyclo*-l-Tyr-l-Trp (**3a**), *cyclo*-l-Phe-l-Trp (**5a**), *cyclo*-l-Phe-l-His (**6a**), *cyclo*-l-Trp-l-Leu (**7a**), *cyclo*-l-Trp-l-His (**13a**), *cyclo*-l-His-l-Ala (**24a**), and *cyclo*-l-Tyr-l-Tyr (**25a**) were well accepted by the three CDOs with total product yields of 50–97%, except for conversion of **3a** by Ndas_1146/7. However, CDPs with tryptophan and one aliphatic amino acid appeared to be poor substrates for the three CDOs. Anthranilic acid–containing CDPs with a benzodiazepine dione skeleton (**35a** and **36a**) were not consumed, indicating the importance of the DKP ring for an acceptance by CDOs. There are CDPs, which were well accepted by two enzymes, e.g., *cyclo*-l-Tyr-d-Trp (**8a**) and *cyclo*-l-Tyr-Gly (**27a**) with product yields of more than 60% by CDO-Np and AlbA/B, but only 4.9 and 12.6% by Ndas_1146/7. It was also observed that some CDPs were well converted by only one enzyme, e.g., *cyclo*-l-Tyr-l-Ser (**15a**) by CDO-Np.

**Fig. 2:**
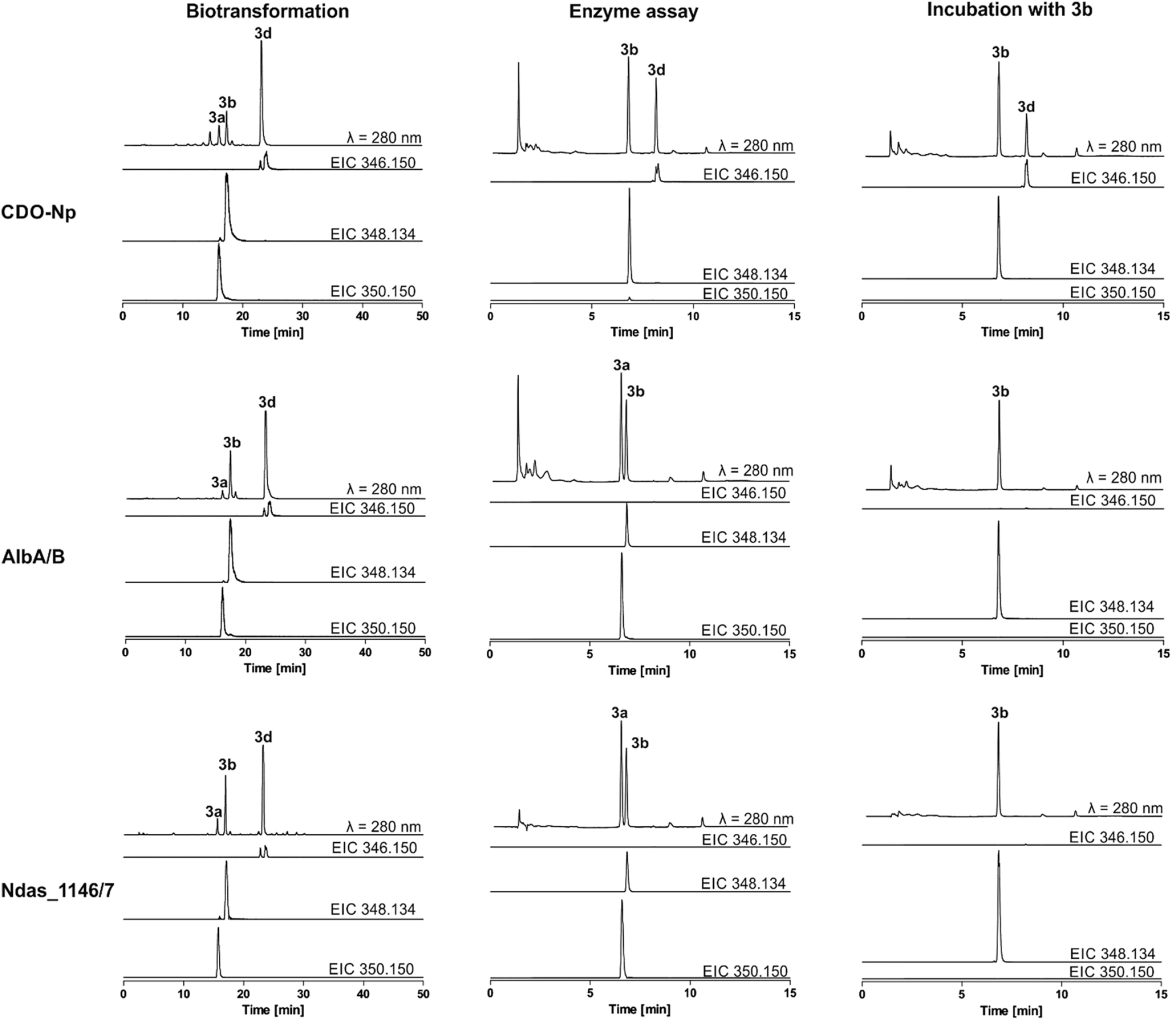
Conversion of *cyclo*-l-Trp-l-Tyr (**3a**) in vivo and in vitro as well as in vitro assays of **3b** by the three CDO-containing extracts. For biotransformation, the fed cultures were maintained at 37 °C for further 16 h. Enzyme assays were carried out at 40 °C for 2 h. Top chromatograms show the absorption at 280 nm and the three below the extracted ion chromatograms of the substrates and respective products with a tolerance of ± 0.005

Taking the number of the installed C–C double bonds into consideration, **1a**, **2a**, **6a**, **24a**, and **25a** were mainly converted to tetradehydrogenated products by the bacteria expressing the three enzymes. Didehydrogenated compounds were main products of **5a** and **7a** by all three enzymes. Differences were found with **3a**, leading to didehydrogenated derivatives by CDO-Np and AlbA/B, and tetradehydrogenated derivatives by Ndas_1146/7 as main products. In the case of **13a**, CDO-Np converted it mainly to di-, and the other two enzymes to tetradehydrogenated products. In Fig. 2, the biotransformation of **3a** is illustrated as an example, while all product yields obtained by the biotransformation experiments are given in Table S2. The structures of the products are described below. The expression of the respective CDO genes, as well as the intake of the substrate into the cell, cannot be accurately monitored in the biotransformation experiments. Therefore, no product formation does not necessarily mean no acceptance of a substrate by a given enzyme. In vitro enzyme assays could verify the findings and provide more insights into the catalytic activities of the enzymes.

### In vitro enzyme assays confirmed similarities and differences in substrate specificity

**Fig. 3:**
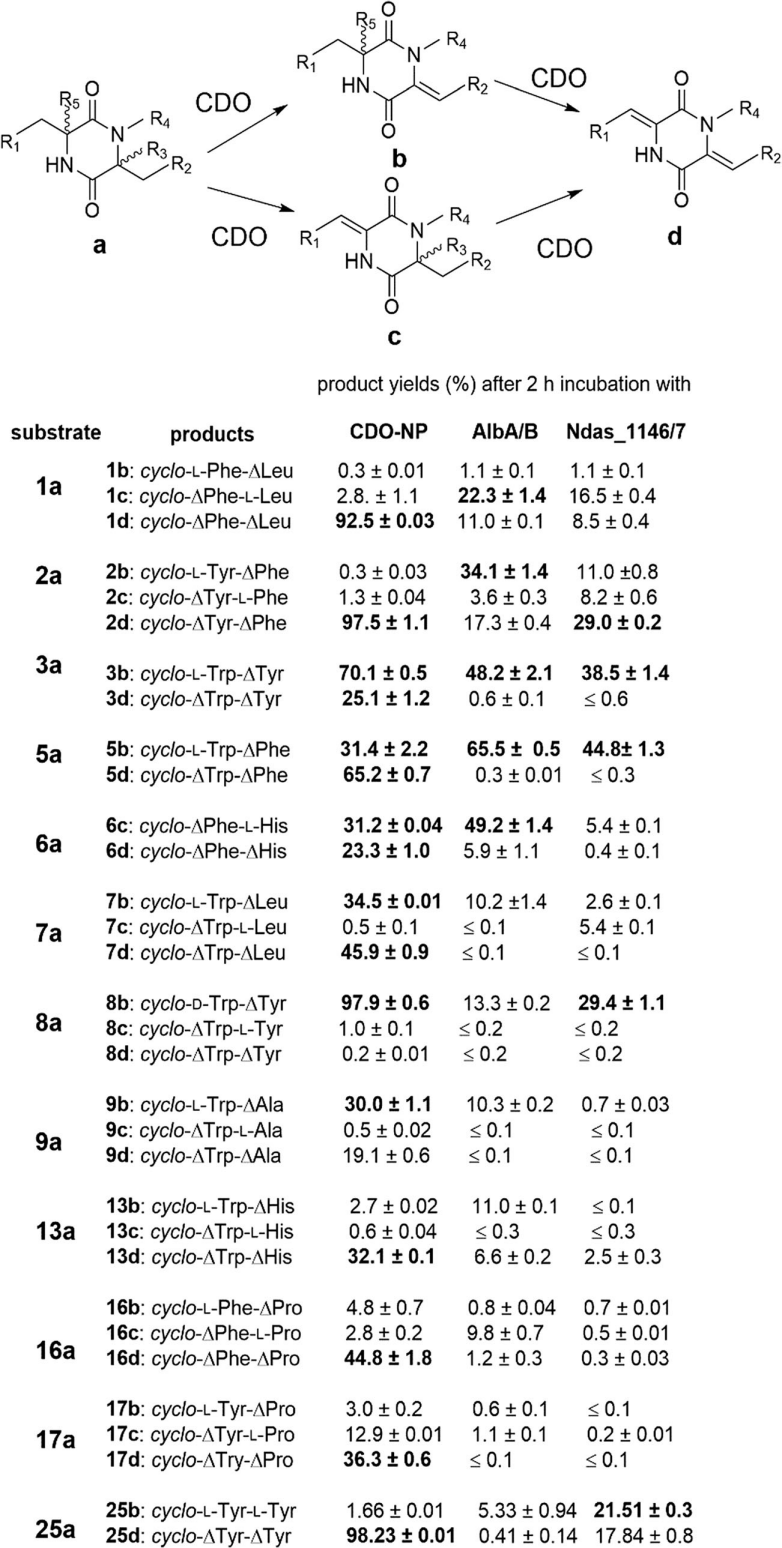
Dehydrogenation reactions of CDPs catalyzed by the three CDOs with overall product yields of more than 30% are shown. Product yields of more than 20% are highlighted in bold. Product yields were calculated by using the area under the curves of the respective extracted ion chromatograms and ± indicates the mean value of two independent experiments. See Fig. S1 for detailed structures

Preliminary experiments showed that high conversion yields of various CDPs can be achieved with crude enzyme extracts from *E. coli* strains containing the three overproduced CDOs, while incubation with heat-inactivated extracts and extracts from cells with the empty vector did not lead to the formation of dehydrogenated products (data not shown). Attempts to purify CDO-Np using nickel affinity chromatography by cloning the overlapping sequence as in the genome and placing the coding sequence for 6xHis to the N-terminus of CDO-NpA resulted in an active protein, but only with 2.9% relative activity of the original crude extract (Fig. S3). A possible reason could be a disruption of the multimeric protein complex during the purification step. To compare the dehydrogenation reactions of the CDOs, we therefore used the desalted crude enzyme extracts for in vitro assays with 34 selected CDPs (**1a** – **23a**, **25a** – **26a**, **30a**, **35a** – **36a**, and **39a** – **44a**, Fig. 3 and Figs. S4–S7). For a better comparison, the cultures were treated in similar ways and the enzyme assays containing 40 μg crude enzyme extracts were incubated at 40 °C for 2 h. Unfortunately, the concentration of the overproduced CDOs in the crude protein extracts could not be estimated. SDS-PAGE analysis showed no significant differences in protein band pattern between the overexpression strains and the control culture with the empty vector (data not shown). In total, 93 enzyme assays were carried out and analyzed on LC-MS. The chromatograms with UV absorption and extracted ion chromatograms for the respective substrates, and di- and tetradehydrogenated products are given exemplarily for **3a** in Fig. 2. Chromatograms for other CDPs (**1a**, **2a**, and **4a**–**23a**, **25a**–**26a**, **30a**, **35a**–**36a** and, **39a**–**44a**) are provided as Figs. S8–S41.

Detailed inspection of the LC-MS results revealed that one didehydrogenated product each was detected in 56 assays and two didehydrogenated products in 33 reactions, while one tetradehydrogenated derivative each was detected in 48 assays. For a given substrate, HPLC chromatograms showed the same retention time for the respective di- and tetradehydrogenated products of the three CDO assays, indicating the presence of the same products. The structures of these products were elucidated (see below) and assigned to different peaks (Figs. 2 and 3, and Figs. S8–S41). For simplicity, we termed the products **b**, **c**, and **d**. In product **b**, the double bond is located at the side of the smaller and in **c** the bigger residue. Product **d** bears two double bonds (see below for their structures and identification). Reactions with more than 30% conversion are summarized in Fig. 3, which in most cases correspond well to those obtained from the feeding experiments given in Table S2. However, there are also differences for a small number of CDPs. Most of the 34 CDPs and derivatives were accepted by CDO-Np with total product yields of 0.2 to 99.5%. **1a** – **3a** and **5a** were very well consumed by the other two enzymes. Interestingly, **8a** was well accepted in vitro by Ndas_1146/7, with a product yield of 29.4%, although it was a very poor substrate in the biotransformation experiments (Table S2). In comparison, the conversion of **6a** by Ndas_1146/7 was higher in the biotransformation experiment than in the enzyme assay. **18a** was better accepted by the CDO-Np-containing extract than in the biotransformation experiment and vice versa results were observed for **18a** with Ndas_1146/7. All these results could be explained by different availability of the substrates of in vitro and in vivo assays. It can, however, not be excluded that they are also caused by different levels of gene expression.

In addition, CDPs containing d-configured amino acids (**8a**–**12a** and **19a**–**21a**), prenyl indole moieties (**4a**, **22a**, **23a**, **26a**, and **41a**), or modifications at the diketopiperazine ring (**36a**–**37a** and **42a**–**44a**, Fig. S1) were also tested with the three enzymes. The results showed that the conversion yields of d-amino acid–containing CDPs were much lower than those consisting merely of l-configured amino acids (Fig. 3 and Fig. S5). The majority of the prenylated CDPs were converted, but in distinctly lower amounts compared with the unprenylated congeners (Fig. S6). Compound **42a**, with a 2,6-diketopiperazine core, was not consumed by the three CDOs. Modifications on the diketopiperazine ring influenced the CDO reaction tremendously. Only the methylated *cyclo*-l-Phe-l-Phe derivative, **43a** showed a good conversion by CDO-Np with one didehydrogenated product. In comparison, the non-methylated *cyclo*-l-Phe-l-Phe (**30a**) was converted by this enzyme to a tetradehydrogenated derivative (Figs. S4, S7, S33, S40, and S41).

### Structure elucidation of the dehydrogenated products

To confirm the structures, especially to determine the position of the C–C double bond in the didehydrogenated derivatives, products were isolated on HPLC from enzyme assays with selected substrates **1a**–**3a** and **5a** under different conditions. These include **1b**–**1d** from assays of **1a** with CDO-Np. **2b**, **3b**, and **5b** were isolated from enzyme assays of the respective substrates, and **2a**, **3a**, and **5a**, with both AlbA/B and CDO-Np. **2c** and **2d** were isolated from the reaction mixture of **2a** with CDO-Np, and **3d** and **5d** from those of **3a** and **5a** with CDO-Np (see “Materials and methods” for details). The isolated products were subjected to NMR and MS analyses. This confirmed the presence of di- and tetradehydrogenated derivatives by detection of [M+H]^+^ ions with two or four Daltons less than those of the respective substrates and the presence of one or two additional signals for olefinic protons in the range of 5.26–7.12 ppm (Tables S4–6 and Figs. S42–51). Correspondingly, one of the two three-spin systems related to the diketopiperazine ring disappeared in the spectra of didehydrogenated products. In the case of tetradehydrogenated products, the two three-spin systems were replaced by signals of two olefinic protons, confirming unequivocally the structures of **1d**, **2d**, **3d**, and **5d**. The NMR data of **1d** were furthermore in good agreement with those reported previously for *cyclo-*ΔLeu-ΔPhe (Kanzaki et al. 2000c). **1b** and **1c** can be easily identified as *cyclo*-ΔLeu-l-Phe and *cyclo*-l-Leu-ΔPhe by the different chemical shifts of the olefinic protons, 5.26 ppm with a low and 6.66 ppm with a highly conjugated system, and by comparison with the published data (Kanzaki et al. 2000c).

**Fig. 4:**
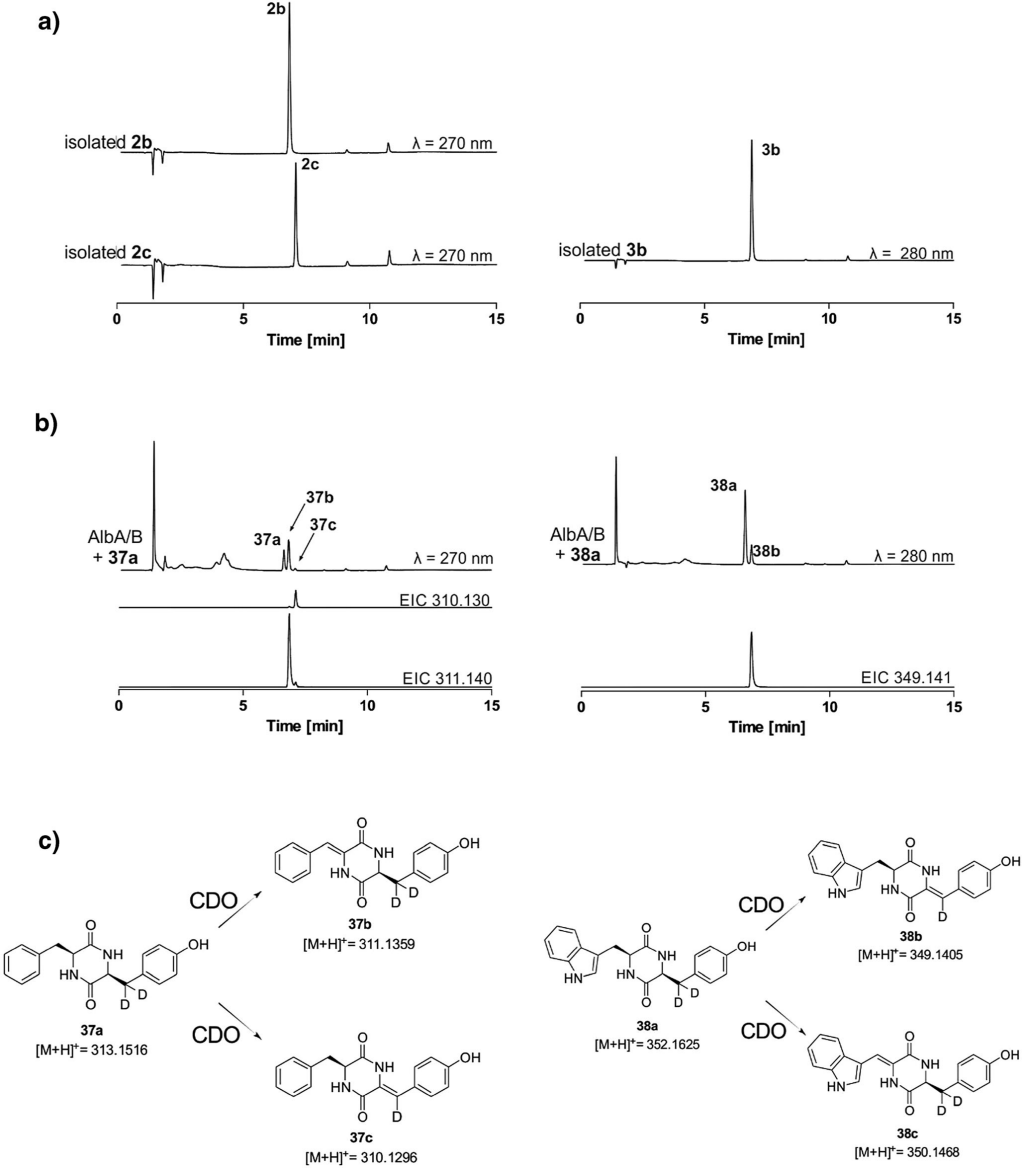
Determination of C–C bond positions in **2b**, **2c**, and **3b**. LC-MS analysis of authentic standards (A) and of incubation mixtures of deuterated CDPs **37a** and **38a** with AlbA/B (B) as well as reaction schemes with expected [M+H]^+^ ions (C). EICs with a tolerance range of ± 0.005 refer products with one additional double bond

The two samples each of **2b**, **3b**, and **5b**, which were isolated from two enzyme assays with AlbA/B and CDO-Np, respectively, have almost identical NMR spectra, proving the presence of the same products of different CDO reactions. To distinguish the C–C double bond positions in **2b**, **2c**, and **3b**, we chemically synthesized *cyclo-*l-Phe-l-Tyr-*d*_*2*_ (**37a**) and *cyclo-*l-Trp-l-Tyr-*d*_*2*_ (**38a**), and used them as substrates for assays with the three CDOs. The reaction mixtures were analyzed on LC-MS afterwards by using the isolated **2b**, **2c**, and **3b** as authentic standards. All three enzymes showed similar behaviors toward **37a** and **38a** as for **2a** and **3a** regarding acceptance and product spectra. The results with AlbA/B are illustrated exemplarily in Fig. 4. Detection of a [M+H]^+^ ion at m/z 311.1359 for **37b** due to the loss of two hydrogens and m/z 310.1296 for **37c** based on the elimination of one hydrogen and one deuterium proved unequivocally the positions of the C–C double bonds. In the incubation mixture of **38a**, the only product was **38b** with a [M+H]^+^ ion at m/z 349.1405. This was three Daltons less than that of the substrate and thus proved *cyclo*-l-Trp-ΔTyr as the enzyme product. In this way, **2b**, **2c**, and **3b** were identified as *cyclo*-l-ΔPhe-l-Tyr, *cyclo*-l-Phe-ΔTyr, and *cyclo*-l-Trp-ΔTyr, respectively.

Comparison of the signals of **3b** and **5b** in the ^1^H NMR spectra revealed very similar chemical shifts and coupling patterns, especially for signals of the tryptophanyl moiety, i.e., H-6, H-7, and the protons at the indole ring. All these indicate the presence of a double bond at the phenylalanyl moiety in **5b**. The assumption that **5b** is *cyclo*-l-Trp-ΔPhe is strongly supported by MS^2^ fragmentation patterns obtained by ESI-CID-MS/MS (Table S7, Figs. S52–S56, and Fig. S58).

**Fig. 5:**
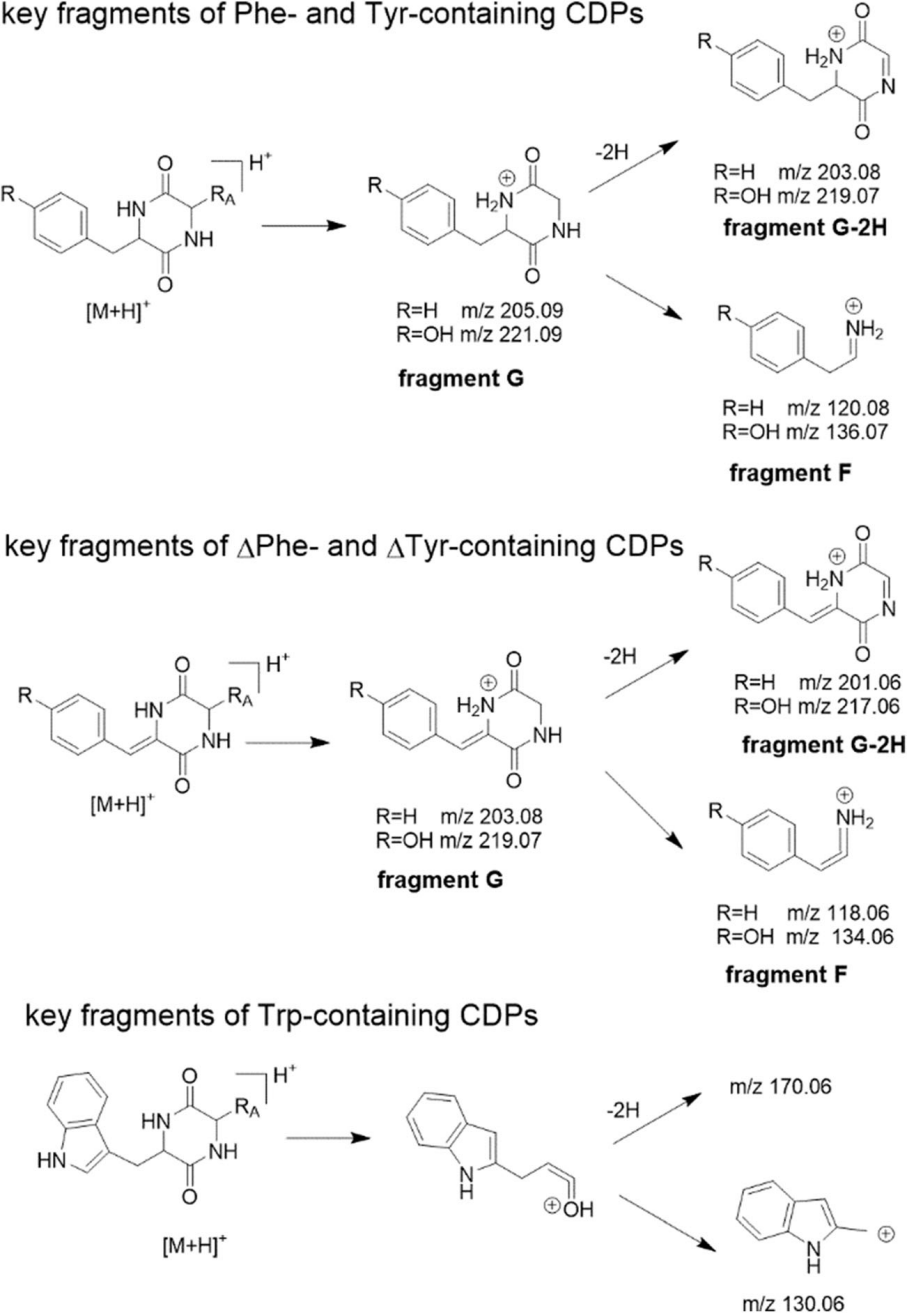
Key fragmentation of phenylalanine-, tyrosine-, and tryptophan-containing CDPs

Specific MS^2^ fragmentation patterns of cyclodipeptides have been reported (Furtado et al. 2007; Guo et al. 2009). In order to find the key MS fragments of dehydrogenated CDPs, we used incubation mixtures of the three CDOs with **1a**–**3a** for MS^2^ analysis (Figs. S52–56). In a previous study, fragment F (Fig. 5) was observed as major peaks in the MS^2^ spectra of phenylalanine- and tyrosine-containing CDPs (Guo et al. 2009), which was confirmed in this study by detection of ions at m/z 120.08 and 136.07 in **1a**, **1b**, **2a**, and **2c** (Table S7, Fig. S52 and Fig. S55). In the derivatives with ∆Phe or ∆Tyr, the corresponding fragments appeared at m/z 118.06 (**1c** and **2b**) and m/z 134.06 (**2c** and **3b**). The fragment pairs G and G-2H at m/z 205.09/203.08 for phenylalanyl- (**1a**, **1b**, **2a**, and **2c**) and at m/z 221.09/219.07 (**2a**, **2b**, and **3a**) for tyrosyl-containing CDPs are also important for the identification of the respective residues. Again, the corresponding fragments in the derivatives with ∆Phe or ∆Tyr are two Daltons smaller and were detected at m/z 203.08/201.06 in **1c** and **2b** and at m/z 219.07/217.06 in **2c** and **3b**. The key fragments for tryptophan-containing CDPs were found at m/z 170.06 and 130.06 (Table S7, Fig. 5 and Figs. S52–S56). A more detailed fragmentation scheme and summary of the fragments are provided as Fig. S86 and Table S7, respectively. The detection of ions at m/z 203.081, 201.066, and 118.065 for ∆Phe, and at m/z 170.060 and 130.064 for the tryptophanyl residue confirmed the structure of **5b** (Fig. S58) as *cyclo*-l-Trp-∆Phe. Afterward, we inspected the MS^2^ spectra of the product peaks in the incubation mixtures of all enzyme assays for the presence of ions derived from fragments F, G, and G-2H as well as those at m/z 170.06 and 130.06 (Figs. S52–S85), because all of these substrates contain at least one phenylalanyl, tyrosyl, tryptophanyl, or a dehydrogenated residue. In this way, the position of the installed C–C double bond can be easily determined and the products identified, which are presented in Tables S3 and S7 as well as Fig. 3, Figs. S8–S41, and Figs. S52–S85.

Until now, all the isolated didehydrogenated products have *Z-*configuration (Arunrattiyakorn et al. 2007; Kanzaki et al. 2000a; Kanzaki et al. 2000b; Kanzaki et al. 2000c; Kanzaki et al. 2002; Kanzaki et al. 2004). The presence of *E*-isomers are reported to be products of chemical rearrangement (Kanzaki et al. 2002), which was also observed in this study (Fig. S87). In this experiment, **2a**, **3a**, and **6a** were incubated with 40 μg CDO-Np for 2 h and the assays were quenched with the equal volume of methanol. The mixtures were subsequently incubated at 22 °C under day light for 0.5 and 8 h and analyzed on LC-MS. As shown in Fig. S87, **2d**, **3b**, **3d**, **6c**, and **6d** were detected as predominant peaks in the extracted ion chromatograms (EICs) after incubation for 30 min. Only a minor peak 6d* with the same mass as **6d** was observed. In contrast, one significant additional peak each, 2d*, 3b*, 3d*, or 6d*, was detected for the corresponding products after incubation for 8 h. In the case of **6d**, the rearrangement even reached approximately 50%.

### Number and preferred position of the installed double bonds

The three CDOs were all able to perform di- and tetradehydrogenation reactions on CDPs. Comparing the yields of the two possible didehydrogenated products showed that the C–C double bond is more easily installed at the phenylalanyl or tyrosyl moiety. This was observed for **1c**, **2b**–**3d**, **5b**, and **6c** in the assays with AlbA/B and Ndas_1146/7 as well as for **7b**–**9b**, **29c**, and **15c** in the assays with all three enzymes. Some of them are predominant products of the respective assays, such as **2b** and **6c** in assays with AlbA/B, **3b** and **5b** in those of AlbA/B and Ndas_1146/7, **8b** in those of CDO-Np and Ndas_1146/7, and **15c** with CDO-Np. Only one exception was observed for CDO-Np with **18a**, where *cyclo*-l-Phe-∆Pro was the main product. Didehydrogenated products with a ∆Trp moiety were scarcely detected in the assays, although tetradehydrogenated products with high yields were found in the assays of **3a**, **5a**, **7a**, **9a**, and **13a**, indicating that the dehydrogenation of the Trp moiety might be the second step. Methylation of the DKP ring influenced the CDOs activity. Both phenylalanyl moieties in **30a** were dehydrogenated, resulting in the tetradehydrogenated derivative as the sole product. In **43a**, the phenylalanyl moiety adjacent to the methylation at the DKP ring was not dehydrogenated, resulting in only one didehydrogenated and no tetradehydrogenated product.

Tetradehydrogenated derivatives with product yields of more than 20% were detected in ten assays with CDO-Np (**1a**–**3a**, **5a**–**7a**, **13a**, **16a**, **17a**, and **25a**), one with Ndas_1146/7 (**2a**), and none with AlbA/B. Because the concentration of the CDOs in the enriched extracts cannot be determined, the observed absence of tetradehydrogenated products could be caused by different behaviors of the enzymes or by low concentrations of AlbA/B and Ndas_1146/7 in the extracts.

**Fig. 6:**
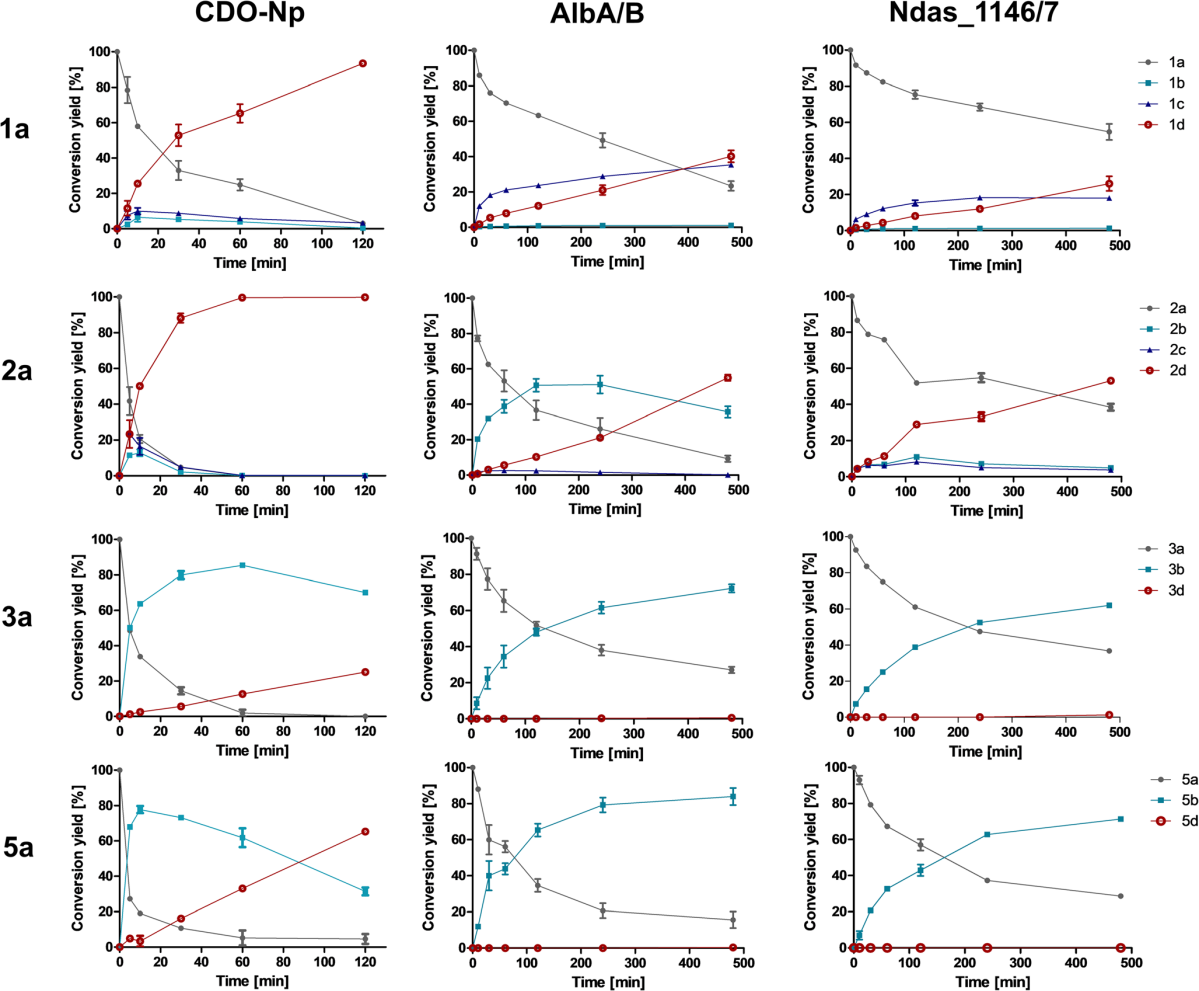
Time-dependent in vitro conversion of **1a**–**3a** and **5a** by the three CDOs. Error bars show mean values of two independent experiments

To monitor the conversion of **1a**–**3a** and **5a** to their dehydrogenated products by the CDOs over time, we carried out incubations with 40 μg crude protein extracts in 25 μl assays (Fig. 6). The data of reactions for 2 h corresponded very well to those listed in Fig. 3 and Figs. S4–S7. CDO-Np showed very high activity toward **1a** and **2a,** and produced mainly tetradehydrogenated derivatives. After 30 min, the formation of **1d** and **2d** had already reached 52 and 88%, respectively. In the reaction mixtures of **3a** and **5a**, the didehydrogenated products **3b** and **5b** achieved their maximal values of 80 and 83% after 30 min, and decreased during further incubation. In comparison, the tetradehydrogenated products increased slowly, but steadily, to 25 and 66% after 120-min incubation. AlbA/B and Ndas_1146/7 showed similar behaviors toward **1a**, **3a**, and **5a**. Formation of **1c** and **1d** was very slow in both reaction mixtures. In the first 350 min, slightly more **1c** than **1d** was produced and after that vice versa. Only one didehydrogenated product **3b** or **5b** was detected in the reaction mixtures of AlbA/B and Ndas_1146/7 with **3a** and **5a**, with comparable product yields between 73 and 84% after incubation for 8 h. The behavior of AlbA/B with **2a** was similar to CDO-Np with **3a** and **5a**, i.e., relative high conversion to **2b** occurred at the beginning and decreased after 240 min. The formation of **2d** increased steadily and exceeded that of **2b** after 8 h. The production of tetradehydrogenated products **3d** and **5d** was not achieved for AlbA/B and Ndas_1146/7 after 8 h of incubation, in contrast to CDO-Np, which produced **3d** and **5d** already after 10 min of incubation.

The inability of AlbA/B and Ndas_1146/7 to produce tetradehydrogenated derivatives **3d** and **5d** was also confirmed by incubation with the didehydrogenated products of **1a**–**3a** and **5a** with CDO-enriched extracts for 2 h (Figs. S88–S91). Incubations with **1a**–**3a** and **5a** were used as controls. Interpretation of these results revealed that yields of the tetradehydrogenated products in reaction mixtures of the three enzymes were in agreement with those of the incubations of **1a**–**3a** and **5a** in many cases. **3b** and **5b** were well accepted by CDO-Np, while they were not consumed by AlbA/B and Ndas_1146/7, confirming them as the sole products of **3a** and **5a** for these enzymes. Additionally, the two didehydrogenated derivatives of **1a** were differentially consumed by a given CDO, indicating a preferred order for installation of the two C–C bonds depending on the respective amino acid during the formation of tetradehydrogenated products. **1c** was converted less than **1b** by all three enzymes, while there was no difference for **2b** and **2c**, matching the observation that phenylalanine and tyrosine are the favored amino acids.

## Discussion

Cyclodipeptide oxidases, mainly from bacteria, catalyze di- and tetradehydrogenation of CDPs and install *exo* C–C double bonds to the DKP rings (Giessen et al. 2013b; Gondry et al. 2001; Kanzaki et al. 2000b, c, 2002). The genes coding for CDOs are usually members of *cdps*-containing clusters (Giessen et al. 2013b). In comparison with the increased interests in the function of CDPS and cytochrome P450 enzymes (Borgman et al. 2019; Canu et al. 2020), only few studies on CDOs are reported. Previous studies with limited CDPs showed a broad substrate specificity of AlbA/B (Gondry et al. 2001; Kanzaki et al. 2000b, c, 2002). The CDPS Ndas_1148 from *N. dassonvillei* produces **1a**, **2a**, and **30a** as main products, which serve as substrates for Ndas_1146/7 for dehydrogenation. The activity of the CDO Ndas_1146/7 for two successive α,ß-dehydrogenations was investigated by co-expression with Ndas_1148 (Giessen et al. 2013b).

In this study, the substrate specificity and the products of the newly discovered CDO-Np from *N. prasina* and the two known CDOs were extensively studied and compared by biotransformation of 32 CDPs and incubation of 34 selected substrates with crude protein extracts. **2a** and **7a** with a ratio of 6.8:1 were identified as main products of CDPS-Np in the cluster containing *cdo-Np* from *N. prasina* (Brockmeyer and Li 2017) and can be expected as the natural substrates of CDO-Np. As given in Table S2, both compounds were well converted to tetradehydrogenated products. In vitro incubation of **2a** with CDO-Np led to an almost complete conversion to **2d** (Fig. 3). In the previous study (Gondry et al. 2001), AlbA/B converted **1a** after incubation for 18 h mainly to **1d** and **6a** to didehydrogenated derivatives. The position of the installed C–C double bond was not given in that study. For both substrates, we detected tetradehydrogenated derivatives as main products in biotransformation (Table S2). In the in vitro assay with AlbA/B, the didehydrogenated derivative **6c** was observed as main product after incubation for 2 h and **6a** was even better accepted than **1a** (Fig. 3). When prolonging the incubation time of **1a** with AlbA/B, the yield of **1d** increased steadily and was the major product after 8 h. This corresponds to the aforementioned results for 18 h incubation (Gondry et al. 2001).

Our results showed that **1a** and **2a** were very well converted to tetradehydrogenated products by Ndas_1146/7 in the biotransformation experiments and were among the well-accepted substrates in enzyme assays as well. In comparison, one of its natural substrates **11a** was a poor substrate, at least in biotransformation experiments (Table S2, Fig. S5).

Comparison of the acceptance of the 34 selected substrates in vitro by the three CDOs proved that CDO-Np showed much higher activities than AlbA/B, followed by Ndas_1146/7, which accepted only ten substrates with product yields of more than 5%. However, it cannot be excluded that this difference, at least in part, was caused by different CDO concentrations in the crude extracts, which were not possible to be determined in this study.

Structure elucidations of the products obtained from enzyme assays of the three CDOs with the 34 selected CDPs revealed their preference toward those containing at least one aromatic amino acid. This phenomenon could be explained by the fact that all three enzymes use phenylalanine- and tyrosine-containing CDPs as natural substrates. It seems that the aromatic character of the substrates is important for their acceptance. Almost all major didehydrogenated products in an enzyme assay carried a ∆Phe or ∆Tyr residue, while ∆Trp-containing didehydrogenated derivatives were only found as minor products. Several ∆Trp-containing tetradehydrogenated CDPs, like **3d**, **5d**, and **7d**, are major or main products of CDO-Np assays. This indicates that ∆Trp-containing didehydrogenated derivatives are more easily converted to tetradehydrogenated products or the dehydrogenation at the tryptophanyl site is the second dehydrogenation step. In the assays of **3a** and **5a** with AlbA/B and Ndas_1146/7, didehydrogenated products **3b** and **5b** were detected almost exclusively, indicating the difficulty for installation of a C–C bond at the tryptophanyl site by the two enzymes.

CDPs containing d-amino acids are poor substrates for the investigated CDOs. The 2,5-diketopierazine ring also seems to be essential for an acceptance by the three CDOs. Anthranilic acid–containing CDPs with a benzodiazepine dione skeleton (**35a** and **36a**) and compound **42a** with a 2,6-diketopiperazine core were not consumed. Methylation of one nitrogen (**43a**) at the DKP ring of *cyclo*-Phe-Phe (**30a**) led to the formation of di- instead of tetrahydrogenated derivative by CDO-Np. The dimethylated derivative **44a** was not converted at all (Figs. S4, S7, S33, S40, and S41).

The results presented in this study provide a solid basis for potential usage of CDOs in the biotechnology and synthetic biology for production of dehydrogenated CDPs. It will be interesting to find CDOs, which use exclusively CDPs comprising aliphatic amino acids and to test their substrate specificity as well as their behavior to install the C–C double bonds.

## Electronic supplementary material

ESM 1 (PDF 15.3 kb)
