# Supplementary material for: Comparative studies on similarities and differences of cyclodipeptide oxidases for installation of C–C double bonds at the diketopiperazine ring
Source: Appl Microbiol Biotechnol. 2020 Jan 27;104(6):2523–36. doi: 10.1007/s00253-020-10392-7 (PMC7044140; doi:10.1007/s00253-020-10392-7)
Supplement: Supplementary file 1 — (PDF 15.3 kb) [file 253_2020_10392_MOESM1_ESM.pdf]

Supporting Information

Comparative studies on similarities and differences of cyclodipeptide oxidases for installation of C-C double bonds at the diketopiperazine ring

Lena Mikulski,[1] Johanna Schäfer,[1] Kirsten Brockmeyer, Rixa Kraut, Shu-Ming Li*

Institut für Pharmazeutische Biologie und Biotechnologie, Philipps-Universität Marburg, Robert-Koch-Straße 4, 35037 Marburg, Germany

[1]These authors contributed equally to this study

Corresponding author:

*Tel/Fax: + 49-6421-28-22461/25365. E-mail: shuming.li@staff.uni-marburg.de.

ORCID Shu-Ming Li: 0000-0003-4583-2655

Table S1: Primers used in this study.

Name	Sequence (5´-3´)	Construct	Use
Np003-for	AGTGGCCATGGCTGTGGCGGCCGAAGAA	pKB78	Cloning vector CDO-Np
Np004b-rev	TGGATCCTCAGGCTGAAACGGCACCGG	pKB78	
Sn001-for	ACCATGGGGCGCCACCCATCGCATT	pKB89	Cloning vector AlbA/B
Sn001-rev	TGGATCCTCAGGGGGTTTCAATTTC	pKB89	

Table S2: Product yields obtained by biotransformation experiments.

CDP	CDO-Np -2H	CDO-Np -4H	CDO-Np total	AlbA/B -2H	AlbA/B -4H	AlbA/B total	Ndas_1146/7 -2H	Ndas_1146/7 -4H	Ndas_1146/7 total
	**Product yields in %**								
cyclo-L-Phe-L-Leu (**1a**)	25.3	63.4	88.6	31.7	59.5	91.2	15.2	82.2	97.5
cyclo-L-Phe-L-Tyr (**2a**)	10.0	69.4	79.4	21.2	36.3	57.5	11.6	85.5	97.1
cyclo-L-Trp-L-Tyr (**3a**)	55.8	12.0	67.8	57.6	13.7	71.3	3.0	24.5	27.5
cyclo-L-Trp-L-Phe (**5a**)	65.7	5.5	71.2	67.1	9.1	76.2	82.0	13.6	95.6
cyclo-L-Phe-L-His (**6a**)	18.9	69.9	88.8	27.0	65.1	92.1	22.5	67.2	89.6
cyclo-L-Trp-L-Leu (**7a**)	37.0	15.4	52.3	54.6	10.3	64.9	46.4	15.1	61.5
cyclo-D-Trp-L-Tyr (**8a**)	60.4	≤0.08	60.4	61.4	≤0.08	61.4	4.9	≤0.08	4.9
cyclo-L-Trp-L-Ala (**9a**)	19.2	0.8	20.0	26.8	≤0.23	26.8	32.9	≤0.23	32.9
cyclo-L-Trp-D-Ala (**10a**)	6.1	≤0.30	6.1	4.5	≤0.30	4.5	4.8	≤0.30	4.8
cyclo-D-Trp-L-Ala (**11a**)	7.8	≤0.24	7.8	4.8	≤0.24	4.8	7.1	≤0.24	7.1
cyclo-D-Trp-D-Ala (**12a**)	5.4	≤0.11	5.4	12.3	≤0.11	12.3	14.7	≤0.11	14.7
cyclo-L-Trp-L-His (**13a**)	34.8	19.6	54.4	26.3	48.7	75.0	22.2	62.4	84.6
cyclo-L-Phe-L-Ser (**14a**)	40.1	≤0.32	40.1	29.6	≤0.32	29.6	11.6	≤0.32	11.6
cyclo-L-Tyr-L-Ser (**15a**)	54.1	≤0.19	54.1	≤0.19	≤0.19	≤0.19	6.9	3.4	10.2
cyclo-L-Phe-L-Pro (**16a**)	22.9	≤0.17	22.9	36.3	≤0.17	36.3	11.4	≤0.17	11.4
cyclo-L-Tyr-L-Pro (**17a**)	43.6	≤0.28	43.6	15.8	≤0.28	15.8	15.8	≤0.28	15.8
cyclo-L-Trp-L-Pro (**18a**)	2.0	≤0.18	2.0	12.5	≤0.18	12.5	42.4	≤0.18	42.4
cyclo-L-Trp-D-Pro (**19a**)	0.8	≤0.13	0.8	0.55	≤0.13	0.55	5.1	≤0.13	5.1
cyclo-D-Trp-L-Pro (**20a**)	0.7	≤0.21	0.7	0.75	≤0.21	0.75	2.6	≤0.21	2.6
cyclo-D-Trp-D-Pro (**21a**)	≤0.03	≤0.03	≤0.03	0.89	≤0.03	0.89	2.3	≤0.03	2.3
cyclo-L-His-L-Ala (**24a**)	13.6	81.8	95.4	≤0.50	95.1	95.1	6.0	87.5	93.5
cyclo-L-Tyr-L-Tyr (**25a**)	20.5	45.7	66.2	26.5	43.8	70.3	14.5	44.6	59.1
cyclo-L-Tyr-Gly (**27a**)	62.9	≤0.63	62.5	64.5	≤0.63	64.5	12.6	≤0.63	12.6
cyclo-L-Phe-Gly (**28a**)	33.1	≤0.06	33.1	78.2	≤0.06	78.2	18.1	≤0.06	18.1
cyclo-L-Trp-L-Trp (**29a**)	32.4	≤0.11	32.4	54.8	3.6	58.4	47.3	3.1	50.4
cyclo-L-Phe-L-Phe (**30a**)	17.7	12.7	30.4	10.6	18.8	29.4	9.2	19.7	28.9
cyclo-L-Trp-Gly (**31a**)	4.5	≤0.38	4.5	3.9	≤0.38	3.9	≤0.38	≤0.38	≤0.38
cyclo-L-His-L-Pro (**32a**)	4.4	≤0.47	4.4	4.9	≤0.47	4.9	5.3	≤0.47	5.3
cyclo-L-Leu-L-Pro (**33a**)	2.0	≤0.15	2.0	0.58	≤0.15	0.58	0.7	≤0.15	0.7
cyclo-L-Val-L-Pro (**34a**)	≤0.19	≤0.19	≤0.19	≤0.19	≤0.19	≤0.19	≤0.19	≤0.19	≤0.19
cyclo-L-Trp-Ant (**35a**)	≤0.13	≤0.13	≤0.13	≤0.13	≤0.13	≤0.13	≤0.13	≤0.13	≤0.13
cyclo-D-Trp-Ant (**36a**)	≤0.13	≤0.13	≤0.13	≤0.13	≤0.13	≤0.13	≤0.13	≤0.13	≤0.13

Table S3: HR-ESI-MS data for cyclodipeptides and their dehydrogenated products.

Compound	Chemical Formula	$[M+H]^+$ Calculated [Da]	$[M+H]^+$ Measured [Da]	Deviation [ppm]
**1a**	$C_{15}H_{20}N_2O_2$	261.1598	261.1593	1.91
**1b**	$C_{15}H_{18}N_2O_2$	259.1441	259.1460	-7.33
**1c**	$C_{15}H_{18}N_2O_2$	259.1441	259.1454	-5.02
**1d**	$C_{15}H_{16}N_2O_2$	257.1285	257.1300	-5.83
**2a**	$C_{18}H_{18}N_2O_3$	311.1390	311.1383	2.25
**2b**	$C_{18}H_{16}N_2O_3$	309.1234	309.1224	3.23
**2c**	$C_{18}H_{16}N_2O_3$	309.1234	309.1227	2.26
**2d**	$C_{18}H_{14}N_2O_3$	307.1077	307.1094	-5.54
**3a**	$C_{20}H_{19}N_3O_3$	350.1499	350.1478	6.00
**3b**	$C_{20}H_{17}N_3O_3$	348.1343	348.1357	-4.02
**3d**	$C_{20}H_{15}N_3O_3$	346.1186	346.1191	-1.44
**4a**	$C_{25}H_{27}N_3O_3$	418.2125	418.2145	-4.78
**4b**	$C_{25}H_{25}N_3O_3$	416.1969	416.1998	-6.97
**4c**	$C_{25}H_{25}N_3O_3$	416.1969	416.2003	-8.17
**4d**	$C_{25}H_{23}N_3O_3$	414.1812	414.1847	-8.45
**5a**	$C_{20}H_{19}N_3O_2$	334.1550	334.1556	-1.80
**5b**	$C_{20}H_{17}N_3O_2$	332.1394	332.1412	-5.42
**5d**	$C_{20}H_{15}N_3O_2$	330.1237	330.1227	3.03
**6a**	$C_{15}H_{16}N_4O_2$	285.1346	285.1354	-2.81
**6c**	$C_{15}H_{14}N_4O_2$	283.1190	283.1198	-2.83
**6d**	$C_{15}H_{12}N_4O_2$	281.1033	281.1036	-1.07
**7a**	$C_{17}H_{21}N_3O_2$	300.1707	300.1715	-2.67
**7b**	$C_{17}H_{19}N_3O_2$	298.1550	298.1553	-1.01
**7d**	$C_{17}H_{17}N_3O_2$	296.1394	296.1400	-2.03
**8a**	$C_{20}H_{19}N_3O_3$	350.1499	350.1505	-1.71
**8b**	$C_{20}H_{17}N_3O_3$	348.1343	348.1352	-2.59
**9a**	$C_{14}H_{15}N_3O_2$	258.1237	258.1246	-3.49
**9b**	$C_{14}H_{13}N_3O_2$	256.1081	256.1090	-3.51
**9d**	$C_{14}H_{11}N_3O_2$	254.0924	254.0932	-3.15
**10a**	$C_{14}H_{15}N_3O_2$	258.1237	258.1233	1.55
**10b**	$C_{14}H_{13}N_3O_2$	256.1081	256.1066	5.86
**10c**	$C_{14}H_{13}N_3O_2$	256.1081	256.1071	3.90
**11a**	$C_{14}H_{15}N_3O_2$	258.1237	258.1230	2.71
**11b**	$C_{14}H_{13}N_3O_2$	256.1081	256.1064	6.64
**11c**	$C_{14}H_{13}N_3O_2$	256.1081	256.1067	5.47
**12a**	$C_{14}H_{15}N_3O_2$	258.1237	258.1240	-1.16
**12b/c**	$C_{14}H_{13}N_3O_2$	256.1081	256.1062	7.42
**13a**	$C_{17}H_{17}N_5O_2$	324.1455	324.1445	3.09
**13b**	$C_{17}H_{15}N_5O_2$	322.1299	322.1303	-1.24
**13d**	$C_{17}H_{13}N_5O_2$	320.1142	320.1140	0.62
**14a**	$C_{12}H_{14}N_2O_3$	235.1077	235.1070	2.98
**14c**	$C_{12}H_{12}N_2O_3$	233.0921	233.0913	3.43
**15a**	$C_{12}H_{14}N_2O_4$	251.1026	251.1025	0.40
**15c**	$C_{12}H_{12}N_2O_4$	249.0870	249.0869	0.40
**16a**	$C_{14}H_{16}N_2O_2$	245.1285	245.1288	-1.22
**16b**	$C_{14}H_{14}N_2O_2$	243.1128	243.1118	4.11
**16c**	$C_{14}H_{14}N_2O_2$	243.1128	243.1112	6.58
**16d**	$C_{14}H_{12}N_2O_2$	241.0972	241.0973	-0.41
**17a**	$C_{14}H_{16}N_2O_3$	261.1234	261.1236	-0.77
**17b**	$C_{14}H_{14}N_2O_3$	259.1077	259.1063	5.40
**17c**	$C_{14}H_{14}N_2O_3$	259.1077	259.1066	4.25
**17d**	$C_{14}H_{12}N_2O_3$	257.0921	257.0918	1.17
**18a**	$C_{16}H_{17}N_3O_2$	284.1394	284.1395	-3.87
**18b**	$C_{16}H_{15}N_3O_2$	282.1237	282.1230	2.48
**18d**	$C_{16}H_{13}N_3O_2$	280.1081	280.1060	7.50

Table S3: HR-ESI-MS data for cyclodipeptides and their dehydrogenated products. Continued.

**19a**	$C_{16}H_{17}N_3O_2$	284.1394	284.1400	-5.63
**19b/c**	$C_{16}H_{15}N_3O_2$	282.1237	282.1259	-7.80
**20a**	$C_{16}H_{17}N_3O_2$	284.1394	284.1392	-2.82
**20b**	$C_{16}H_{15}N_3O_2$	282.1237	282.1223	7.96
**20c**	$C_{16}H_{15}N_3O_2$	282.1237	282.1226	3.90
**21a**	$C_{16}H_{17}N_3O_2$	284.1394	284.1398	-4.93
**21b/c**	$C_{16}H_{15}N_3O_2$	282.1237	282.1224	4.61
**22a**	$C_{21}H_{25}N_3O_2$	352.2020	352.2027	-1.99
**23a**	$C_{21}H_{25}N_3O_2$	352.2020	352.2029	-2.56
**23b**	$C_{21}H_{23}N_3O_2$	350.1863	350.1861	0.57
**24a**	$C_9H_{12}N_4O_2$	209.1033	209.1016	8.13
**24b/c**	$C_9H_{10}N_4O_2$	207.0877	207.0892	-7.24
**24d**	$C_9H_8N_4O_2$	205.0720	205.0705	7.13
**25a**	$C_{18}H_{18}N_2O_4$	327.1339	327.1337	0.61
**25b/c**	$C_{18}H_{16}N_2O_4$	325.1183	325.1171	3.69
**25d**	$C_{18}H_{14}N_2O_4$	323.1026	323.1027	-0.31
**26a**	$C_{23}H_{26}N_2O_4$	395.1965	395.1983	-4.55
**26b**	$C_{23}H_{24}N_2O_4$	393.1809	393.1807	0.51
**26c**	$C_{23}H_{24}N_2O_4$	393.1809	393.1806	0.76
**26d**	$C_{23}H_{22}N_2O_4$	391.1652	391.1657	-1.28
**27a**	$C_{11}H_{12}N_2O_3$	221.0921	221.0904	7.69
**27b**	$C_{11}H_{10}N_2O_3$	219.0764	219.0778	-6.39
**28a**	$C_{11}H_{12}N_2O_2$	205.0972	205.0970	0.98
**28b**	$C_{11}H_{10}N_2O_2$	203.0815	203.0814	0.49
**29a**	$C_{22}H_{20}N_4O_2$	373.1659	373.1657	0.54
**29b**	$C_{22}H_{18}N_4O_2$	371.1503	371.1500	0.81
**29d**	$C_{22}H_{16}N_4O_2$	369.1346	369.1337	2.44
**30a**	$C_{18}H_{18}N_2O_2$	295.1441	295.1444	-1.02
**30b**	$C_{18}H_{16}N_2O_2$	293.1285	293.1270	5.12
**30d**	$C_{18}H_{14}N_2O_2$	291.1128	291.1134	-2.06
**31a**	$C_{13}H_{13}N_3O_2$	244.1081	244.1086	-2.05
**31b**	$C_{13}H_{11}N_3O_2$	242.0924	242.0937	-5.37
**32a**	$C_{11}H_{14}N_4O_2$	235.1190	235.1187	1.28
**32b/c**	$C_{11}H_{12}N_4O_2$	233.1033	233.1052	-3.43
**33a**	$C_{11}H_{18}N_2O_2$	211.1441	211.1448	-3.32
**33b/c**	$C_{11}H_{16}N_2O_2$	209.1285	209.1279	2.87
**34a**	$C_{10}H_{16}N_2O_2$	197.1285	197.1278	3.55
**35a**	$C_{18}H_{15}N_3O_2$	306.1237	306.1248	-3.59
**35b**	$C_{18}H_{13}N_3O_2$	304.1084	304.1068	5.26
**36a**	$C_{18}H_{15}N_3O_2$	306.1237	306.1244	-2.29
**37a**	$C_{18}H_{16}D_2N_2O_3$	313.1516	313.1520	-1.28
**37b**	$C_{18}H_{14}D_2N_2O_3$	311.1359	311.1340	6.11
**37c**	$C_{18}H_{15}DN_2O_3$	310.1296	310.1311	-4.84
**37d**	$C_{18}H_{13}DN_2O_3$	308.1140	308.1156	-5.19
**38a**	$C_{20}H_{17}D_2N_3O_3$	352.1625	352.1636	-3.12
**38b**	$C_{20}H_{16}DN_3O_3$	349.1405	349.1422	-4.87
**38d**	$C_{20}H_{14}DN_3O_3$	347.1249	347.1250	-0.29
**39a**	$C_{16}H_{19}N_3O_2$	286.1550	286.1554	-1.40
**39b**	$C_{16}H_{17}N_3O_2$	284.1394	284.1375	6.69
**40a**	$C_{17}H_{21}N_3O_2$	300.1707	300.1708	-0.33
**40b**	$C_{17}H_{19}N_3O_2$	298.1550	298.1549	0.34
**40c**	$C_{17}H_{19}N_3O_2$	298.1550	298.1546	1.34
**41a**	$C_{22}H_{29}N_3O_2$	368.2333	368.2344	-2.99
**41b**	$C_{22}H_{27}N_3O_2$	366.2176	366.2173	0.82
**41d**	$C_{22}H_{25}N_3O_2$	364.2020	364.2013	1.92
**42a**	$C_{18}H_{18}N_2O_2$	295.1441	295.1444	-1.02
**43a**	$C_{19}H_{20}N_2O_2$	309.1598	309.1595	0.97
**43b**	$C_{19}H_{18}N_2O_2$	307.1441	307.1442	-0.33
**43d**	$C_{19}H_{16}N_2O_2$	305.1285	305.1273	3.93
**44a**	$C_{20}H_{22}N_2O_2$	323.1754	323.1755	-0.31

Table S4: ^{1}H NMR data of *cyclo*ΔLeu-L-Phe (**1b**), *cyclo*-L-Leu-ΔPhe (**1c**) and *cyclo*-ΔLeu-ΔPhe (**1d**) isolated from an enzyme assay with CDO-Np.

	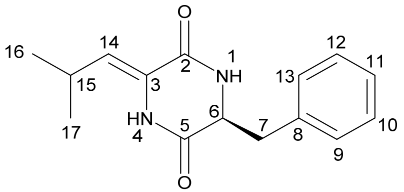	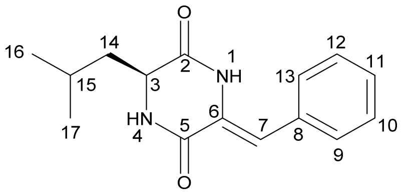	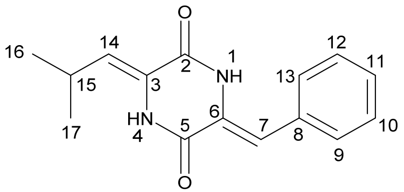
Position	*Cyclo*-ΔLeu-L-Phe (**1b**)<br>DMSO-d$_6$, 500 MHz<br>multi., (*J* [Hz])	*Cyclo*-L-Leu-ΔPhe (**1c**)<br>DMSO-d$_6$, 500 MHz<br>multi., (*J* [Hz])	*Cyclo*-ΔLeu-ΔPhe (**1d**)<br>DMSO-d$_6$, 500 MHz<br>multi., (*J* [Hz])
1	8.20, d, (1.9)	9.90, br s	10.25, br, s
3	-	3.93, m	-
4	9.68, br s	8.50, br s	10.25, br, s
6	4.29, ddd, (5.0, 4.0, 2.5)	-	-
7	3.11, dd, (13.7, 4.0)<br>2.90, dd, (13.7, 5.0)	6.66, s	6.71, s
9	7.11, m	7.50, d, (7.3)	7.53, d, (7.5)
10	7.22, m[a]	7.39, t, (7.7)	7.40, t, (7.5)
11	7.19, m[a]	7.29, t, (7.2)	7.30, t, (7.5)
12	7.22, m[a]	7.39, t, (7.7)	7.40, t, (7.5)
13	7.11, m	7.50, d, (7.3)	7.53, d, (7.5)
14	5.26, d, (10.3)	1.61, dd, (13.8, 5.7)<br>1.57, dd, (13.8, 6.6)	5.69, d, (10.4)
15	2.62,dsept, (10.3, 6.5)	1.81, sept, (6.7)	2.96, dsept, (10.4, 6.6)
16	0.83, d, (6.5)	0.89, d, (6.7)	0.98, d, (6.6)
17	0.70, d, (6.5)	0.90, d, (6.7)	0.98, d, (6.6)

[a] signals overlapping with each other. The data corresponded well to those published previously (Kanzaki et al. 2000).

Table S5: ^{1}H NMR data of *cyclo*-ΔPhe-L-Tyr (**2b**), *cyclo*-L-Phe-ΔTyr (**2c**), and *cyclo*-ΔPhe-ΔTyr (**2d**) isolated from an enzyme assay with CDO-Np.

	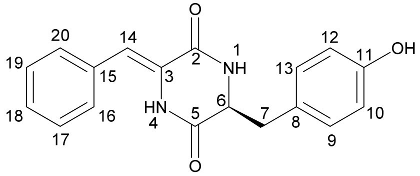	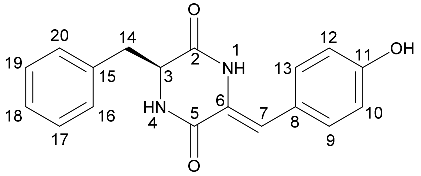	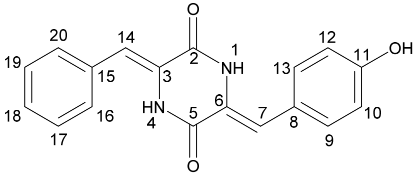
Position	*Cyclo*-ΔPhe-L-Tyr (**2b**)<br>DMSO-d_6, 500 MHz<br>multi., (*J* [Hz])	*Cyclo*-L-Phe-ΔTyr (**2c**)<br>DMSO-d_6, 500 MHz<br>multi., (*J* [Hz])	*Cyclo*-ΔPhe-ΔTyr (**2d**)<br>DMSO-d_6, 500 MHz<br>multi., (*J* [Hz])
1	8.35, d, (2.5)	9.52, br s	10.03, br s
3	-	4.31, ddd, (5.1, 4.4, 2.8)	-
4	9.10, s	8.29,d, (2.6)	9.82, br s
6	4.26, m	-	-
7	3.03, dd, (13.8, 4.0)<br>2.83, dd, (13.8, 5.0)	6.29, s	6.67, s
9	6.95, dt, (8.9, 2.5)	7.10, dt, (8.6, 2.3)	7.43, d, (8.4)^{b}
10	6.60, dt, (8.9, 2.5)	6.73, dt, (8.6, 2.5)	6.80, dt, (8.4, 2.4)
11-OH	9.64, br s	9.63, s	-
12	6.60, dt, (8.9, 2.5)	6.73, dt, (8.6, 2.5)	6.80, dt, (8.4, 2.4)
13	6.95, dt, (8.9, 2.5)	7.10, dt, (8.6, 2.3)	7.43, d, (8.4) ^{b}
14	6.36, s	3.12, dd, (13.6. 4.4)<br>2.95, dd, (13.6, 5.1)	6.70, s
16	7.24, d, (7.0)^{a}	7.17, m	7.57, d, (7.5)
17	7.34, t, (7.3)	7.21, t, (7.2)	7.40, t, (7.6) ^{b}
18	7.25, t, (7.3)^{a}	7.17, m	7.30, t, (7.6)
19	7.34, t, (7.3)	7.21, t, (7.2)	7.40, t, (7.6) ^{b}
20	7.24, d, (7.0)^{a}	7.17, m	7.57, d, (7.5)

$^{a, b}$ signals overlapping with each other.

Table S6: ^{1}H NMR data of *cyclo*-L-Trp-ΔTyr (**3b**) isolated from an enzyme assay with AlbA/B and *cyclo*-ΔTrp-ΔTyr (**3d**), *cyclo*-L-Trp-ΔPhe (**5b**), and *cyclo*-ΔTrp-ΔPhe (**5d**) isolated from enzyme assays with CDO-Np.

	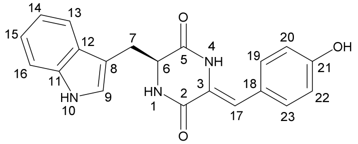	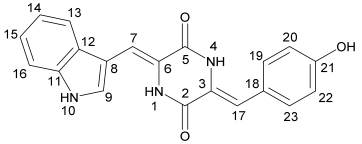	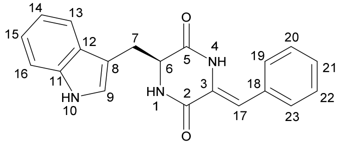	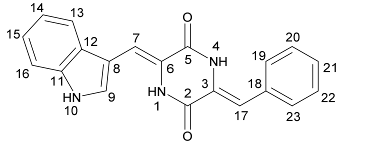
Position	*cyclo*-L-Trp-ΔTyr (**3b**) DMSO-d_6, 500 MHz multi., (*J* [Hz])	*cyclo*-ΔTrp-ΔTyr (**3d**) DMSO-d_6, 500 MHz multi., (*J* [Hz])	*cyclo*-L-Trp-ΔPhe (**5b**) DMSO-d_6, 500 MHz multi., (*J* [Hz])	*cyclo*-ΔTrp-ΔPhe (**5d**) DMSO-d_6, 500 MHz multi., (*J* [Hz])
1	8.22, d, (2.9)	9.73, br s	8.34, d, (2.9)	9.77, br s
4	9.17, s	9.73, br s	9.34, s	9.77, br s
6	4.23, dd, (4.7, 3.9)	-	4.26, m	-
7	3.33, dd, (14.6, 3.9)<br>3.02, dd, (14.6, 4.7)	7.07, s	3.37, dd, (14.6, 3.5)<br>3.03, dd, (14.6, 4.7)	7.12, s
9	7.01, d, (2.0)	8.08, s	7.00, d, (2.5)	8.10, d, (1.9)
10	10.81, d, (1.8)	11.71, s	10.83, s	11.76, s
13	7.54, d, (7.2)	7.68, dt, (8.0, 0.8)	7.54, d, (7.0)	7.69, d, (8.0)
14	6.99, td, (7.0, 1.2)	7.13, ddd, (8.4, 7.6, 1.0)	7.01, td, (7.0, 1.4)	7.14, ddd, (8.4, 7.5, 1.0)
15	7.02, td,(7.1, 1.5)	7.19, ddd, (8.4, 7.6, 1.1)	7.04, td, (7.0, 1.5)	7.20, ddd, (8.4, 7.5, 1.2)
16	7.17, d, (7.5)	7.44, dt, (8.0, 0.8)[a]	7.16, m	7.45, dt, (8.0, 0.8)[b]
17	6.16, s	6.68, s	6.19, s	6.76, s
19	6.54, m	7.43, d, (8.7)[a]	6.58, m	7.56, d, (7.4)
20	6.54, m	6.82, dt, (8.7, 2.4)	7.12, m	7.42, t, (7.4)[b]
21	9.49, s	9.73, br s	7.12, m	7.33, tt, (7.4, 1.2)
22	6.54, m	6.82, dt, (8.7, 2.4)	7.12, m	7.42, t, (7.4)[b]
23	6.54, m	7.43, d, (8.7)[a]	6.55, d, (7.6)	7.56, d, (7.4)

[a,b] signals with same letter are overlapping with each other.

Table S7: $[M+H]^+$ fragmentation ions of CDPs and their didehydrogenated products obtained by ESI-CID-MS/MS.

Compounds		Type of ions $[M+H]^+$									
		A	B	C	D	E	F	G	G-2H	H	I
cFL (**1a**)	Calculated	261.1598	233.1648	216.1383	188.1434	132.0808; 98.0964	120.0808; 86.0964	205.0972; 171.1128	203.0815; 169.0972	131.0491; 97.0648	91.0542
	Observed	261.1593	233.1644	216.1379	188.1441	-	120.0795	-	-	-	-
cFΔL(**1b**)	Calculated	259.1441	231.1492	214.1226	186.1277	132.0808; 96.0808	120.0808; 84.0808	205.0972; 169.0972	203.0815; 167.0815	131.0491; 95.0491	91.0542
	Observed	259.1460	231.1509	214.1230	-	-	120.0832	-	-	-	91.0563
cΔFL (**1c**)	Calculated	259.1441	231.1492	214.1226	186.1277	130.0651; 98.0964	118.0651; 86.0964	203.0815; 171.1128	201.0659; 169.0972	129.0335; 97.0648	91.0542
	Observed	259.1454	231.1514	-	-	-	118.0670	203.1547	-	-	91.0562
cFY (**2a**)	Calculated	311.1390	283.1441	266.1176	238.1213	132.0808; 148.0757	120.0808; 136.0750	205.0975; 221.0921	203.0815; 219.0764	131.0491; 147.0441	91.0542; 107.0491
	Observed	311.1383	283.1426	266.1148	238.1213	-	120.0788; 136.0750	205.0968	-	-	107.0464
cΔFY (**2b**)	Calculated	309.1234	281.1255	264.1019	236.1070	130.0651; 148.0757	118.0651; 136.0750	203.0815; 221.0921	201.0659; 219.0764	129.0335; 147.0441	91.0542; 107.0491
	Observed	309.1224	281.1247	-	-	-	118.0643; 136.0761	203.0811	-	-	91.0546; 107.0492
cFΔY (**2c**)	Calculated	309.1234	281.1255	264.1019	236.1070	132.0808; 146.0600	120.0808; 134.0600	205.0975; 219.0764	203.0815; 217.0608	131.0491; 145.0284	91.0542; 107.0491
	Observed	309.1227	281.1274	-	-	146.0597	120.0803; 134.0601	-	-	-	91.0534; 107.0490
cWY (**3a**)	Calculated	350.1499	322.1550	305.1285	277.1335	171.0917; 148.0757	159.0917; 136.0750	244.1081; 221.0921	242.0924; 219.0764	170.0600; 147.0441	130.0651; 107.0491
	Observed	350.1478	322.1570	-	-	-	159.0918	221.0923	219.0756	170.0597	130.0636
cWΔY (**3b**)	Calculated	348.1343	320.1394	303.1128	275.1179	171.0917; 146.0600	159.0917; 134.0600	244.1081; 219.0764	242.0924; 217.0608	170.0600; 145.0284	130.0651; 107.0491
	Observed	348.1357	-	-	-	-	-	219.0762	217.0611	170.0623	130.0652
2-*tert*-prenyl-cWY(**4a**)	Calculated	418.2125	390.2176	373.0045	345.1961	239.1543; 148.0757	227.1543; 136.0757	312.1708; 221.0921	310.1550; 219.0764	238.1226; 147.0441	198.1277; 107.0491
	Observed	418.,2145	-	-	-	-	136.0769	221.0913	219.0768	-	198.1297
2-*tert*-prenyl-cWΔY (**4c**)	Calculated	416.1969	388.2020	371.1754	243.1804	239.1543; 146.0600	227.1543; 134.0600	312.1707; 219.0764	310.1550; 217.0608	236.1070; 147.0441	198.1277; 107.0491
	Observed	416.2001	-	-	-	-	-	219.0815	217.0622	-	198.1295
cWF (**5a**)	Calculated	334.1550	306.1601	289.1335	261.1386	171.0917; 132.0808	159.0917; 120.0808	244.1081; 205.0972	242.0924; 203.0815	170.0600; 131.0491	130.0651; 91.0542
	Observed	334.1556	306.1576	289.1355	-	-	159.0910; 120.0797	205.0945	203.0802	170.0593	130.0643
cWΔF (**5b**)	Calculated	332.1394	304.1444	287.1179	259.1230	171.0917; 130.0651	159.0917; 118.0651	244.1081; 203.0815	242.0924; 201.0659	170.0600; 129.0335	130.0651; 91.0542
	Observed	332.1412	-	-	-	130.0649	118.0651	203.0819	201.0662	170.0601	130.0649
cFH (**6a**)	Calculated	285.1346	257.1397	240.1131	212.1182	132.0808; 122.0713	120.0808; 110.0713	205.0945; 195.0877	203.0815; 193.0720	131.0491; 121.0396	91.0542; 81.0447
	Observed	285.1354	257.1391	-	212.1171	-	120.0796; 110.0704	195.0896	-	-	-
cΔFH (**6c**)	Calculated	283.1190	255.1240	238.0975	210.1026	130.0651; 122.0713	118.0651; 110.0713	203.0815; 195.0877	201.0659; 193.0720	129.0335; 121.0396	91.0542; 81.0447
	Observed	283.1190	255.1225	238.0979	210.1015	-	118.0643; 110.0705	203.0807	-	-	-
cWL (**7a**)	Calculated	300.1707	272.1757	255.1492	227.3154	171.0917; 98.0964	159.0910; 86.0964	244.1081; 171.1128	242.0924; 169.0972	170.0600; 97.0648	130.0651; 57.1155
	Observed	300.1715	272.1766	-	-	-	159.0919	171.1077	169.0978	170.0604	130.0643
cWΔL (**7b**)	Calculated	298.1550	270.1601	253.1335	225.1386	171.0917; 96.0808	159.0910; 84.0808	244.1081; 169.0972	242.0924; 167.0815	170.0600; 95.0491	130.0651; 57.1155
	Observed	298.1553	-	-	-	-	-	169.0981	167.0825	170.0619	130.0648
cDWY (**8a**)	Calculated	350.1499	322.1550	305.1285	277.1335	171.0917; 148.0757	159.0917; 136.0750	244.1081; 221.0921	242.0924; 219.0764	170.0600; 147.0441	130.0651; 107.0491
	Observed	350.1514	322.1555	305.1299	-	-	159.0912	221.0905	219.0759	170.0601	130.0643
cDWΔY (**8b**)	Calculated	348.1343	320.1394	303.1128	275.1179	171.0917; 146.0600	159.0917; 134.0600	244.1081; 219.0764	242.0924; 217.0608	170.0600; 145.0284	130.0651; 107.0491
	Observed	348.1352	-	-	-	-	-	219.0753	217.0600	170.0605	130.0654
***cyclo*-Trp-Ala**	Calculated	258.1237	230.1288	213.1022	185.1073	171.0917	159.0917	244.1081; 129.0659	242.0924; 127.0502	170.0600	130.0651
cWA (**9a**)	Observed	258.1246	230.1297	-	-	-	159.0914	-	-	170.0599	130.0640
cWDA (**10a**)	Observed	258.1234	-	213.0943	-	171.0917	159.0935	-	242.0912	170.0621	130.0653
cDWA (**11a**)	Observed	258.1235	230.1312	213.1125	-	171.0642	159.0918	-	-	170.0617	130.0652
cDWDA (**12a**)	Observed	258.1121	230.1204	213.1204	-	-	159.0930	-	-	170.0604	130.0650
***cyclo*-TrpΔAla**	Calculated	256.1081	228.1131	211.0866	183.0917	171.0917	159.0917	244.1081; 127.0502	242.0924; 125.0346	170.0600	130.0651
cWΔA (**9b**)	Observed	256.1090	228.1107	-	-	-	-	127.0486	-	170.0603	130.0646
cWΔA (**10b**)	Observed	256.1043	-	-	183.0930	-	-	-	-	-	130.0649
cDWΔA (**11b**)	Observed	256.1064	-	-	-	-	-	-	-	-	130.0649
cDWΔA (**12b**	Observed	256.1065	-	-	-	-	159.0878	-	-	170.0504	130.0647
***cyclo*ΔTrp-Ala**	Calculated	256.1081	228.1131	211.0866	183.0917	169.0760	157.0760	242.0924; 129.0659	240.0768; 127.0502	168.0443	130.0651
cΔWDA (**10c**)	Observed	256.1060	-	-	-	-	157.0760	-	-	-	130.0642
cΔWA (**11c**)	Observed	256.1056	-	-	-	-	157.0745	-	-	-	130.0642
cWH (**13a**)	Calculated	324.1455	296.1506	279.1240	252.3205	171.0917, 122.0713	159.0917; 110.0713	244.1081; 195.0877	242.0924; 193.0720	170.0600; 121.0396	130.0651; 81.0447
	Observed	324.1445	-	-	-	-	159.0928	195.0868	-	170.0590	130.0635

Fragments smaller than 90 Da are not displayed.

Table S7: $[M+H]^+$ fragmentation ions of CDPs and their didehydrogenated products obtained by ESI-CID-MS/MS. Continued.

cWΔH (**13b**)	Calculated	322.1299	294.1349	277.1084	350.1213	171.0917	159.0917	244.1081; 193.0720	242.0924	170.0600	130.0651
	Observed	322.1303	-	-	-	-	-	-	-	-	130.0657
cFS (**14a**)	Calculated	235.1077	207.1128	190.0863	162.0913	132.0808	120.0808	205.0972; 145.0608	203.0815; 143.0451	131.0491	91.0542
	Observed	235.1070	207.1118	-	162.0909	-	120.0792	-	-	-	-
cΔFS (**14c**)	Calculated	233.0921	205.0972	188.0706	160.0757	130.0651	118.0651	203.0815; 145.0608	201.0659; 143.0451	129.0335	91.0542
	Observed	233.0913	205.0973	-	160.0766	-	118.0633	203.0813	-	-	-
cYS (**15a**)	Calculated	251.1026	223.1077	206.0812	178.0863	148.0757	136.0750	221.0921; 145.0608	219.0764; 143.0451	147.0441	107.0491
	Observed	251.1025	223.1054	-	178.0858	-	136.0735	145.0601	-	-	107.0476
cΔYS (**15c**)	Calculated	249.0870	221.0921	204.0655	176.0706	146.0600	134.0600	219.0764; 145.0608	217.0608; 143.0451	145.0284	107.0491
	Observed	249.0869	221.0870	-	-	-	134.0578	219.0751	-	-	107.0474
cFP (**16a**)	Calculated	245.1285	217.1335	200.1070	172.1121	132.0808	120.0808	205.0972; 155.0815	203.0815; 153.0659	131.0491	91.0542
	Observed	245.1288	217.1330	200.1066	172.1121	-	120.0811	155.0747	153.0656	131.0488	-
cFΔP (**16b**)	Calculated	243.1128	215.1179	198.0913	170.0964	132.0808	120.0808	205.0972; 153.0659	203.0815; 151.0502	131.0491	91.0542
	Observed	243.1118	215.1169-	-	-	-	-	153.0597	151.0490	-	-
cΔFP (**16c**)	Calculated	243.1128	215.1179	198.0913	170.0964	130.0651	118.0651	203.0815; 155.0815	201.0659; 153.0659	129.0335	91.0542
	Observed	243.1112	215.1157-	-	-	-	118.0614	-	-	-	-
cYP (**17a**)	Calculated	261.1234	233.1285	216.1019	188.1070	148.0757	136.0750	221.0921; 155.0815	219.0764; 153.0659	147.0441	107.0491
	Observed	261.1236	233.1280	-	188.1046	-	136.0737	155.0800	153.0652	147.0420	107.0468
cYΔP (**17b**)	Calculated	259.1077	231.1128	214.0863	186.0913	148.0757	136.0750	221.0921; 153.0659	219.0764; 151.0502	147.0441	107.0491
	Observed	259.1053	-	-	-	-	-	153.0637	-	-	107.0479
cΔYP (**17c**)	Calculated	259.1077	231.1128	214.0863	186.0913	146.0600	134.0600	219.0764; 155.0815	217.0608; 153.0659	145.0284	107.0491
	Observed	259.1066	231.1123	-	-	146.0602	134.0573	-	-	-	107.0468
***cyclo*-Trp-Pro**	Calculated	284.1394	256.1444	239.1179	211.1230	171.0917	159.0917	244.1081; 155.0815	242.0924; 153.0659	170.0600	130.0651
cWP (**18a**)	Observed	284.1381	-	-	-	-	159.0910	155.0818	153.0637	170.0607	130.0652
cWDP (**19a**)	Observed	284.1382	256.1435	239.1177	-	171.0642	159.0913	155.0808	153.0653	170.0611	-
cDWP (**20a**)	Observed	284.1379	256.1402	239.1178	-	171.0635	159.0915	155.0798	153.0651	170.0605	130.0653
cDWDP (**21a**)	Observed	284.1383	256.1378	239.1158	-	171.0624	159.0916	155.0804	153.0651	170.0603	130.0651
***cyclo*-TrpΔPro**	Calculated	282.1237	254.1288	237.1022	209.1073	171.0917	159.0917	244.1081; 153.0659	242.0924; 151.0502	170.0600	130.0651
cWΔP (**18b**)	Observed	282.1248	-	-	-	-	-	153.0649	151.0495	-	130.0643
cDWΔP (**21b**)	Observed	282.1127	-	-	-	-	-	153.0653	-	170.0682	130.0640
***cyclo*-ΔTrp-Pro**	Calculated	282.1237	254.1288	237.1022	209.1073	169.0760	157.0760	242.0924; 155.0815	240.0767; 153.0659	168.0443	130.0651
cΔWP (**20c**)	Observed	282.1133	-	-	-	-	159.0903	155.0638	153.0813	170.0593	130.0649
N1-prenyl-cWP (**23a**)	Calculated	352.2020	324.207	307.1804	279.1855	239.1543	227.1543	312.1707; 155.0815	310.1550; 153.0815	238.1226	198.1277
	Observed	352.2021	-	-	-	239.1186	-	155.0785	153.0650	-	198.1282
N1-prenyl-cWΔP (**23b**)	Calculated	350.1863	322.1914	305.1648	277.1698	239.1543	227.1543	312.1707; 153.0659	310.1550; 151.0502	238.1226	198.1277
	Observed	350.1800	-	-	-	-	-	153.0659			198.1272
O-prenyl-cYY (**26a**)	Calculated	395.1965	367.2016	350.1750	322.1801	148.0757; 203.1305	136.0757; 204.1383	221.0921; 289.1547	219.0764; 287.1390	147.0441; 215.1067	107.0491; 175.1117
	Observed	395.1951	367.3031	-	-	148.0766	136.0755; 204.1369	221.0902; 289.1564	219.0737	147.0621; 215.1037	107.0504; 175.1108
O-prenyl-cYΔY (**26b**)	Calculated	393.1809	365.1860	348.1594	320.1645	146.0600; 203.1305	134.0600; 204.1369	219.0764; 289.1547	217.0608; 287.1390	145.0284; 215.1067	107.0491; 175.1117
	Observed	393.1808	365.1804	-	-	-	-	219.0756	217.0580	-	107.0488; 175.1121
O-prenyl-cΔYY (**26c**)	Calculated	393.1809	365.1860	348.1594	320.1645	148.0757; 203.1305	136.0794	221.0921; 287.1547	219.0764; 285.1234	147.0441; 213.0910	107.0961; 175.1117
	Observed	393.1822	-	-	-		136.0794	-	219.0713; 285.2779	-	107.0492; 175.1163
cWDV (**39a**)	Calculated	286.1550	258.1601	241.1335	213.1386	171.0917	159.0917	244.1081; 157.0972	242.0924; 155.0815	170.0600	130.0651
	Observed	286.1540	-	-	-	171.0626	159.0922	157.0965	155.0810	170.0601	130.0654
cΔWDV (**39b**)	Calculated	284.1394	256.1444	239.1178	211.1229	169.0760	157.0760	242.0924; 157.0972	240.0768; 155.0815	170.0600	130.0651
	Observed	284.1327	-	-	-	169.0867	157.0757		155.0591	170.0895	130.0636
chWDV (**40a**)	Calculated	300.1707	272.1757	255.1491	227.1542	185.1073	173.1073	258.1237; 157.0972	256.1081; 155.0815	184.0757	144.0808
	Observed	300.1707	272.1727	-	-	185.0864	173.1044	157.0906	155.0619	184.0869	144.0812
chΔWDV (**40b**)	Calculated	298.1550	270.1601	253.1335	225.1386	183.0917	171.0917	256.1081; 157.0972	254.0924; 155.0815	184.0757	144.0808
	Observed	298.1588	-	-	-	-	171.0895	-	-	-	-
chWΔV (**40c**)	Calculated	298.1550	270.1492	253.1335	225.1286	185.1073	173.1073	258.1237; 155.0815	256.1081; 153.0659	182.0600	144.0808
	Observed	298.1499	-	-	-	-	-	-	153.1025	-	144.0793

Fragments smaller than 90 Da are not displayed.

Table S7: [M+H]$^{+}$ fragmentation ions of CDPs and their didehydrogenated products obtained by ESI-CID-MS/MS. Continued.

5-prenyl-chWDV (**41a**)	Calculated	368.2333	340.2383	323.2117	295.2168	253.1699	241.1699	326.1863; 157.0972	324.1707; 155.0815	252.1383; 97.0648	212.1434
	Observed	368.2349	-	-	295.1449	-	241.0957	157.0887	155.1136	252.1353	212.2434
5-prenyl-chΔWDV (**41b**)	Calculated	366.2176	338.2227	321.1961	293.2011	251.1543	239.5143	324.1707; 157.0972	322.1550; 155.0815	252.1383; 97.0648	212.1434
	Observed	366.2229	-	-	-	-	-	157.0950	155.0805	250.1218	-
N-methyl-cFF (**43a**)	Calculated	309.1598	281.1648	250.1226	222.1277	132.0808; 133.0866	134.0964; 120.0794	219.1128; 219.1128	217.0972; 217.0972	131.0491; 131.0491	91.0542; 91.0542
	Observed	309.1593	281.1634	-	-	-	134.0952; 120.0794	219.1191	217.0981	131.0641	-
N-methyl-cFΔF (**43b**)	Calculated	307.1441	279.1492	248.1069	220.1120	132.0808; 133.0866	134.0964; 118.0651	219.1128; 217.0972	217.0972; 215.0815	131.0491; 131.0491	91.0542; 91.0542
	Observed	307.1436	279.1487	-	-		134.0950	219.1094; 217.0940	215.0806	-	-

Fragments smaller than 90 Da are not displayed.

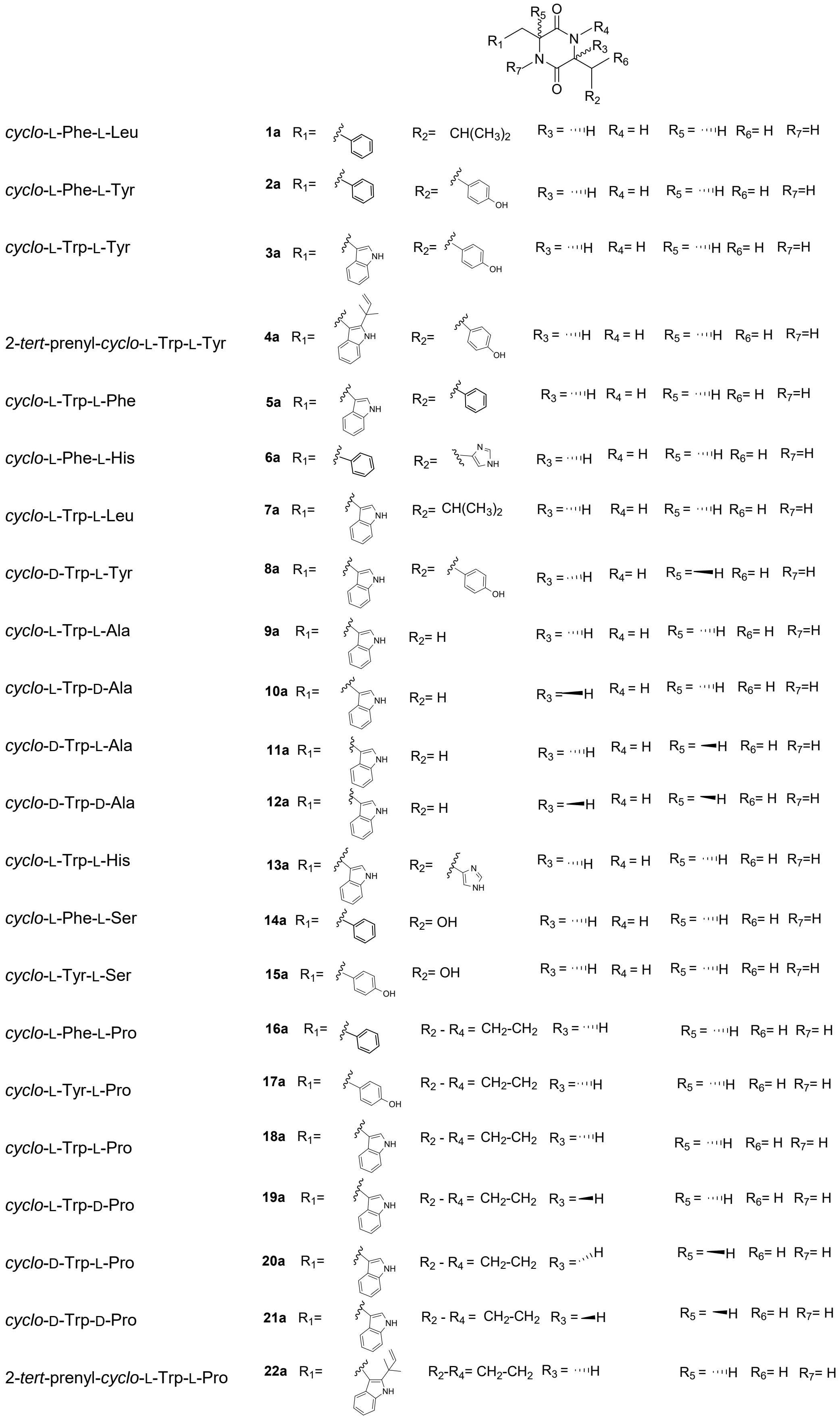

Figure S1: Overview of substrates used in this study (**1a − 22a**).

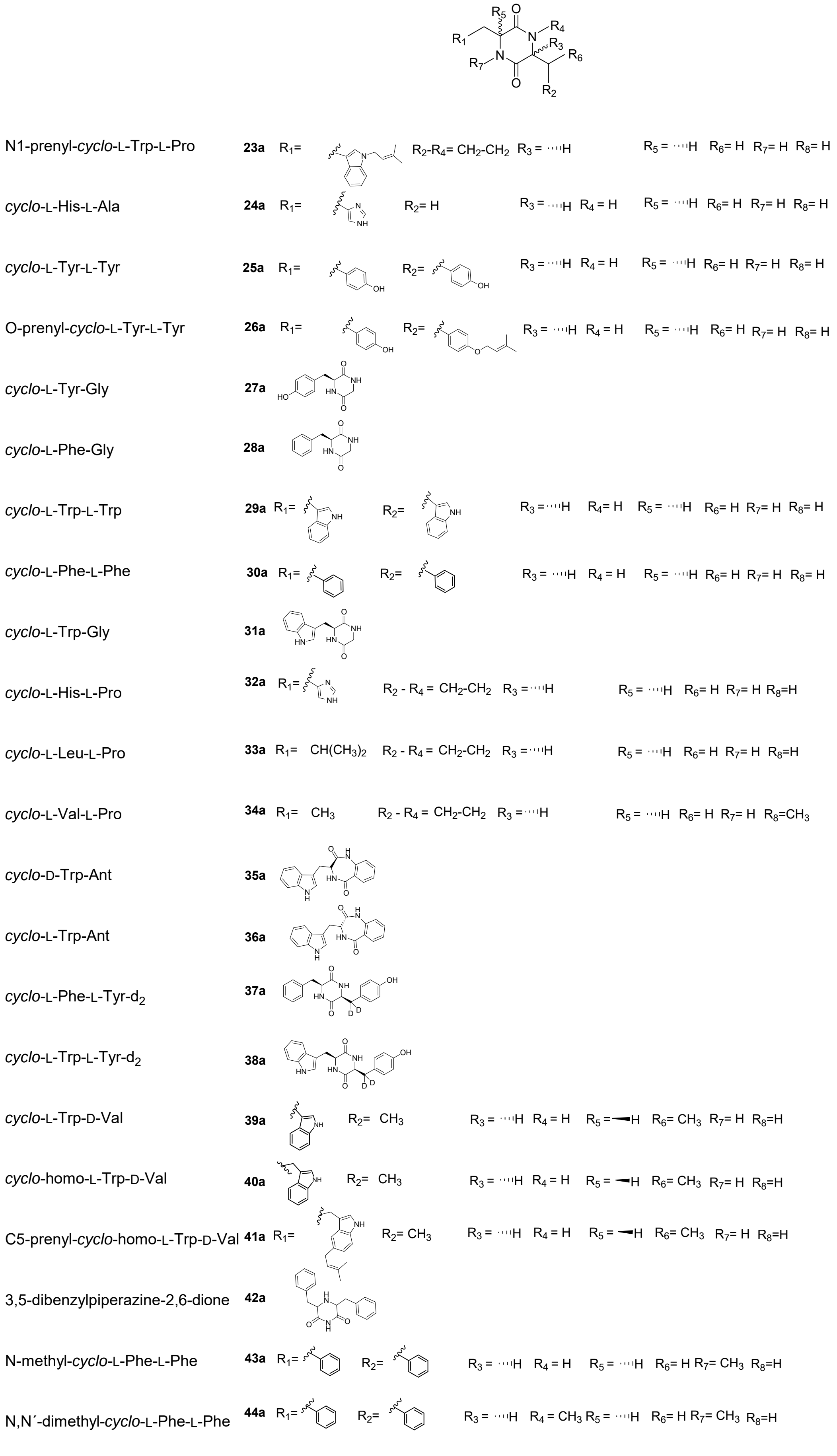

Figure S1: continued (**23a** – **44a**)

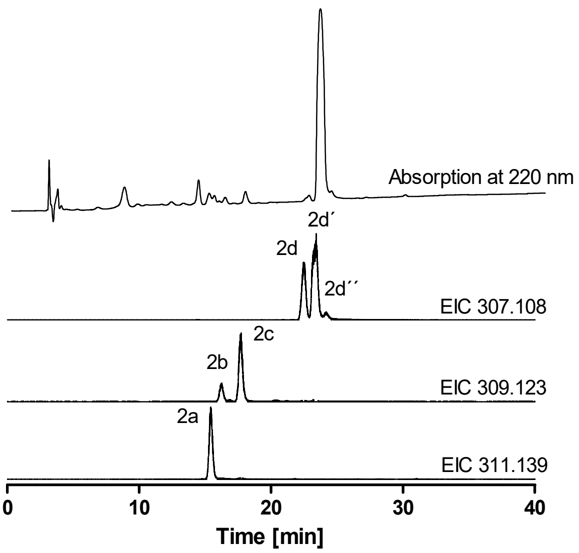

Figure S2: LC-MS chromatograms of the biotransformation of *cyclo*-L-Phe-L-Tyr (**2a**) by *E.coli* expressing CDO-Np after 16 h of incubation. The top chromatogram illustrates UV absorption and the three below refer to EICs for the substrate (**2a**), di- (**2b** and **2c**), and tetradehydrogenated products (**2d**) with a tolerance of ± 0.005.

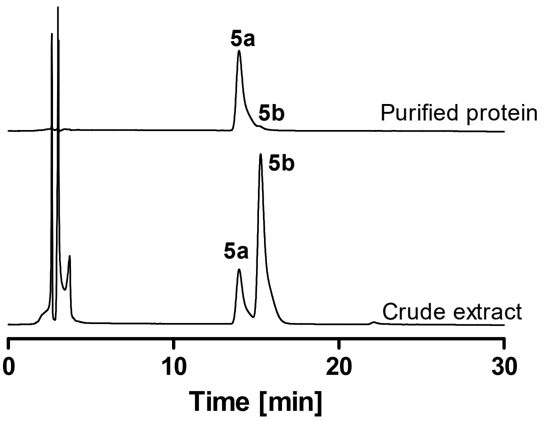

Figure S3: HPLC chromatograms of CDO-Np assays with *cyclo*-L-Trp-L-Phe (**5a**) before and after a Nickel-NTA agarose purification. The absorptions at 280 nm are illustrated.

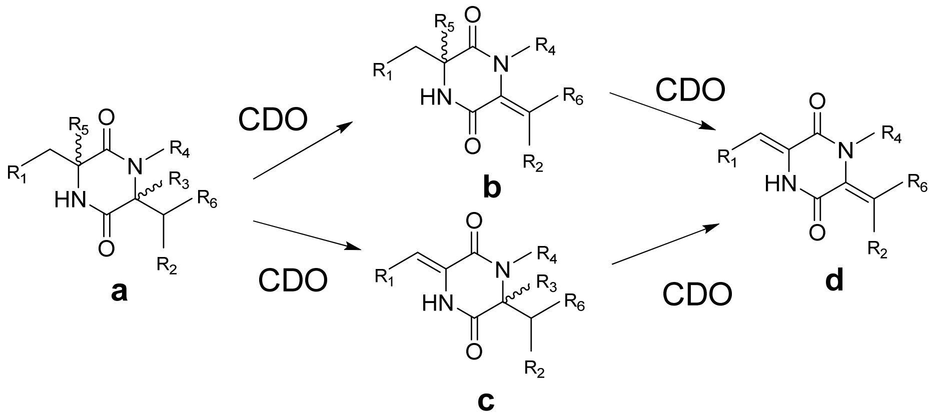

substrate	products	CDO-NP	AlbA/B	Ndas_1146/7
		product yields (%) after 2 h incubation with		
**14a**	**14c**: *cyclo*-ΔPhe-L-Ser	13.3 ± 0.6	2.9 ± 0.2	≤ 0.1
	**14d**: *cyclo*-ΔPhe-ΔSer	0.7 ± 0.4	0.3 ± 0.01	≤ 0.1
**15a**	**15c**: *cyclo*-ΔTyr-L-Ser	**24.6 ± 2.0**	≤ 0.1	≤ 0.1
**30a**	**30b**: *cyclo*-ΔPhe-L-Phe	≤ 0.1	≤ 0.1	≤ 0.1
	**30d**: *cyclo*-ΔPhe-ΔPhe	10.6 ± 0.5	0.3 ± 0.02	2.8 ± 0.7
**39a**	**39c**: *cyclo*-ΔTrp-D-Val	1.4 ± 0.2	≤ 0.1	≤ 0.1
**40a**	**40b**: *cyclo*-homo-L-Trp-ΔVal	2.0 ± 0.1	≤ 0.1	≤ 0.1
	**40c**: *cyclo*-homo-ΔTrp-D-Val	4.5 ± 0.1	4.3 ± 0.1	≤ 0.1

Figure S4: Dehydrogenation reactions of CDPs catalysed by the three CDOs with a conversion yield smaller than 30 %. Product yields of more than 20 % are highlighted in bold. Product yields were calculated by using the area under the curves of the respective extracted ion chromatogram and ± indicated the main value of two independent experiments. See Figure S1 for detailed structure information.

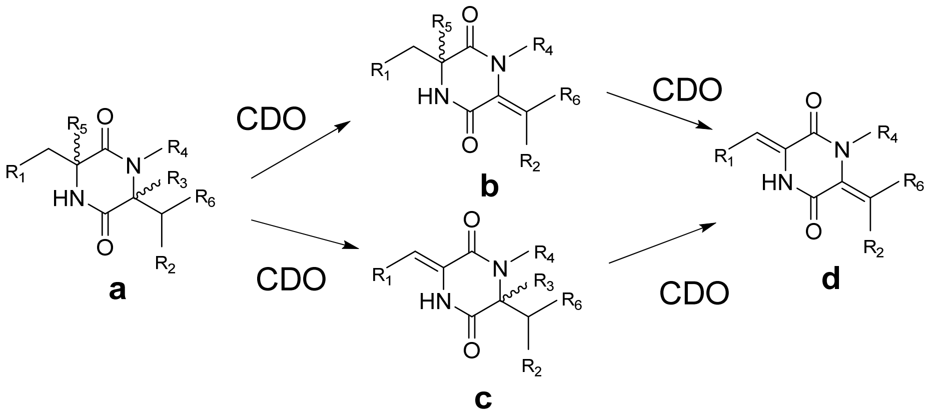

substrate	products	CDO-NP	AlbA/B	Ndas_1146/7
		product yields (%) after 2 h incubation by		
**10a**	**10b**: *cyclo*-L-Trp-ΔAla	17.3 ± 2.2	0.1 ± 0.01	≤ 0.1.
	**10c**: *cyclo*-ΔTrp-L-Ala	3.4 ± 0.2	1.4 ± 1.3	0.3 ± 0.02
**11a**	**11b**: *cyclo*-D-Trp-ΔAla	14.0 ± 0.5	0.4 ± 0.3	≤ 0.1
	**11c**: *cyclo*-ΔTrp-L-Ala	2.7 ± 0.02	1.4 ± 1,2	0.3 ± 0.02
**12a**	**12b**: *cyclo*-D-Trp-ΔAla	4.6 ± 0.02	0.2 ± 0.01	0.1 ±0.02
	**12d**: *cyclo*-ΔTrp-ΔAla	6.4 ± 0.28	≤ 0.1	≤ 0.1
**18a**	**18b**: *cyclo*-L-Trp-ΔPro	**20.8 ± 0.6**	3.6 ± 0.04	1.0 ± 0.2
	**18d**: *cyclo*-ΔTrp-ΔPro	2.8 ± 0.1	≤ 0.1	≤ 0.1
**19a**	**19b**: *cyclo*-L-Trp-ΔPro	0.1 ± 0.01	≤ 0.1	≤ 0.1
	**19c**: *cyclo*-ΔTrp-D-Pro	0.1 ± 0.05	≤ 0.1	0.4 ± 0.03
**20a**	**20b**: *cyclo*-D-Trp-ΔPro	0.7 ± 0.01	≤ 0.1	≤ 0.1
	**20c**: *cyclo*-ΔTrp-L-Pro	0.1 ± 0.02	0.2 ±0.02	0.6 ±0.04
**21a**	**21b**: *cyclo*-D-Trp-ΔPro	1.4 ± 0.06	≤ 0.1	≤ 0.1
	**21c**: *cyclo*-ΔTrp-D-Pro	≤ 0.1	0.2 ± 0.03	0.5 ± 0.06
	**21d**: *cyclo*-ΔTrp-ΔPro	0.2 ± 0.01	≤ 0.1	≤ 0.1

Figure S5: Dehydrogenation reactions of CDPs with different D/L configurations catalysed by the three CDOs. Product yields of more than 20% are highlighted in bold. Product yields were calculated by using the area under the curves of the respective extracted ion chromatogram and ± indicated the main value of two independent experiments. See Figure S1 for detailed structure information.

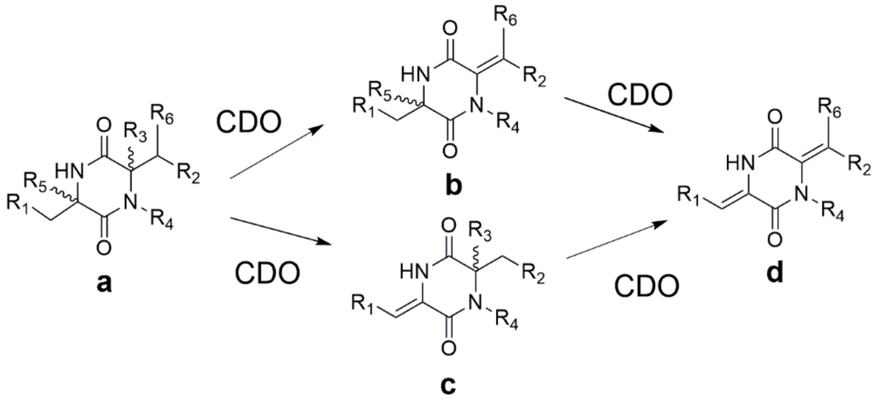

substrates	products	product yields (%) after 2 h incubation by CDO-NP	AlbA/B	Ndas_1146/7
**4a**	**4b**:2-*tert*-prenyl-*cyclo*-L-Trp-ΔTyr	23.7 ± 0.4	≤ 0.1	3.3 ± 0.1
	**4c**:2-*tert*-prenyl-*cyclo*-ΔTrp-L-Tyr	5.6 ± 0.7	4.3 ± 0.02	5.2 ± 0.7
	**4d**:2-*tert*-prenyl-*cyclo*-ΔTrp-ΔTyr	0.7 ± 0.04	0,2 ± 0.03	0.5 ± 0.1
**22a**	**22b**:2-*tert*-prenyl-*cyclo*-L-Trp-ΔPro	≤ 0.1	≤ 0.1	≤ 0.1
**23a**	**23b**:N1-prenyl-*cyclo*-L-Trp-ΔPro	5.8 ±0.4	5.4 ± 0.2	6.2 ± 0.1
**26a**	**26b**:O-prenyl-*cyclo*-L-Tyr-ΔTyr	63.2 ± 0.3	1.1 ± 0.3	14.8 ± 0.5
	**26c**:O-prenyl-*cyclo*-ΔTyr-L-Tyr	≤ 0.1	1.6 ± 0.1	2.0 ± 0.1
	**26d**:O-prenyl-*cyclo*-ΔTyr-ΔTyr	18.0 ± 1.5	≤ 0,1	9.4 ± 0.7
**41a**	**41b**:5-prenyl-*cyclo*-homo-L-Trp-ΔVal	0.9 ± 0.02	1.08 ± 0.1	1.45 ± 0.02
	**41c**:5-prenyl-*cyclo*-homo-ΔTrp-D-Val	3.4 ± 1.2	3.6 ± 0.2	2.2 ± 0.2
	**41d**:5-prenyl-*cyclo*-homo-ΔTrp-ΔVal	0.8 ± 0.03	0.9 ± 0.01	0.7 ± 0.04

Figure S6: Dehydrogenation reactions of prenylated CDPs catalysed by the three CDOs. Product yields of more than 20% are highlighted in bold. Product yields were calculated by using the area under the curves of the respective extracted ion chromatogram and ± indicated the main value of two independent experiments. See Figure S1 for detailed structure information.

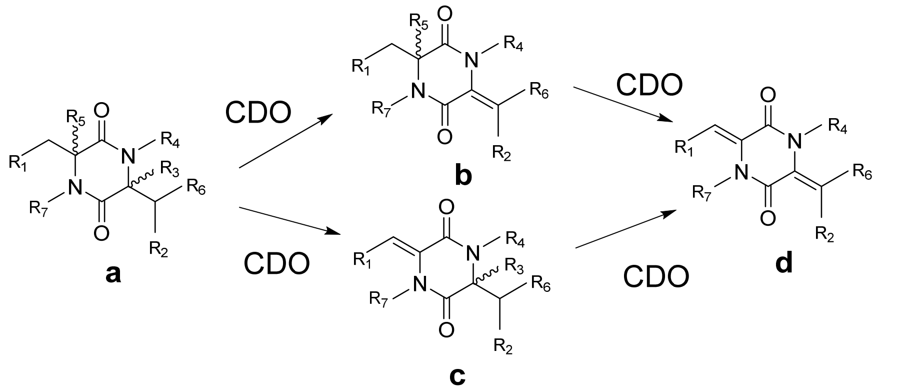

substrate	products	product yields (%) after 2 h incubation with		
		CDO-NP	AlbA/B	Ndas_1146/7
**35a**	**35b**: cyclo-ΔTrp-Ant	0.2 ± 0.01	0.15 ± 0.02	0.19 ± 0.01
**36a**	**36b**: cyclo-ΔTrp-Ant	< 0,1	< 0,1	< 0,1
**42a**	**42a:** 3,5-dibenzylpiperazine-2,6-dione	< 0,1	< 0,1	< 0,1
**43a**	**43b**: N-methyl-cyclo-L-Phe-ΔPhe	22.0 ± 0.5	0.3 ± 0.02	1.4 ± 0.1
	**43d**: N-methyl-cyclo-ΔPhe-ΔPhe	0.2 ± 0.01	< 0,1	< 0,1
**44a**	**44**: N,N´-dimethyl-cyclo-L-Phe-L-Phe	< 0,1	< 0,1	< 0,1

Figure S7: Dehydrogenation reactions of CDPs with a modified diketopiperazine ring catalysed by the three CDOs. Product yields of more than 20% are highlighted in bold. Product yields were calculated by using the area under the curves of the respective extracted ion chromatogram and ± indicated the main value of two independent experiments. See Figure S1 for detailed structure information.

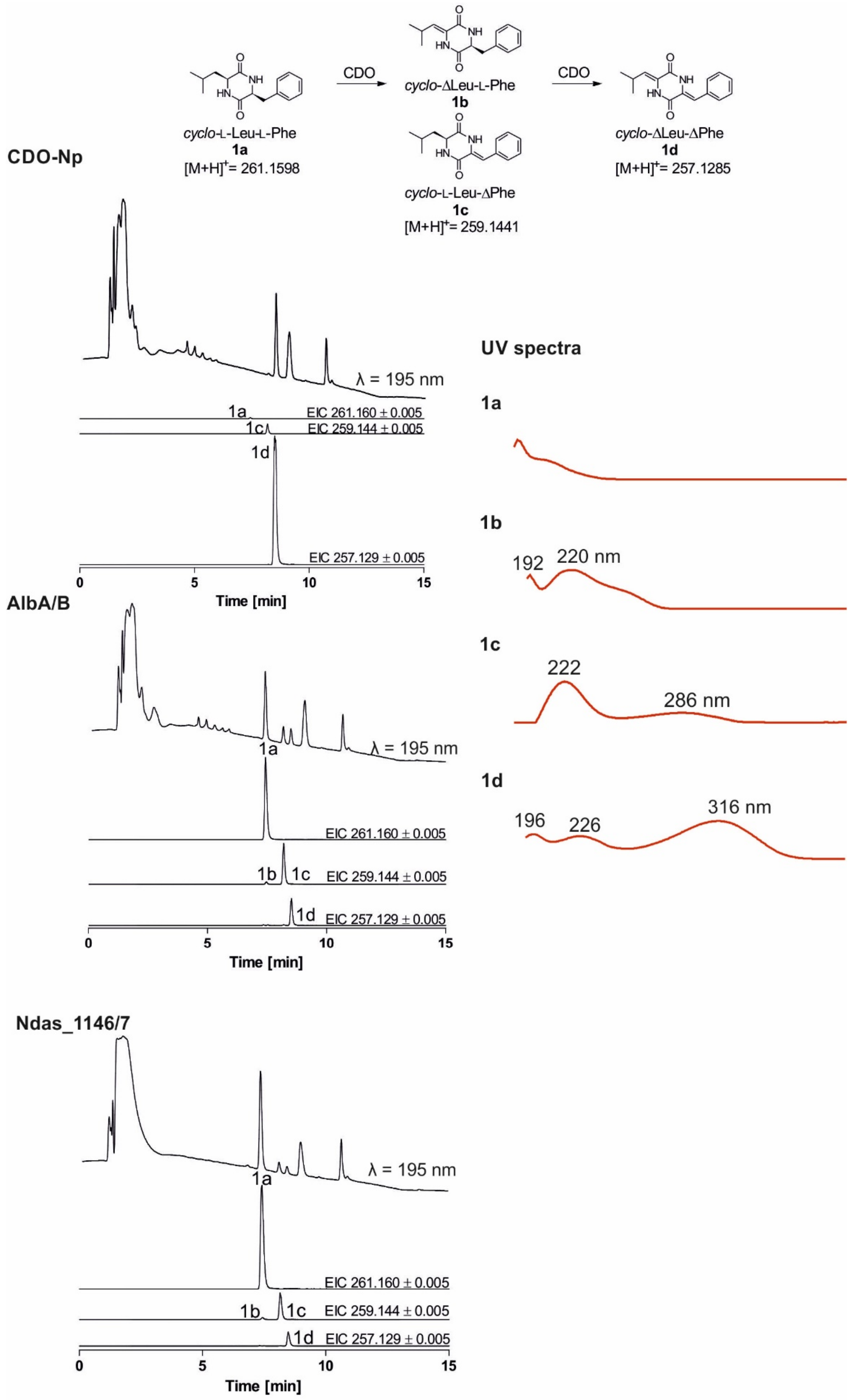

Figure S8: Overview of the conversion of **1a** to its dehydrogenated products by the 3 CDO-containing cell free extracts after 2h of incubation. The respective top chromatogram illustrates the UV absorption and the EICs for the substrate (**1a**), di- (**1b** and **1c**), and tetradehydrogenated products (**1d**) are displayed below with a tolerance of ± 0.005. UV spectra of the substrate and products are displayed on the right side.

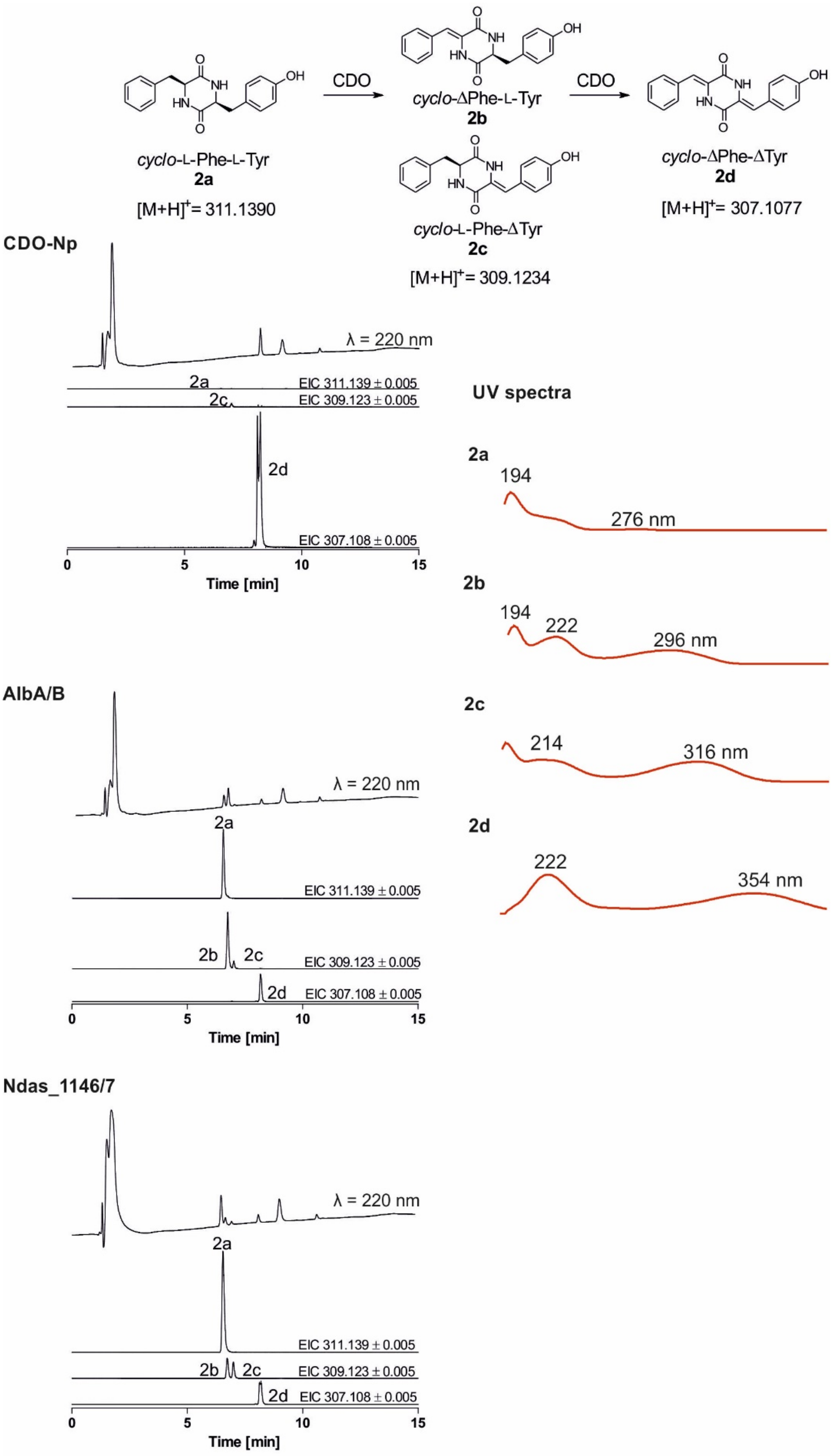

Figure S9: Overview of the conversion of **2a** to its dehydrogenated products by the 3 CDO-containing cell free extracts after 2h of incubation. The respective top chromatogram illustrates the UV absorption and the EICs for the substrate (**2a**), di- (**2b** and **2c**), and tetradehydrogenated products (**2d**) are displayed below with a tolerance of ± 0.005. UV spectra of the substrate and products are placed on the right side.

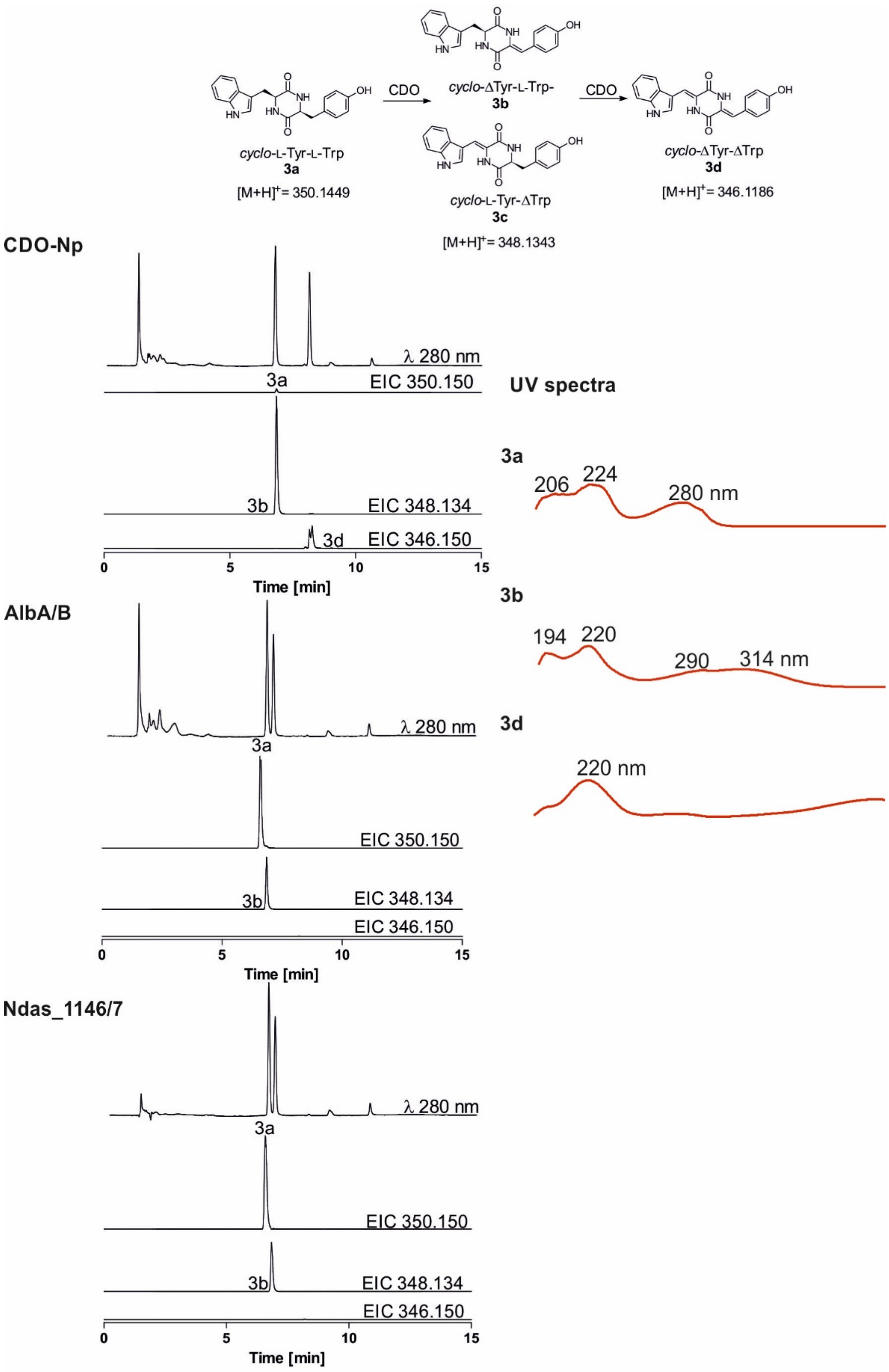

Figure S10: Overview of the conversion of **3a** to its dehydrogenated products by the 3 CDO-containing cell free extracts after 2h of incubation. The respective top chromatogram illustrates the UV absorption and the EICs for the substrate (**3a**), di- (**3b** and **3c**), and tetradehydrogenated products (**3d**) are displayed below with a tolerance of ± 0.005. UV spectra of the substrate and products are placed on the right side.

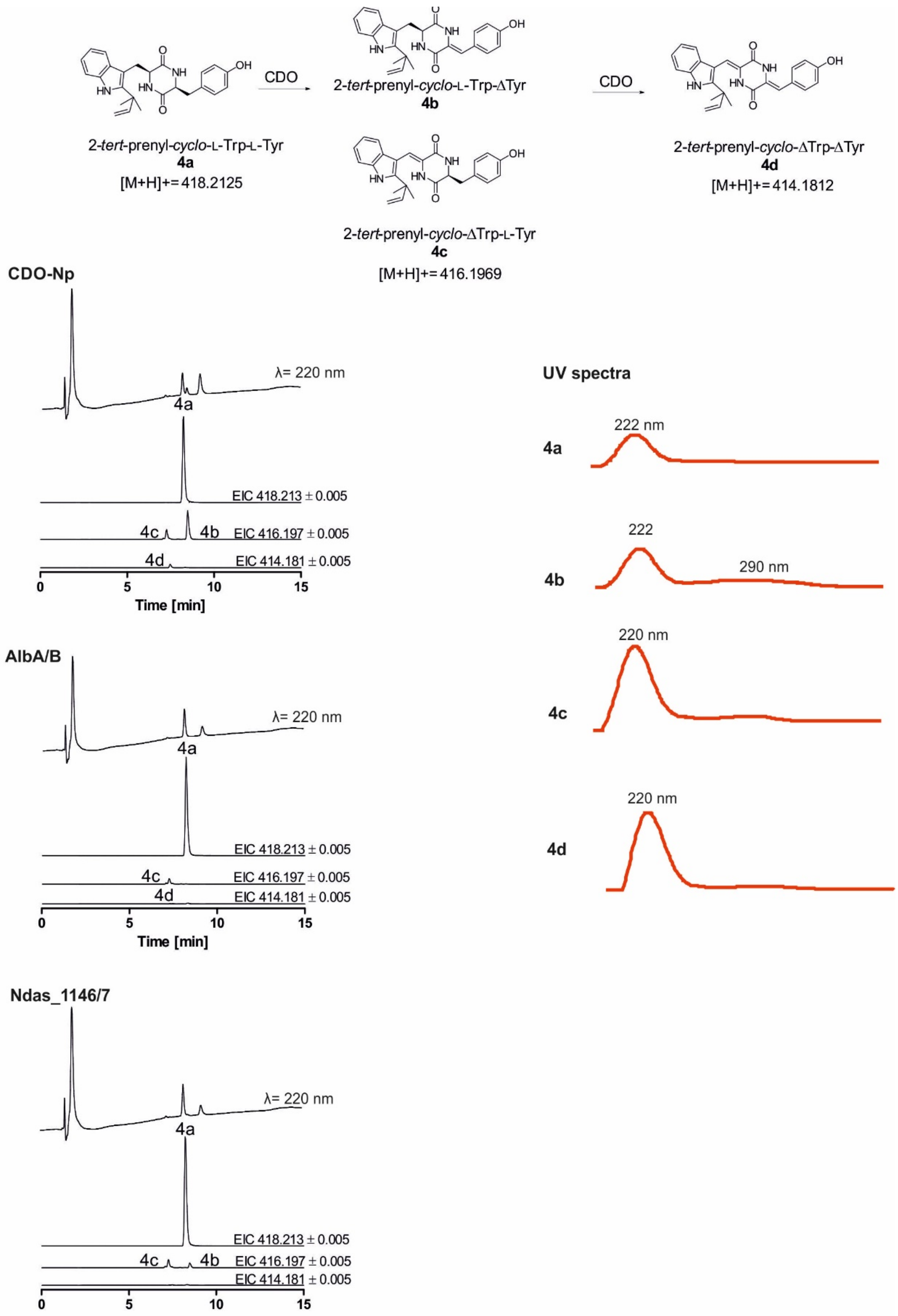

Figure S11: Overview of the conversion of **4a** to its dehydrogenated products by the 3 CDO-containing cell free extracts after 2h of incubation. The respective top chromatogram illustrates the UV absorption and the EICs for the substrate (**4a**), di- (**4b** and **4c**), and tetradehydrogenated products (**4d**) are displayed below with a tolerance of ± 0.005. UV spectra of the substrate and products are placed on the right side.

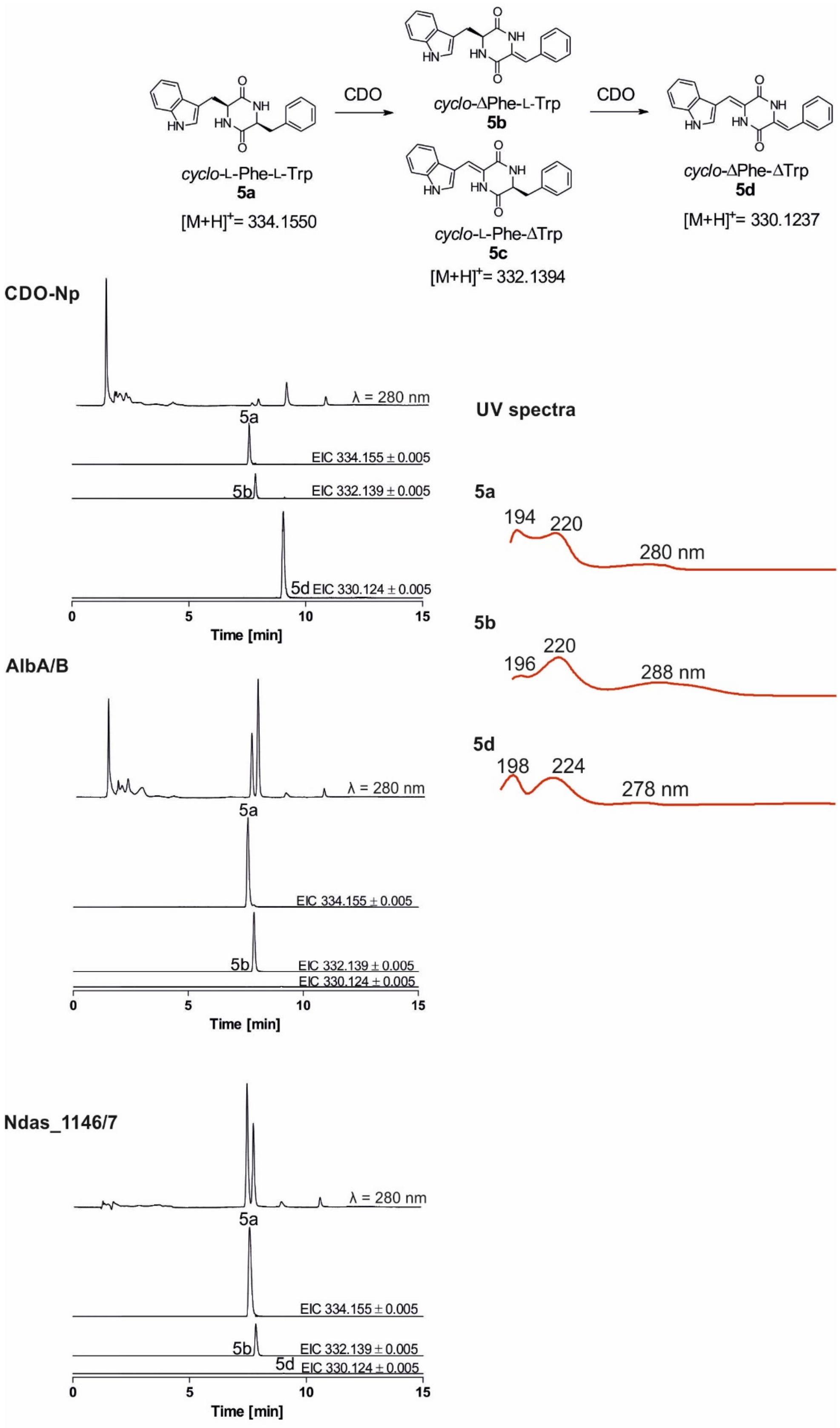

Figure S12: Overview of the conversion of **5a** to its dehydrogenated products by the 3 CDO-containing cell free extracts after 2h of incubation. The respective top chromatogram illustrates the UV absorption and the EICs for the substrate (**5a**), di- (**5b** and **5c**), and tetradehydrogenated products (**5d**) are displayed below with a tolerance of ± 0.005. UV spectra of the substrate and products are placed on the right side.

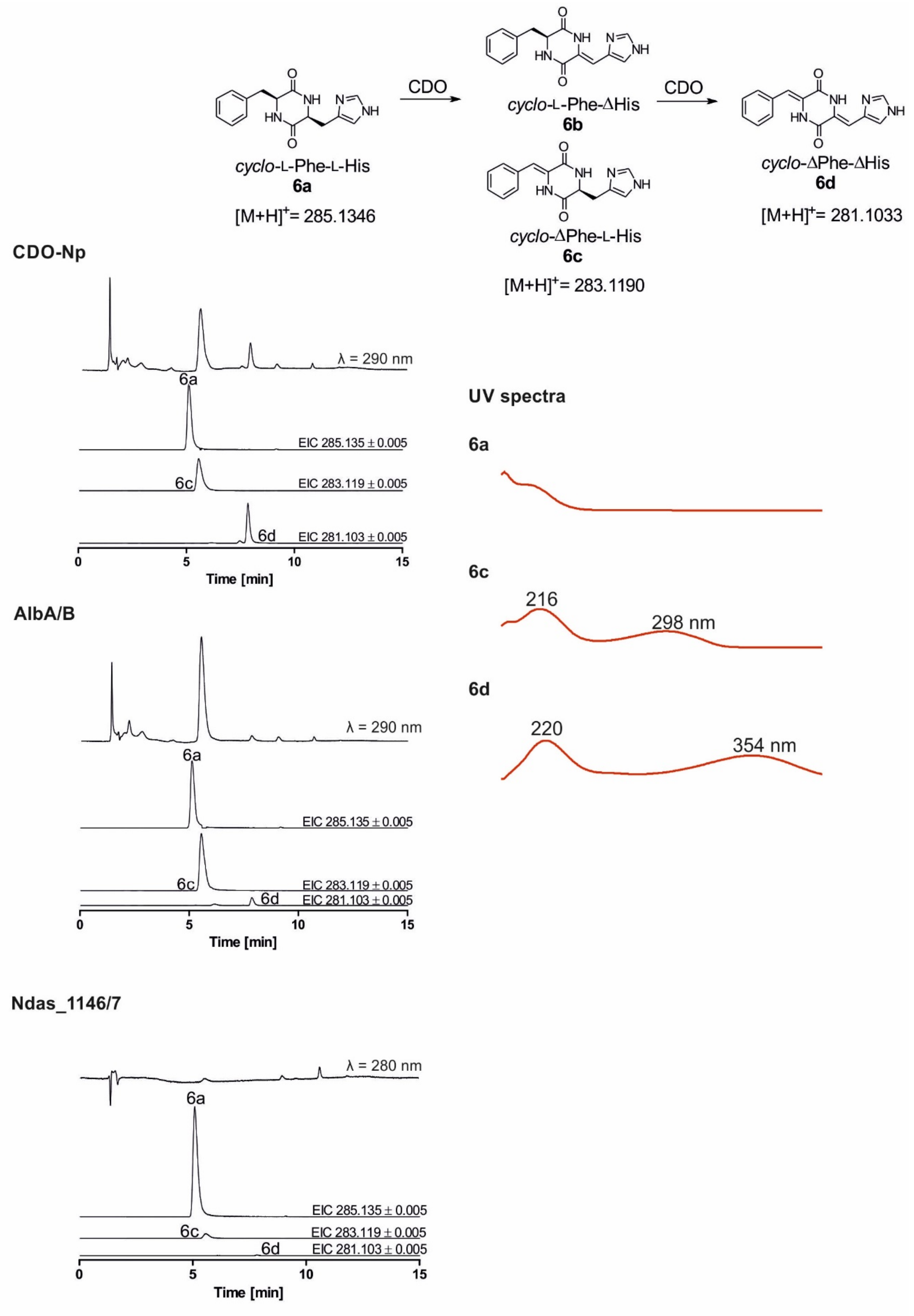

Figure S13: Overview of the conversion of **6a** to its dehydrogenated products by the 3 CDO-containing cell free extracts after 2h of incubation. The respective top chromatogram illustrates the UV absorption and the EICs for the substrate (**6a**), the di- (**6b** and **6c**), and tetradehydrogenated products (**6d**) are displayed below with a tolerance of ± 0.005. UV spectra of the substrate and products are placed on the right side.

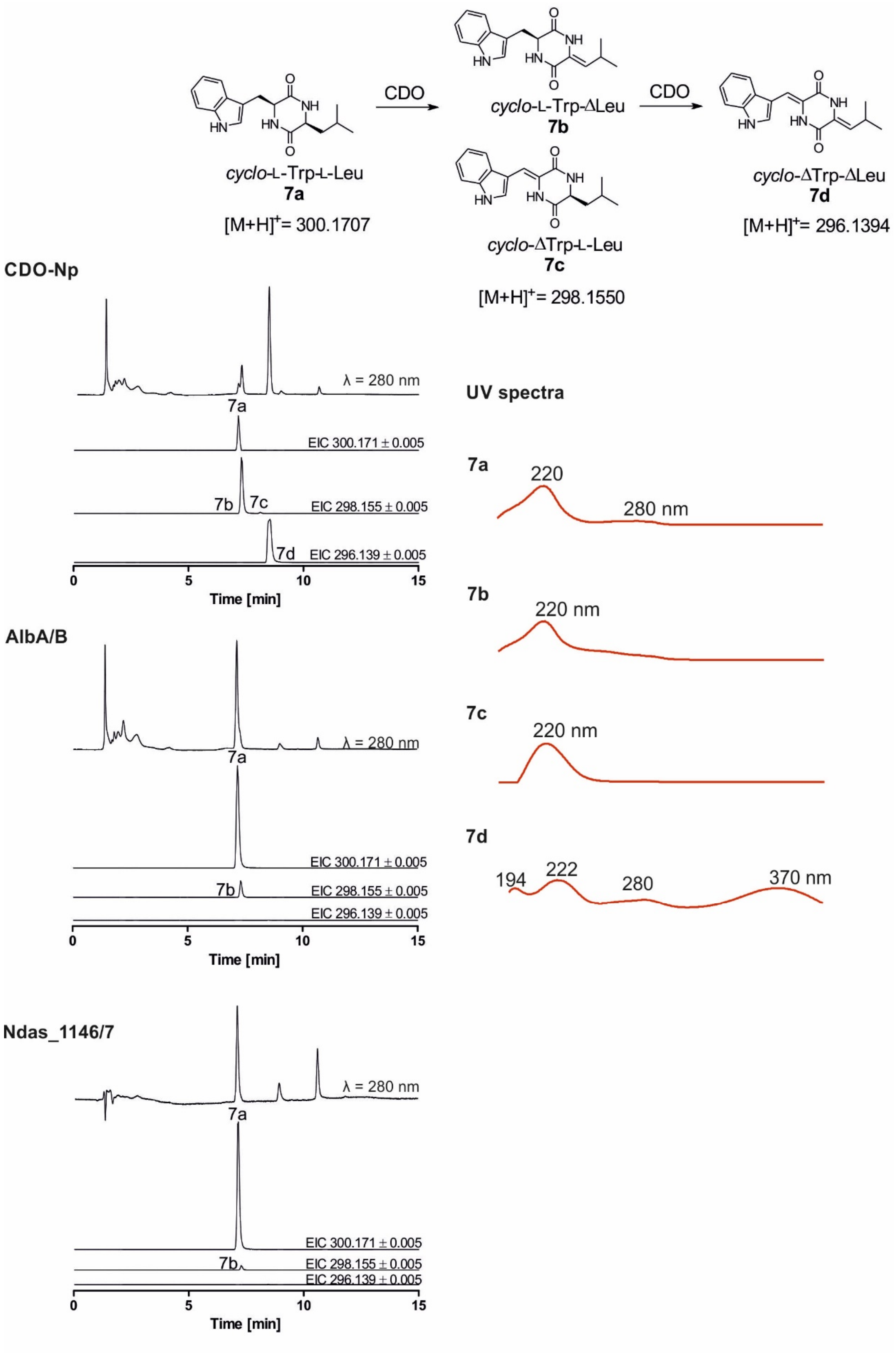

Figure S14: Overview of the conversion of **7a** to its dehydrogenated products by the 3 CDO-containing cell free extracts after 2h of incubation. The respective top chromatogram illustrates the UV absorption and EICs for the substrate (**7a**), the di- (**7b** and **7c**), and tetradehydrogenated products (**7d**) are displayed below with a tolerance of ± 0.005. UV spectra of the substrate and products are placed on the right side.

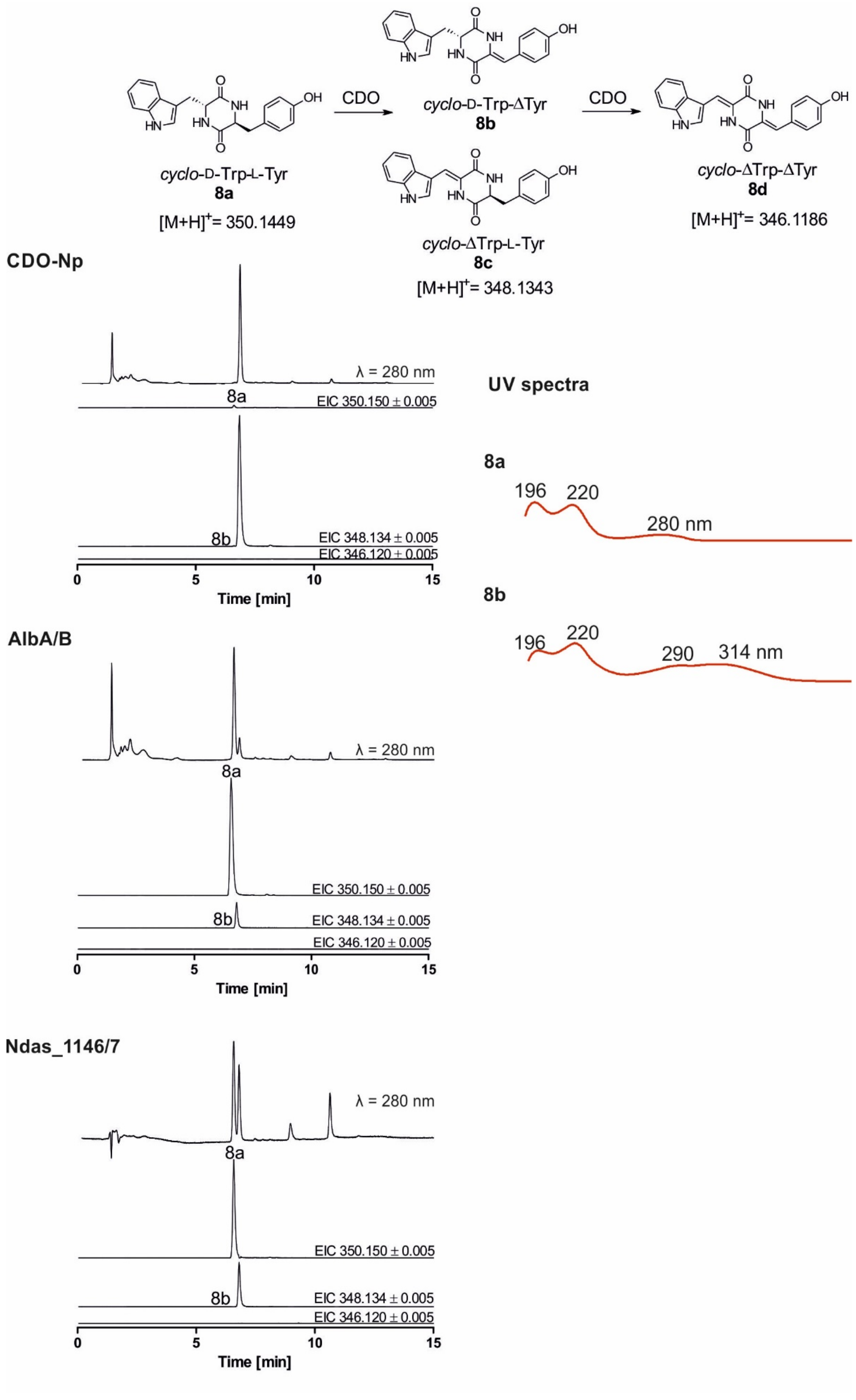

Figure S15: Overview of the conversion of **8a** to its dehydrogenated products by the 3 CDO-containing cell free extracts after 2h of incubation. The respective top chromatogram illustrates the UV absorption and the EICs for the substrate (**8a**), di- (**8b** and **8c**), and tetradehydrogenated products (**8d**) are displayed below with a tolerance of ± 0.005. UV spectra of the substrate and products are placed on the right side.

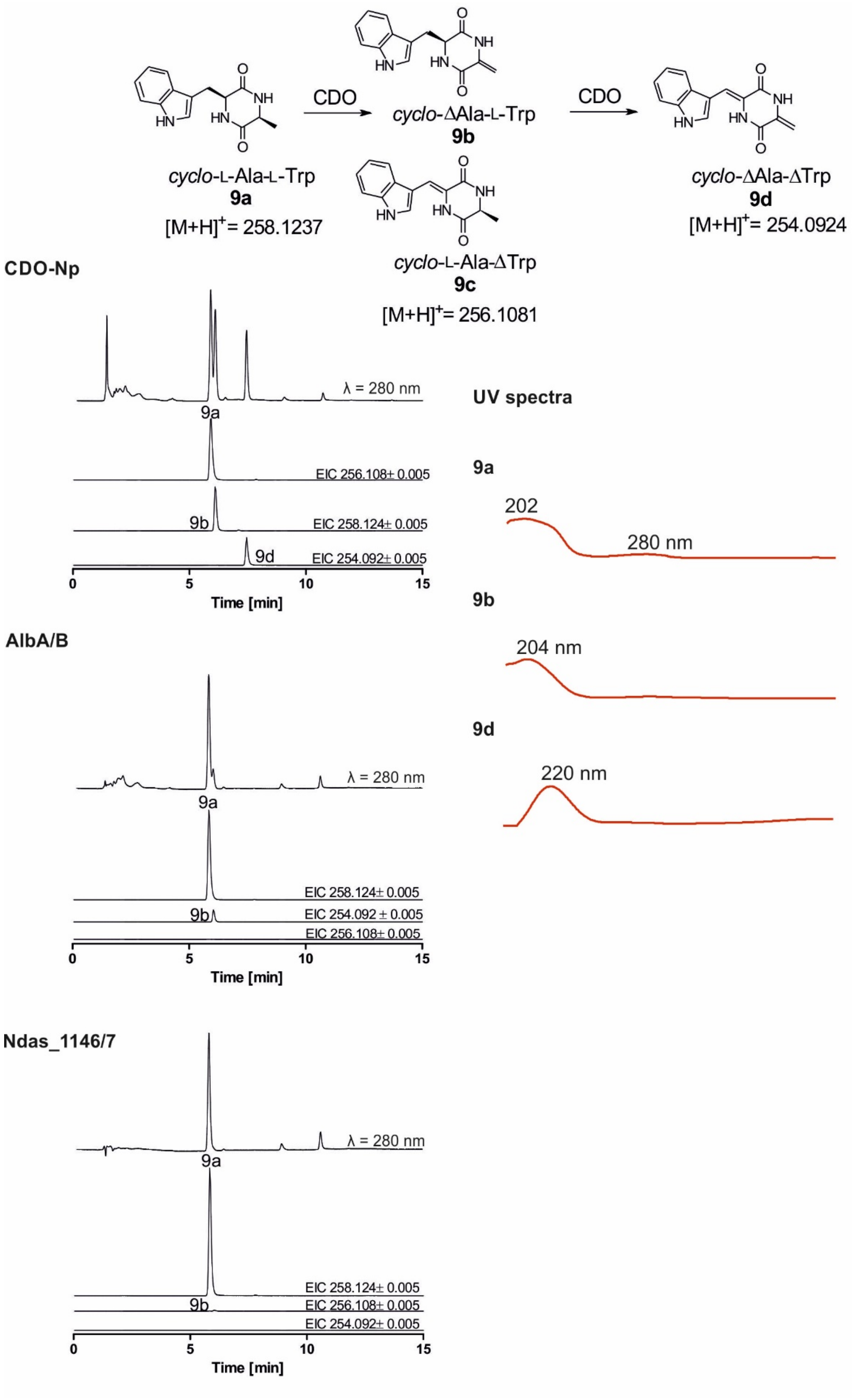

Figure S16: Overview of the conversion of **9a** to its dehydrogenated products by the 3 CDO-containing cell free extracts after 2h of incubation. The respective top chromatogram illustrates the UV absorption and the EICs for the substrate (**9a**), di- (**9b** and **9c**), and tetradehydrogenated products (**9d**) are displayed below with a tolerance of ± 0.005. UV spectra of the substrate and products are placed on the right side.

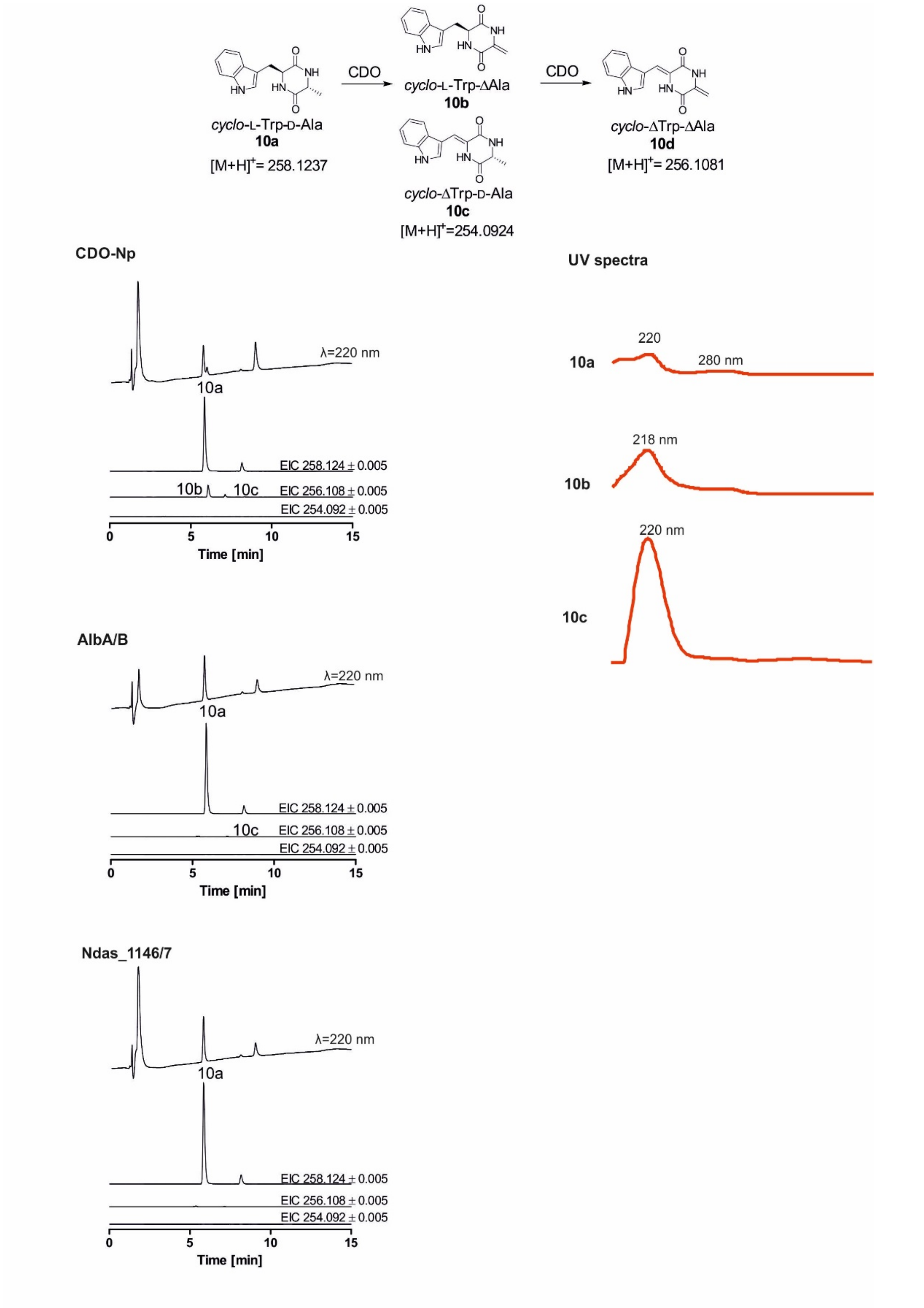

Figure S17: Overview of the conversion of **10a** to its dehydrogenated products by the 3 CDO-containing cell free extracts after 2h of incubation. The respective top chromatogram illustrates the UV absorption and the EICs for the substrate (**10a**), di- (**10b** and **10c**), and tetradehydrogenated products (**10d**) are displayed below with a tolerance of ± 0.005. UV spectra of the substrate and products are placed on the right side.

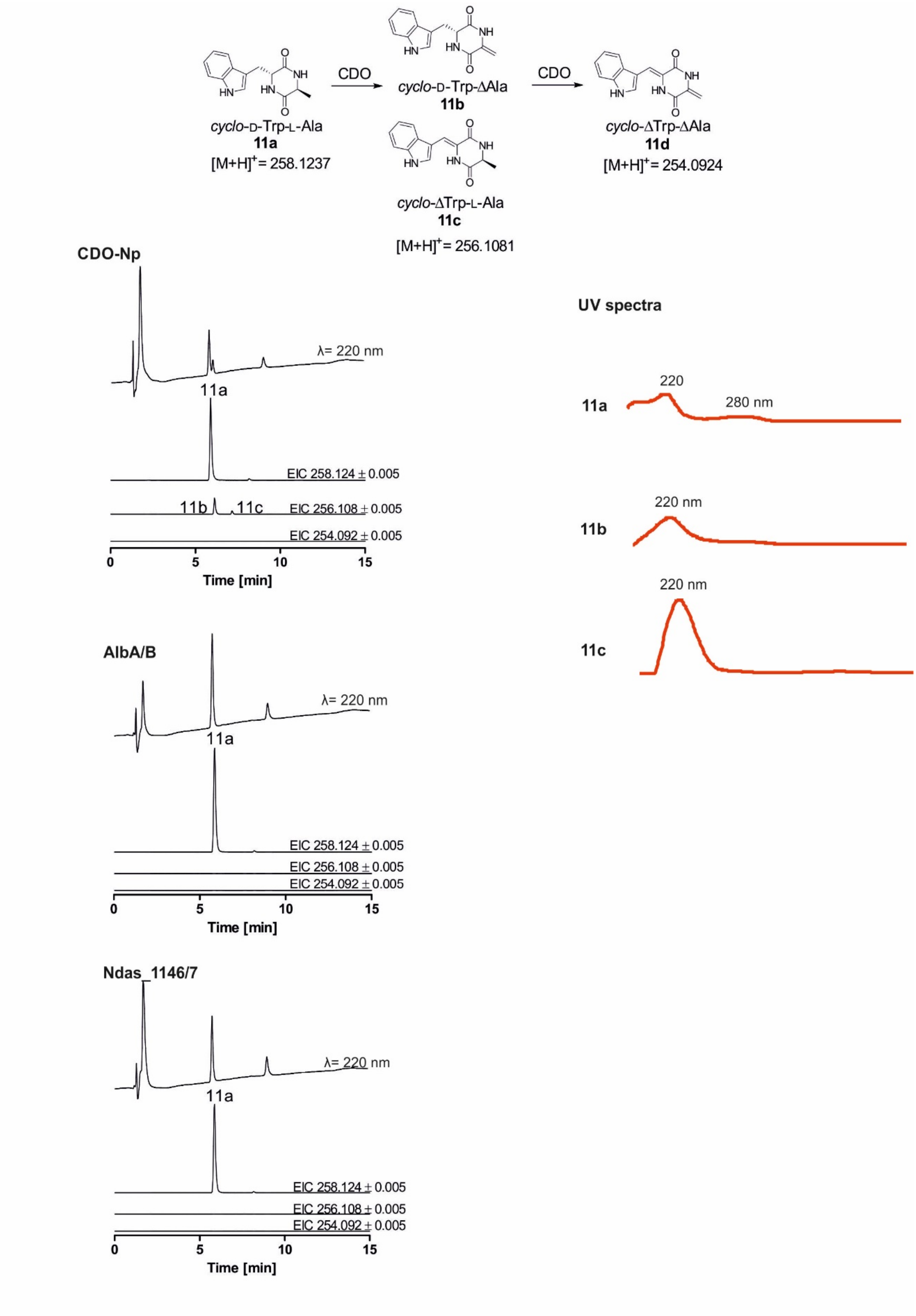

Figure S18: Overview of the conversion of **11a** to its dehydrogenated products by the 3 CDO-containing cell free extracts after 2h of incubation. The respective top chromatogram illustrates the UV absorption and the EICs for the substrate (**11a**), di- (**11b** and **11c**), and tetradehydrogenated products (**11d**) are displayed below with a tolerance of ± 0.005. UV spectra of the substrate and products are placed on the right side.

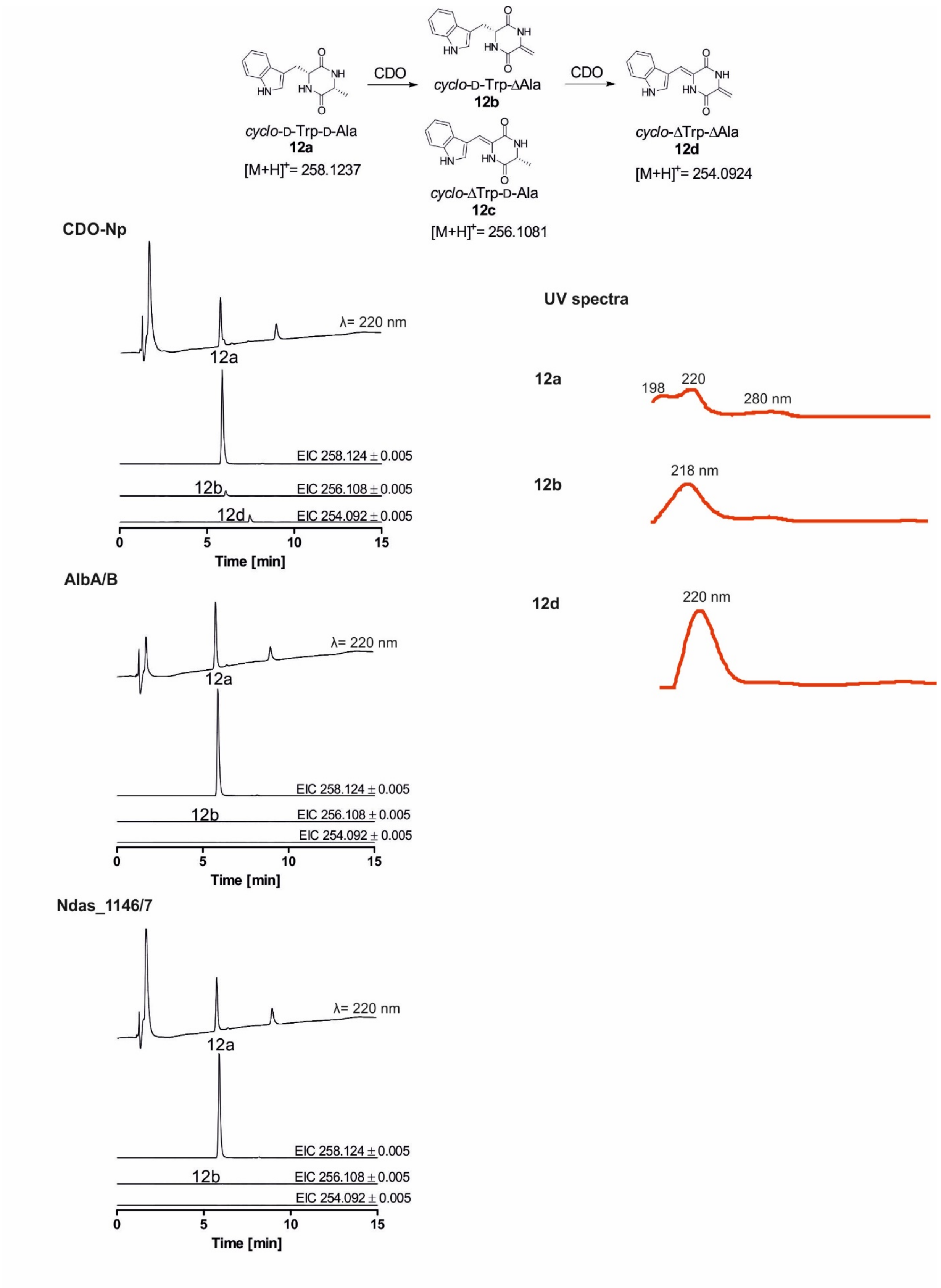

Figure S19: Overview of the conversion of **12a** to its dehydrogenated products by the 3 CDO-containing cell free extracts after 2h of incubation. The respective top chromatogram illustrates the UV absorption and the EICs for the substrate (**12a**), di- (**12b** and **12c**), and tetradehydrogenated products (**12d**) are displayed below with a tolerance of ± 0.005. UV spectra of the substrate and products are placed on the right side.

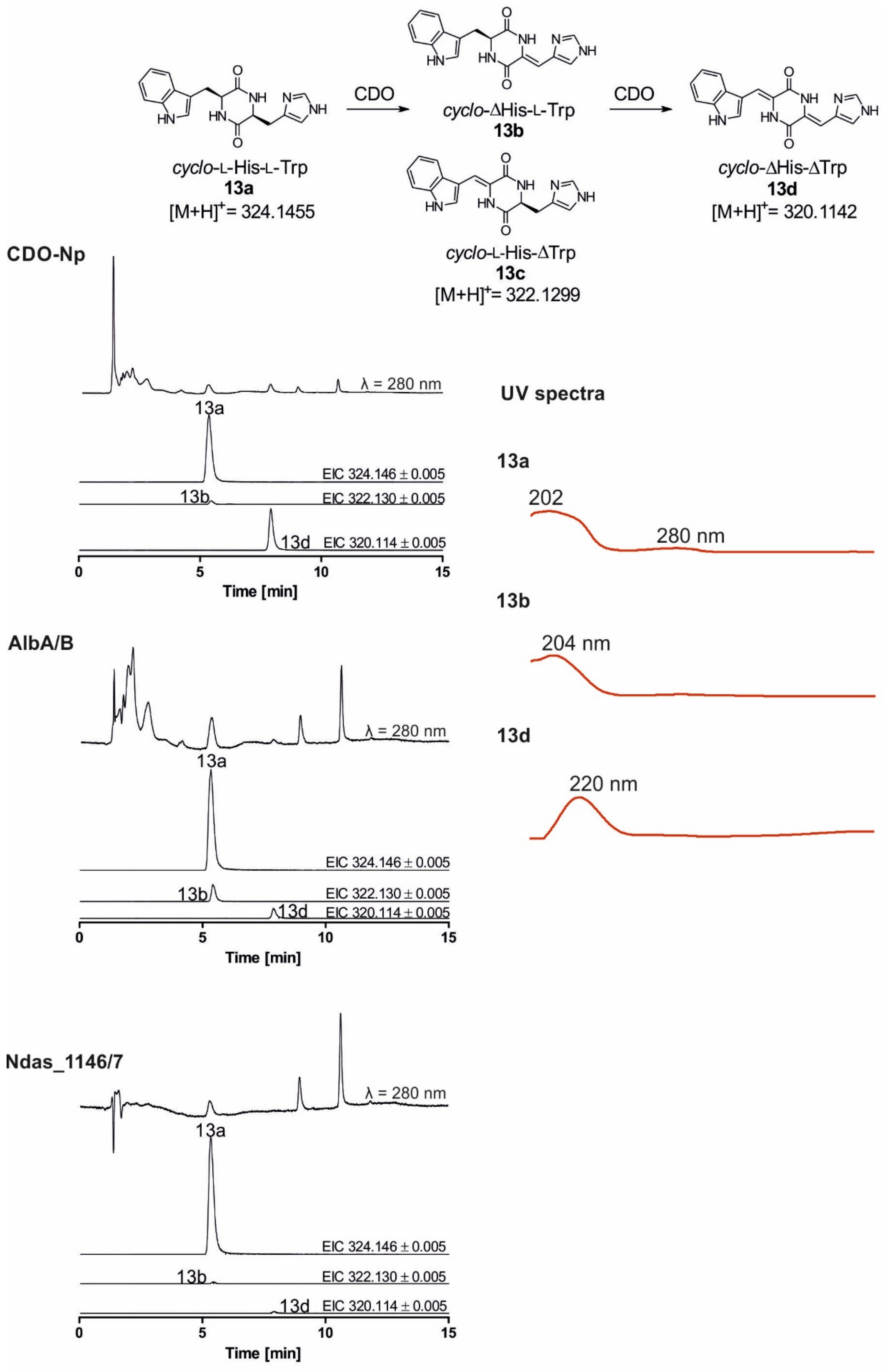

Figure S20: Overview of the conversion of **13a** to its dehydrogenated products by the 3 CDO-containing cell free extracts after 2h of incubation. The respective top chromatogram illustrates the UV absorption and the EICs for the substrate (**13a**), di- (**13b** and **13c**), and tetradehydrogenated products (**13d**) are displayed below with a tolerance of ± 0.005. UV spectra of the substrate and products are placed on the right side.

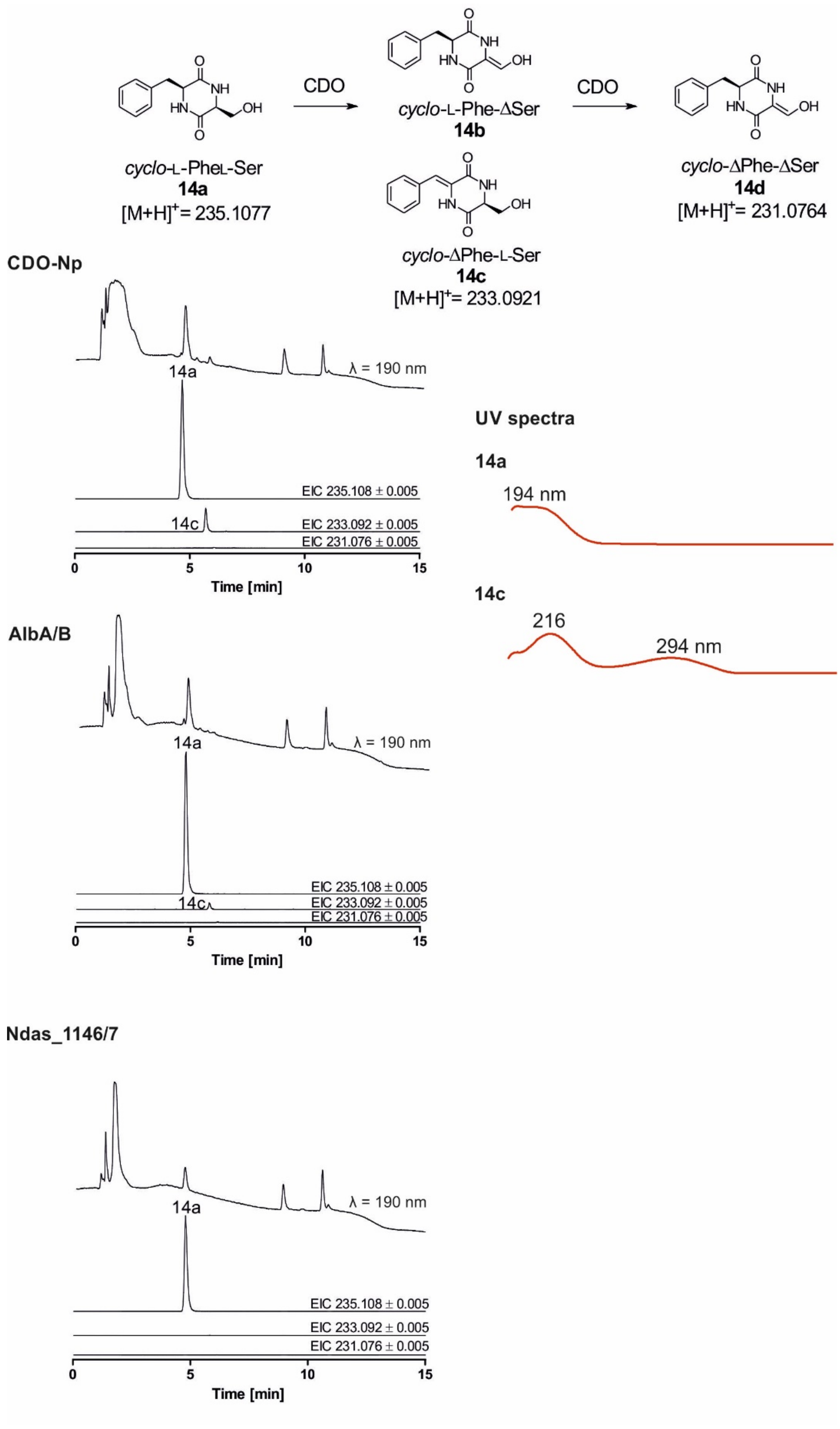

Figure S21: Overview of the conversion of **14a** to its dehydrogenated products by the 3 CDO-containing cell free extracts after 2h of incubation. The respective top chromatogram illustrates the UV absorption and the EICs for the substrate (**14a**), di- (**14b** and **14c**), and tetradehydrogenated products (**14d**) are displayed below with a tolerance of ± 0.005. UV spectra of the substrate and products are placed on the right side.

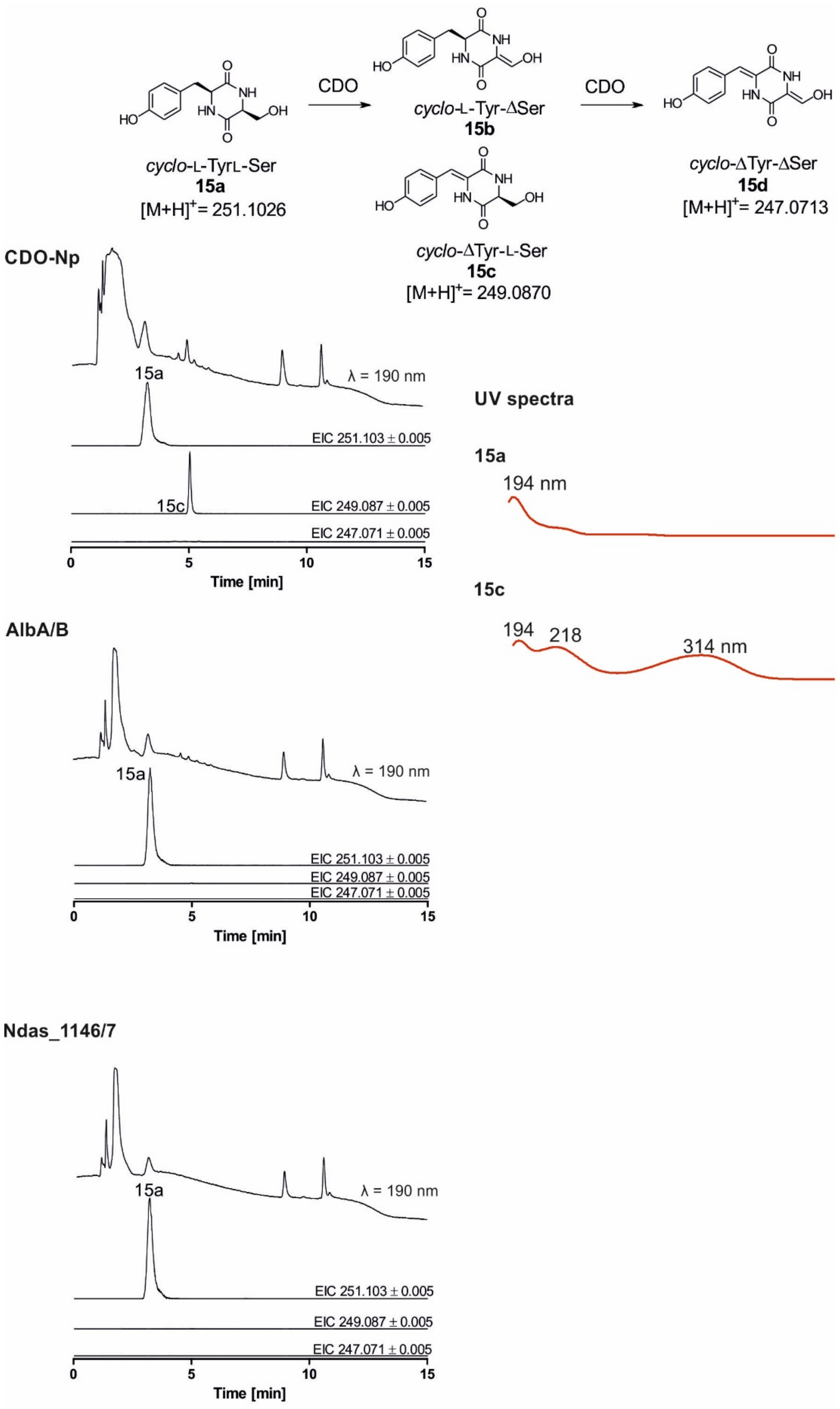

Figure S22: Overview of the conversion of **15a** to its dehydrogenated products by the 3 CDO-containing cell free extracts after 2h of incubation. The respective top chromatogram illustrates the UV absorption and the EICs for the substrate (**15a**), di- (**15b** and **15c**), and tetradehydrogenated products (**15d**) are displayed below with a tolerance of ± 0.005. UV spectra of the substrate and products are placed on the right side.

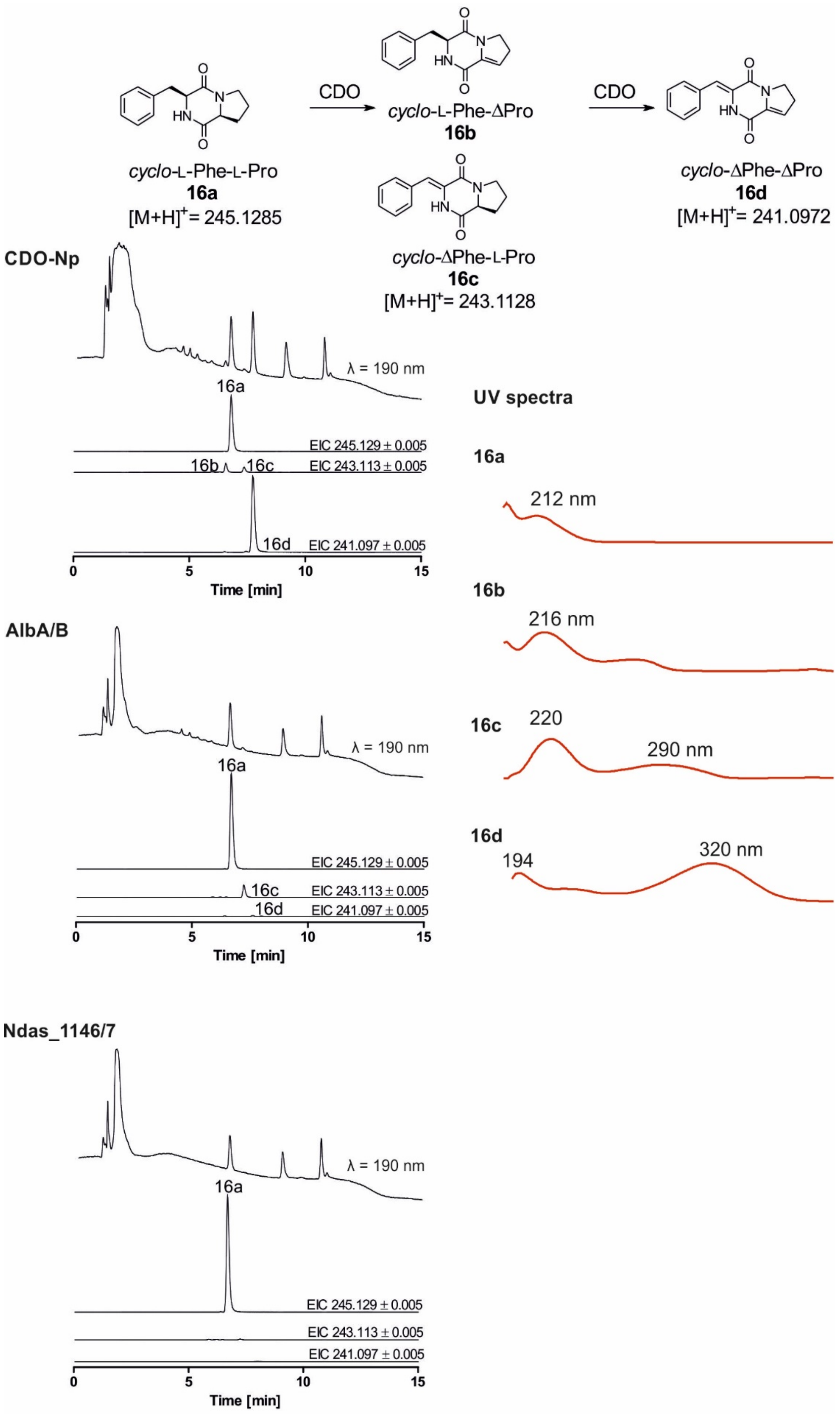

Figure S23: Overview of the conversion of **16a** to its dehydrogenated products by the 3 CDO-containing cell free extracts after 2h of incubation. The respective top chromatogram illustrates the UV absorption and the EICs for the substrate (**16a**), di- (**16b** and **16c**), and tetradehydrogenated products (**16d**) are displayed below with a tolerance of ± 0.005. UV spectra of the substrate and products are placed on the right side.

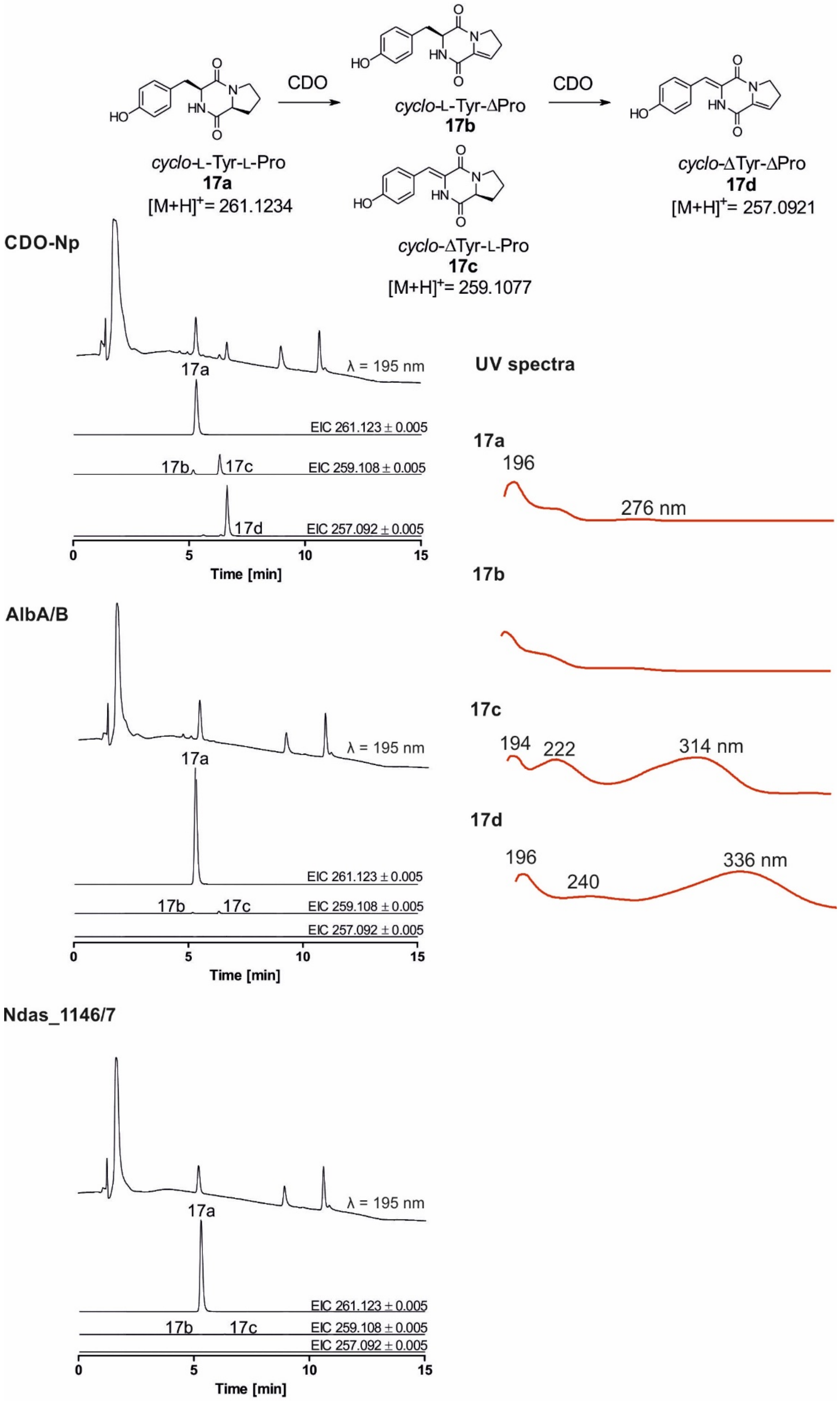

Figure S24: Overview of the conversion of **17a** to its dehydrogenated products by the 3 CDO-containing cell free extracts after 2h of incubation. The respective top chromatogram illustrates the UV absorption and the EICs for the substrate (**17a**), the di- (**17b** and **17c**), and tetradehydrogenated products (**17d**) are displayed below with a tolerance of ± 0.005. UV spectra of the substrate and products are placed on the right side.

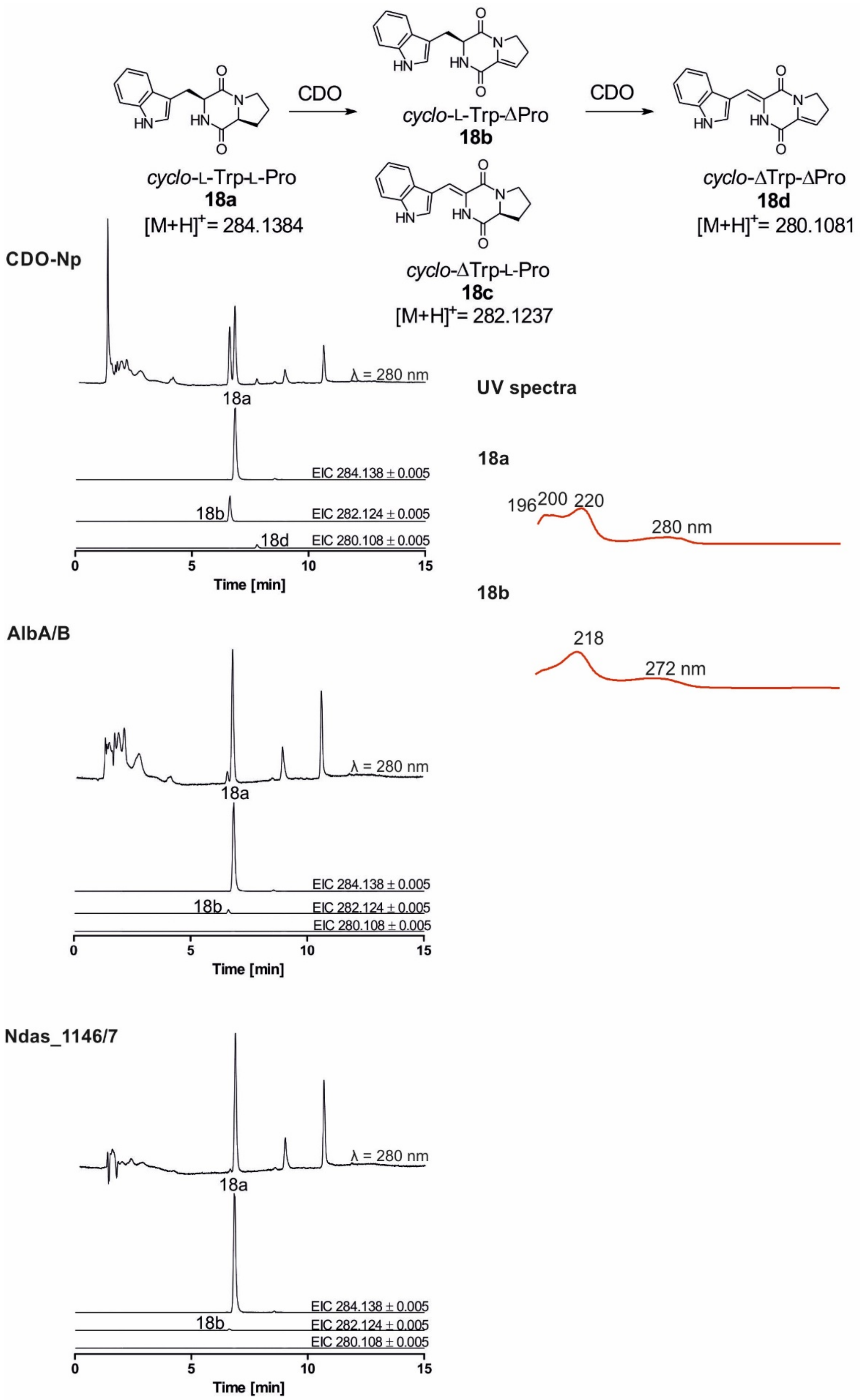

Figure S25: Overview of the conversion of **18a** to its dehydrogenated products by the 3 CDO-containing cell free extracts after 2h of incubation. The respective top chromatogram illustrates the UV absorption and the EICs for the substrate (**18a**), di- (**18b** and **18c**), and tetradehydrogenated products (**18d**) are displayed below with a tolerance of ± 0.005. UV spectra of the substrate and products are placed on the right side.

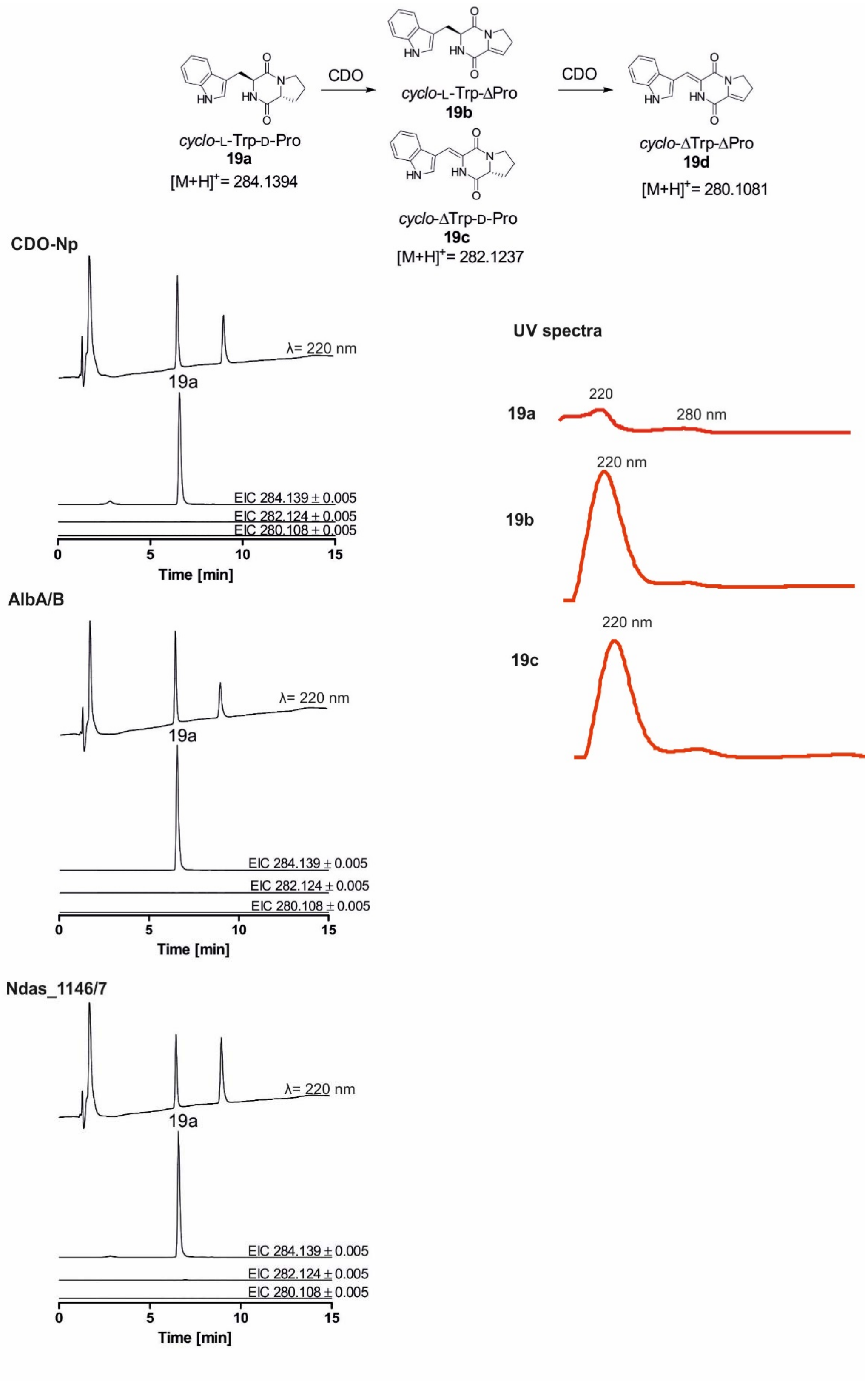

Figure S26: Overview of the conversion of **19a** to its dehydrogenated products by the 3 CDO-containing cell free extracts after 2h of incubation. The respective top chromatogram illustrates the UV absorption and the EICs for the substrate (**19a**), di- (**19b** and **19c**), and tetradehydrogenated products (**19d**) are displayed below with a tolerance of ± 0.005. UV spectra of the substrate and products are placed on the right side.

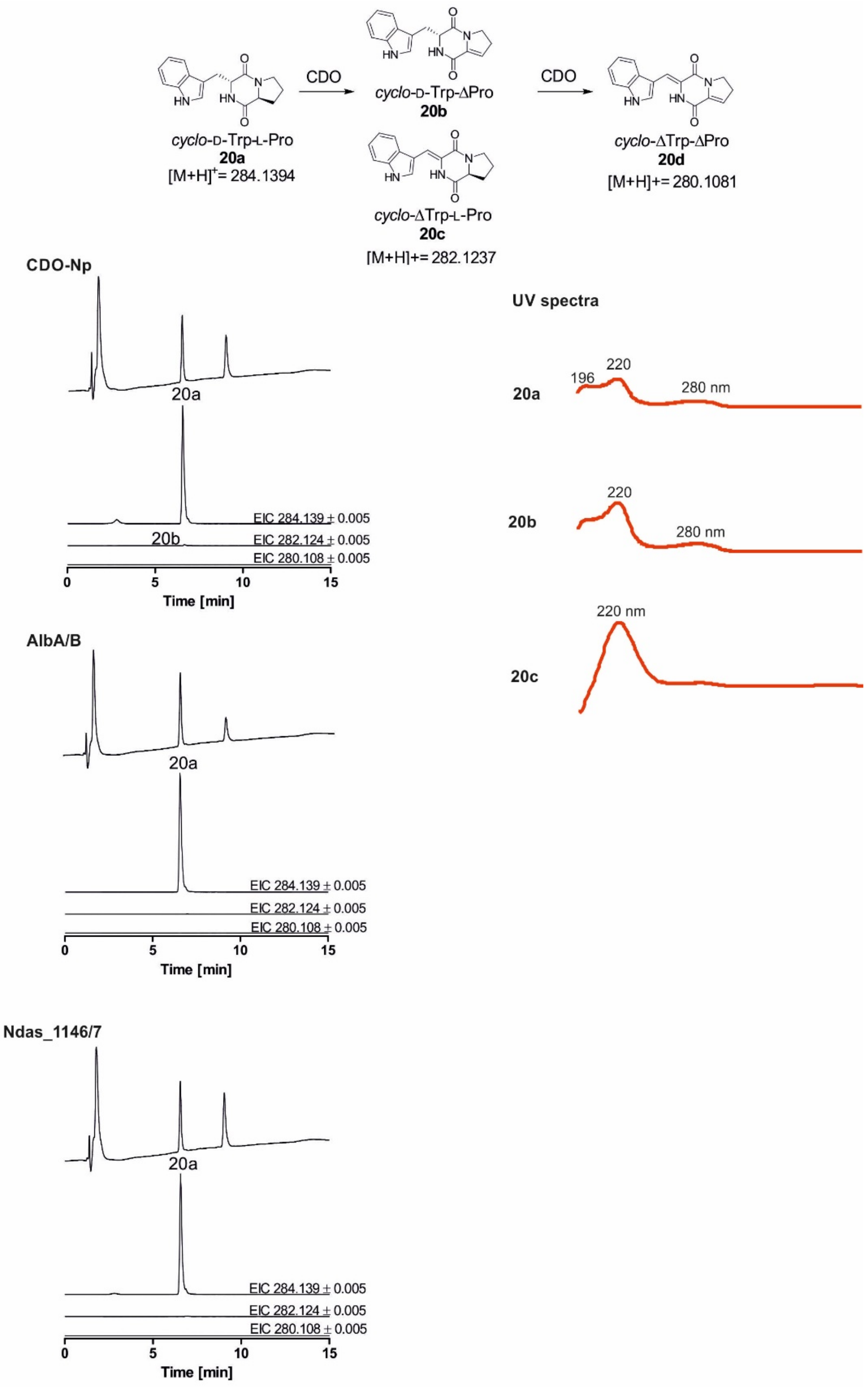

Figure S27: Overview of the conversion of **20a** to its dehydrogenated products by the 3 CDO-containing cell free extracts after 2h of incubation. The respective top chromatogram illustrates the UV absorption and the EICs for substrate (**20a**), di- (**20b** and **20c**), and tetradehydrogenated products (**20d**) are displayed below with a tolerance of ± 0.005. UV spectra of the substrate and products are placed on the right side.

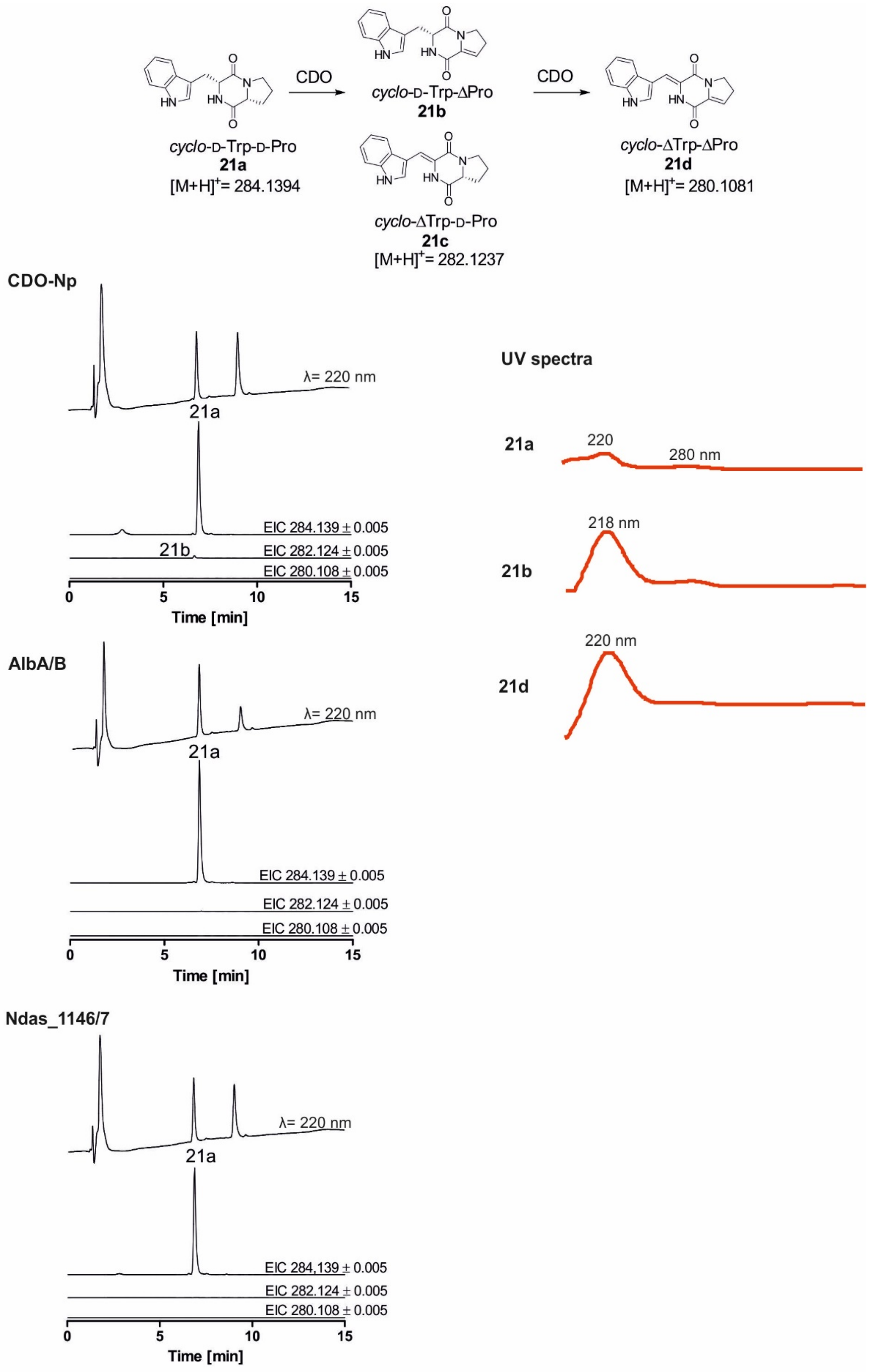

Figure S28: Overview of the conversion of **21a** to its dehydrogenated products by the 3 CDO-containing cell free extracts after 2h of incubation. The respective top chromatogram illustrates the UV absorption and the EICs for substrate (**21a**), di- (**21b** and **21c**), and tetradehydrogenated products (**21d**) are displayed below with a tolerance of ± 0.005. UV spectra of the substrate and products are placed on the right side.

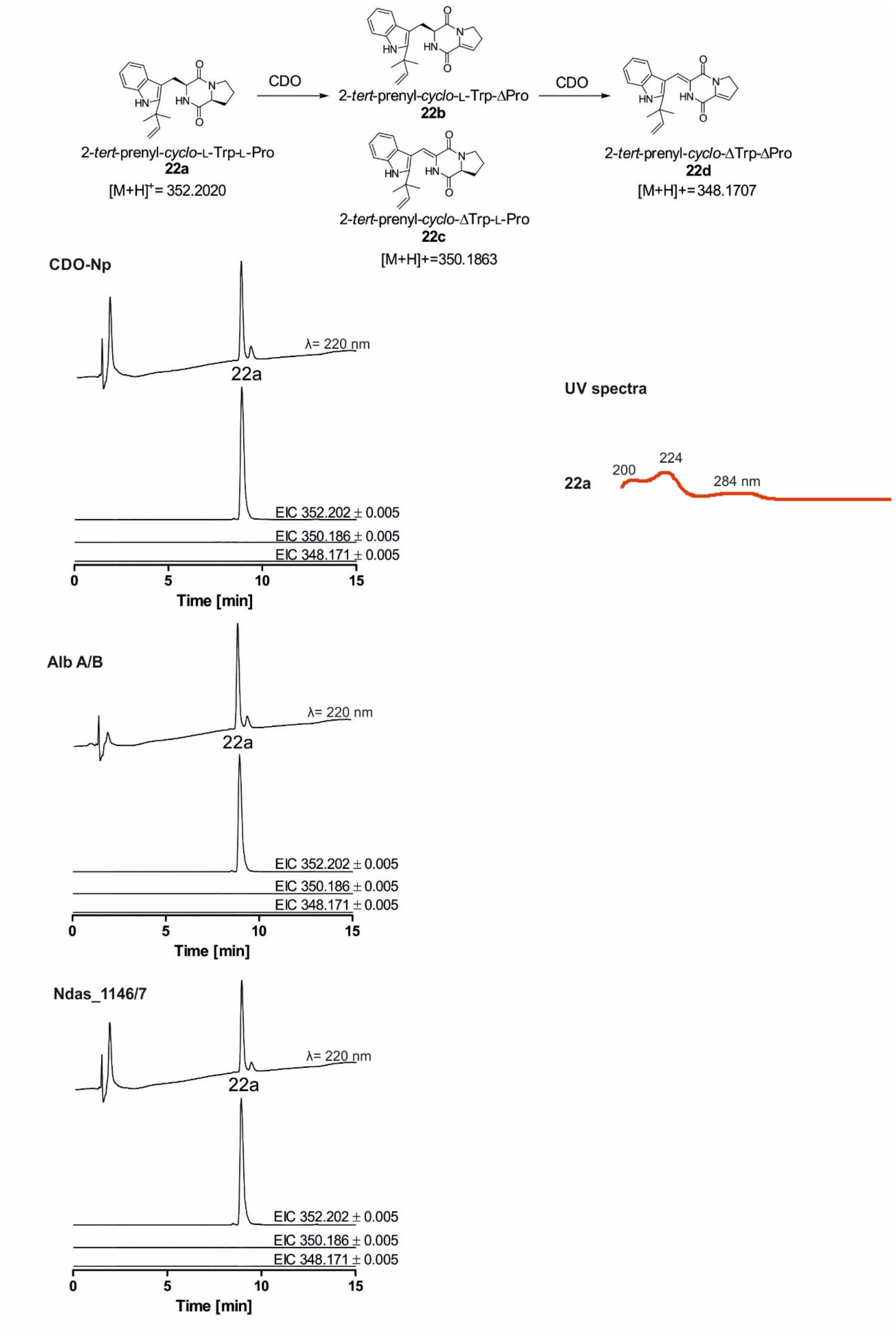

Figure S29: Overview of the conversion of **22a** to its dehydrogenated products by the 3 CDO-containing cell free extracts after 2h of incubation. The respective top chromatogram illustrates the UV absorption and the EICs for the substrate (**22a**), di- (**22b** and **22c**), and tetradehydrogenated products (**22d**) are displayed below with a tolerance of ± 0.005. UV spectra of the substrate and products are placed on the right side.

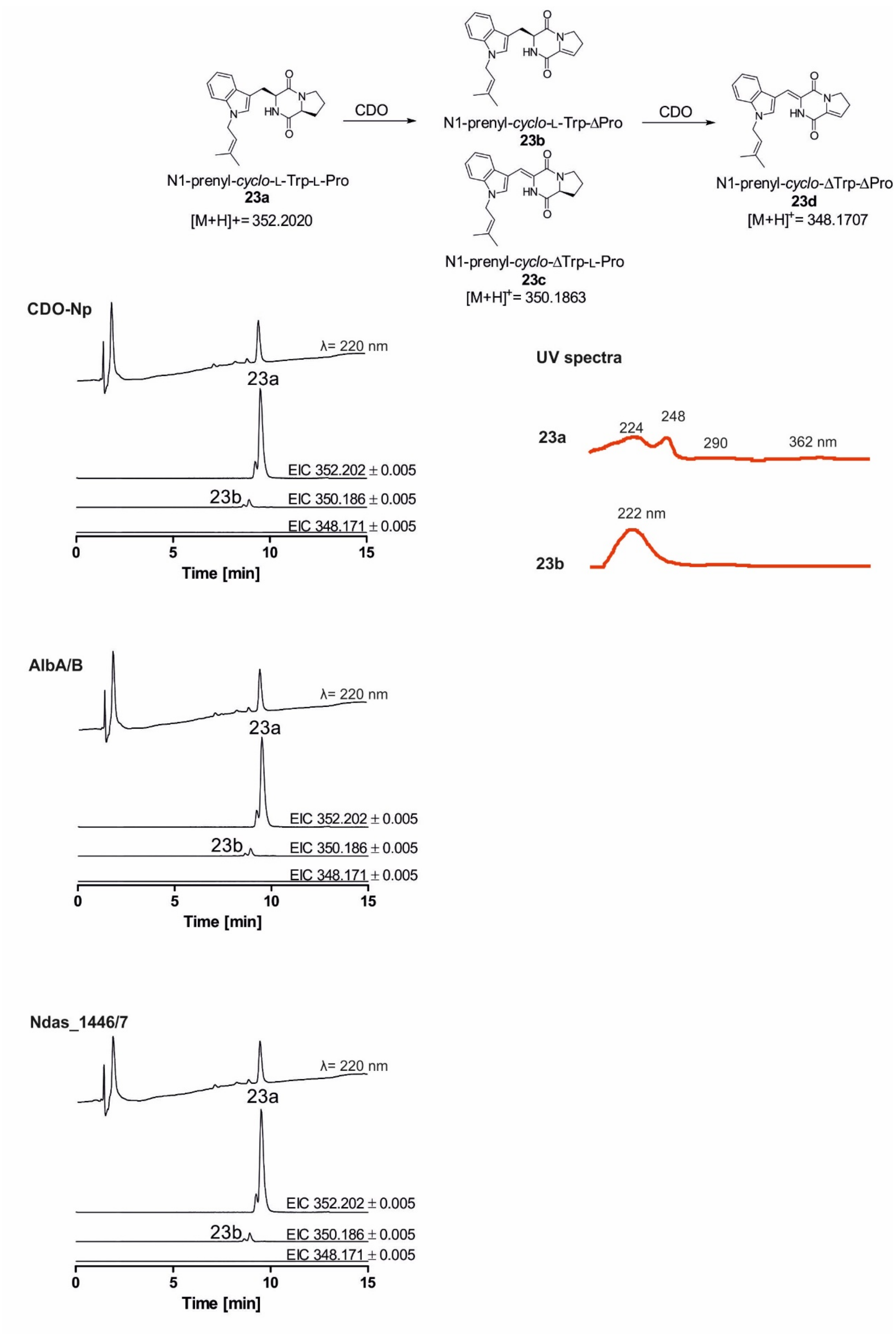

Figure S30: Overview of the conversion of **23a** to its dehydrogenated products by the 3 CDO-containing cell free extracts after 2h of incubation. The respective top chromatogram illustrates the UV absorption and the EICs for substrate (**23a**), di- (**23b** and **23c**), and tetradehydrogenated products (**23d**) are displayed below with a tolerance of ± 0.005. UV spectra of the substrate and products are placed on the right side.

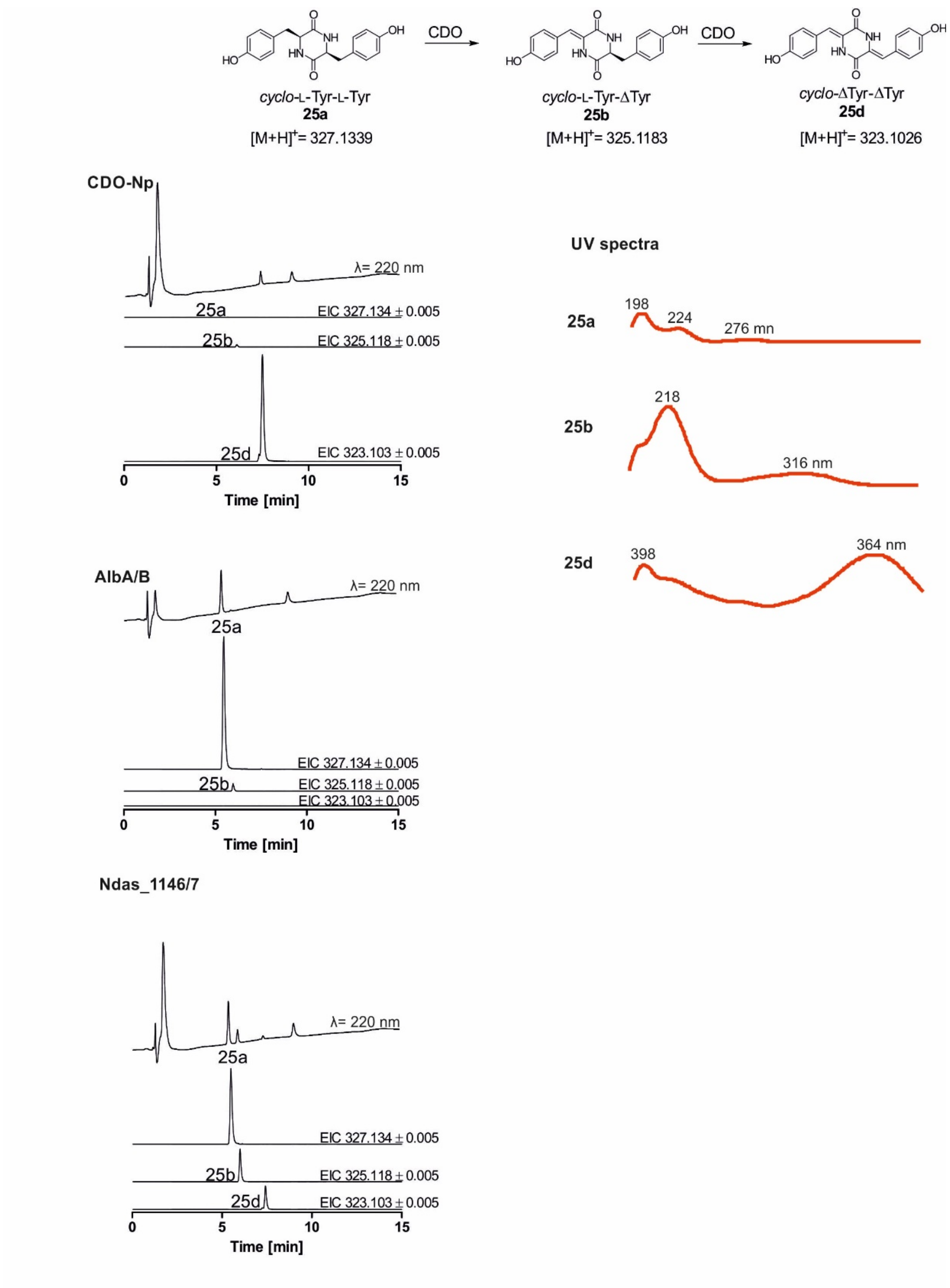

Figure S31: Overview of the conversion of **25a** to its dehydrogenated products by the 3 CDO-containing cell free extracts after 2h of incubation. The respective top chromatogram illustrates the UV absorption and the EICs for substrate (**25a**), di- (**25b** and **25c**), and tetradehydrogenated products (**25d**) are displayed below with a tolerance of ± 0.005. UV spectra of the substrate and products are placed on the right side.

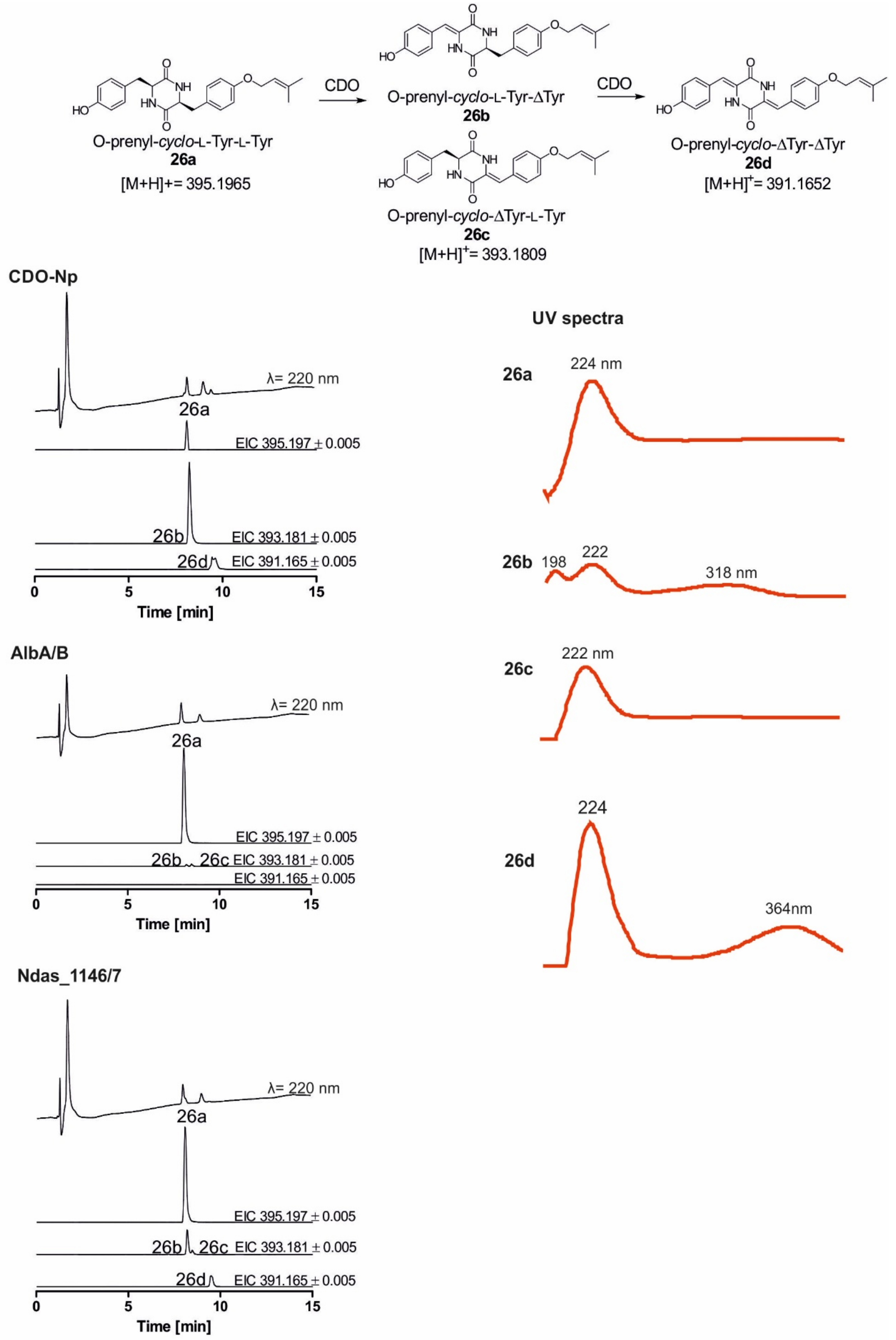

Figure S32: Overview of the conversion of **26a** to its dehydrogenated products by the 3 CDO-containing cell free extracts after 2h of incubation. The respective top chromatogram illustrates the UV absorption and the EICs for substrate (**26a**), di- (**26b** and **26c**), and tetradehydrogenated products (**26d**) are displayed below with a tolerance of ± 0.005. UV spectra of the substrate and products are placed on the right side.

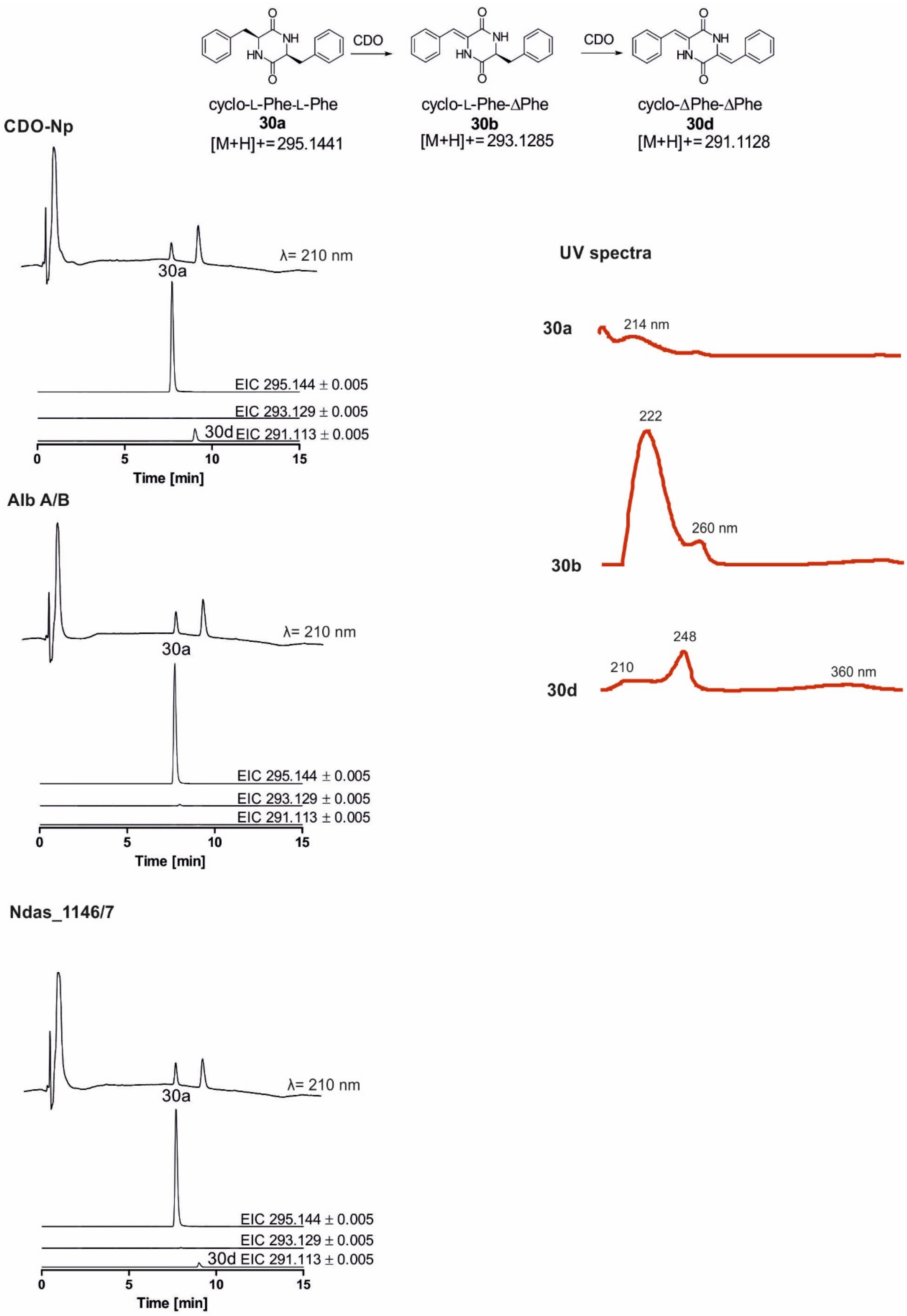

Figure S33: Overview of the conversion of **30a** to its dehydrogenated products by the 3 CDO-containing cell free extracts after 2h of incubation. The respective top chromatogram illustrates the UV absorption and the EICs for substrate (**30a**), di- (**30b** and **30c**), and tetradehydrogenated products (**30d**) are displayed below with a tolerance of ± 0.005. UV spectra of the substrate and products are placed on the right side.

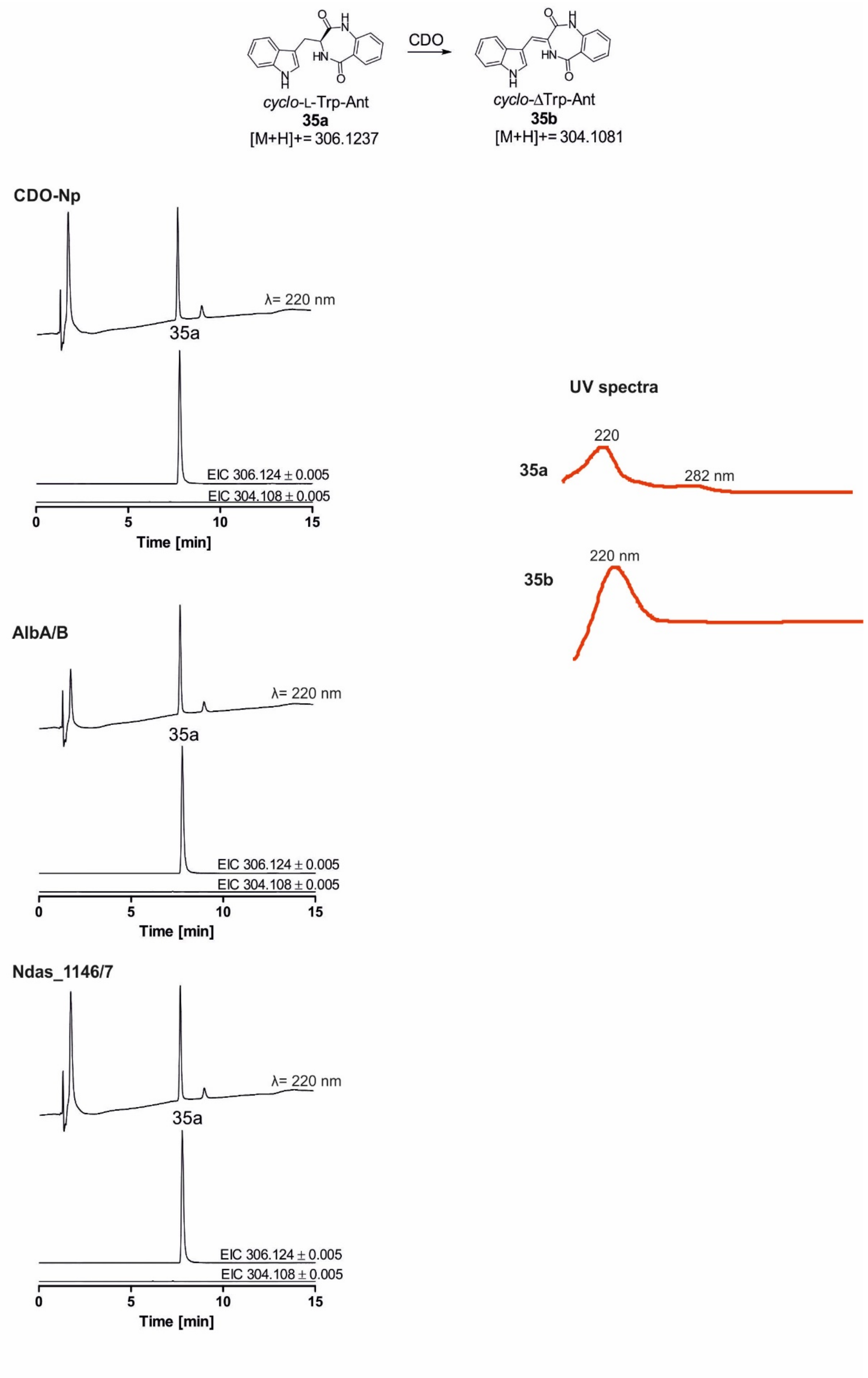

Figure S34: Overview of the conversion of **35a** to its dehydrogenated products by the 3 CDO-containing cell free extracts after 2h of incubation. The respective top chromatogram illustrates the UV absorption and the EICs for substrate (**35a**), di- (**35b** and **35c**), and tetradehydrogenated products (**35d**) are displayed below with a tolerance of ± 0.005. UV spectra of the substrate and products are placed on the right side.

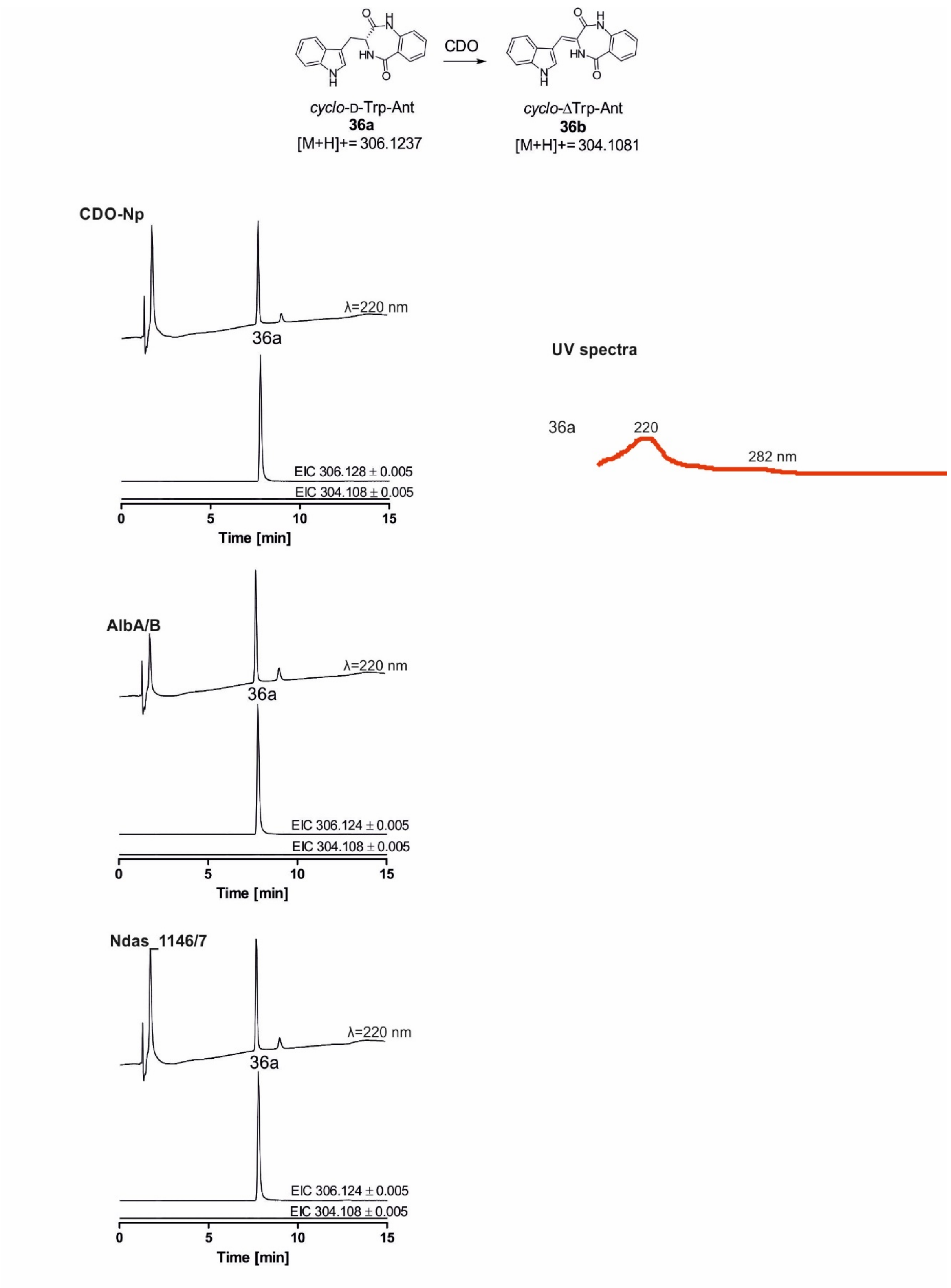

Figure S35: Overview of the conversion of **36a** to its dehydrogenated products by the 3 CDO-containing cell free extracts after 2h of incubation. The respective top chromatogram illustrates the UV absorption and the EICs for substrate (**36a**), di- (**36b** and **36c**), and tetradehydrogenated products (**36d**) are displayed below with a tolerance of ± 0.005. UV spectra of the substrate and products are placed on the right side.

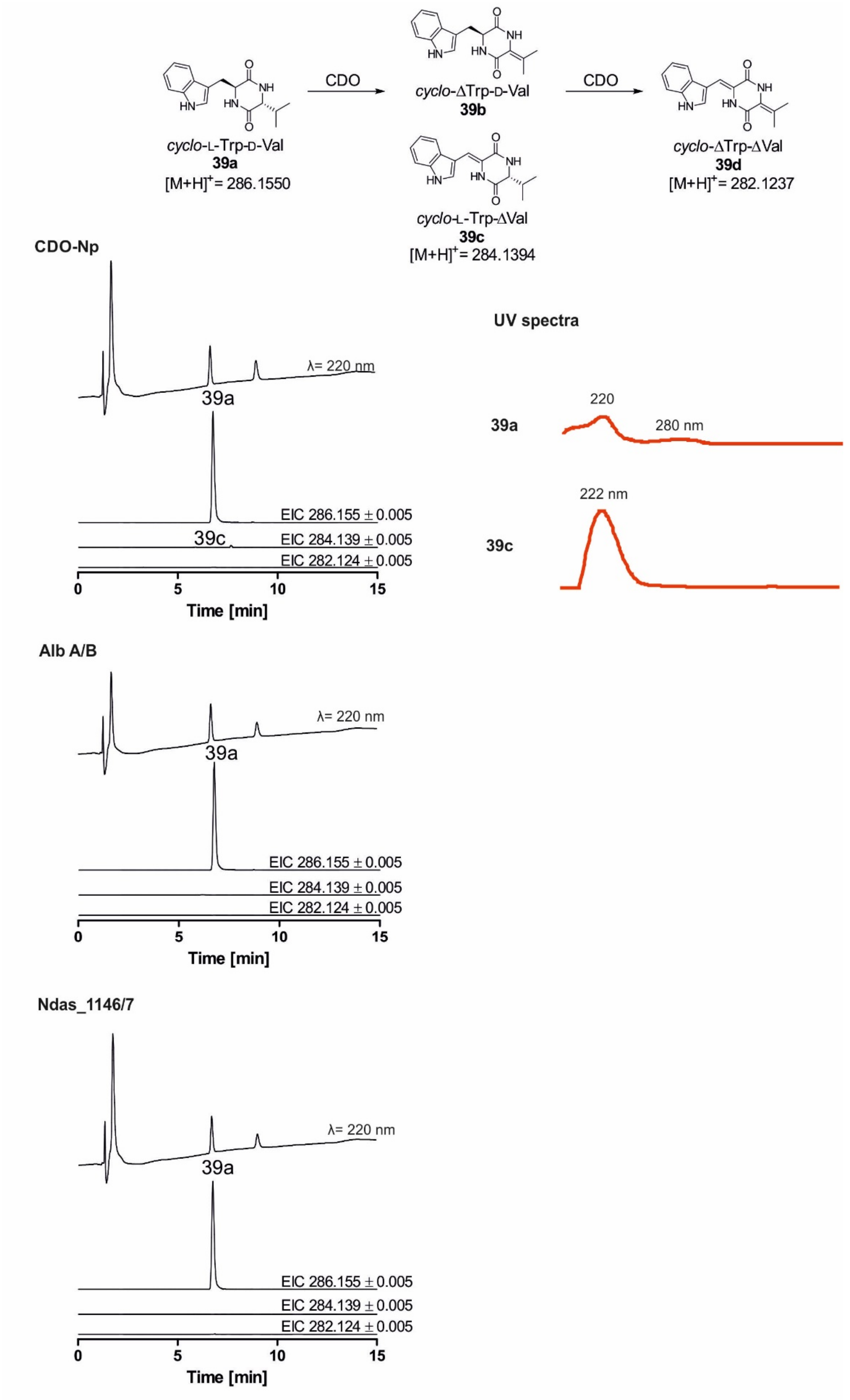

Figure S36: Overview of the conversion of **39a** to its dehydrogenated products by the 3 CDO-containing cell free extracts after 2h of incubation. The respective top chromatogram illustrates the UV absorption and the EICs for substrate (**39a**), di- (**39b** and **39c**), and tetradehydrogenated products (**39d**) are displayed below with a tolerance of ± 0.005. UV spectra of the substrate and products are placed on the right side.

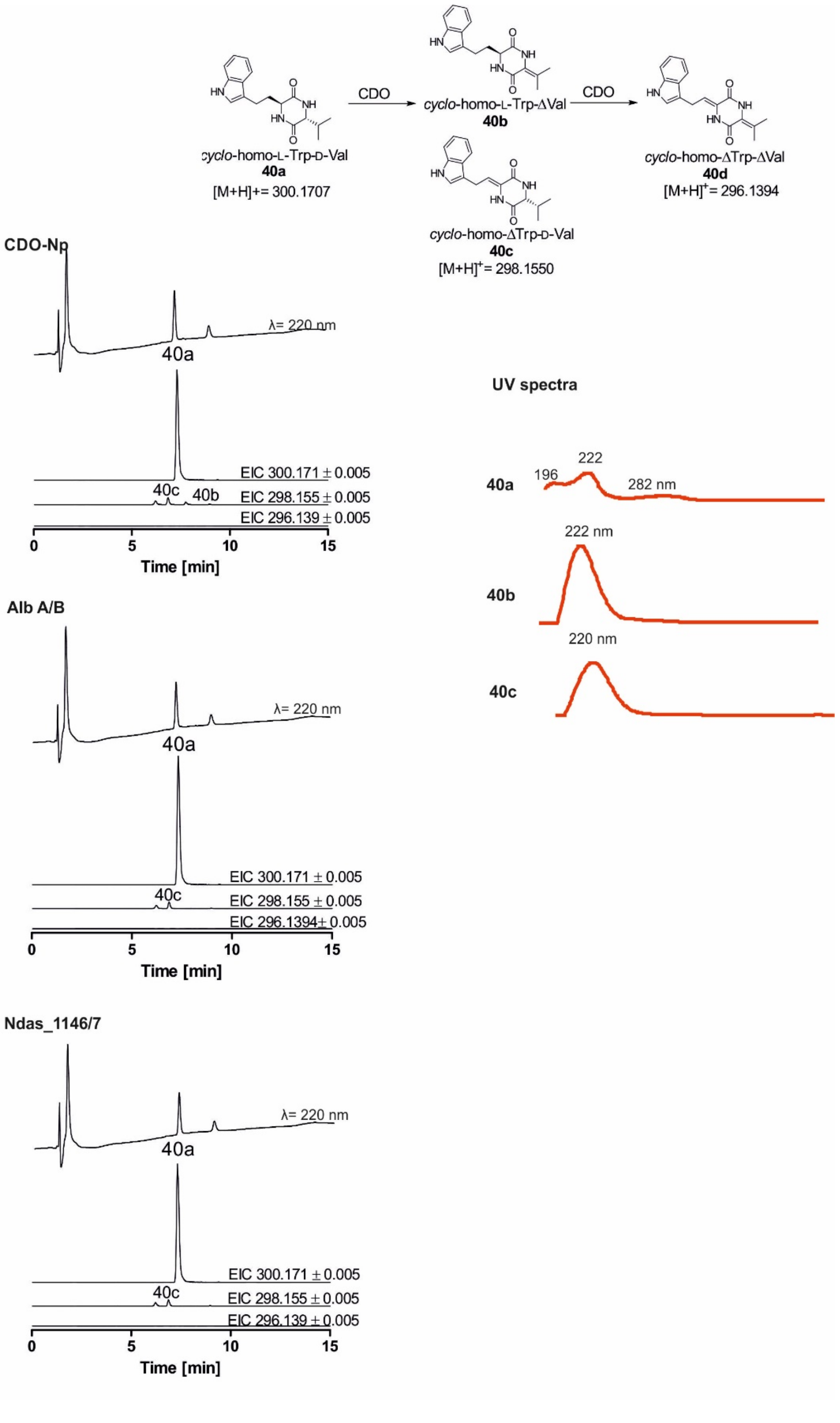

Figure S37: Overview of the conversion of **40a** to its dehydrogenated products by the 3 CDO-containing cell free extracts after 2h of incubation. The respective top chromatogram illustrates the UV absorption and the EICs for substrate (**40a**), di- (**40b** and **40c**), and tetradehydrogenated products (**40d**) are displayed below with a tolerance of ± 0.005. UV spectra of the substrate and products are placed on the right side.

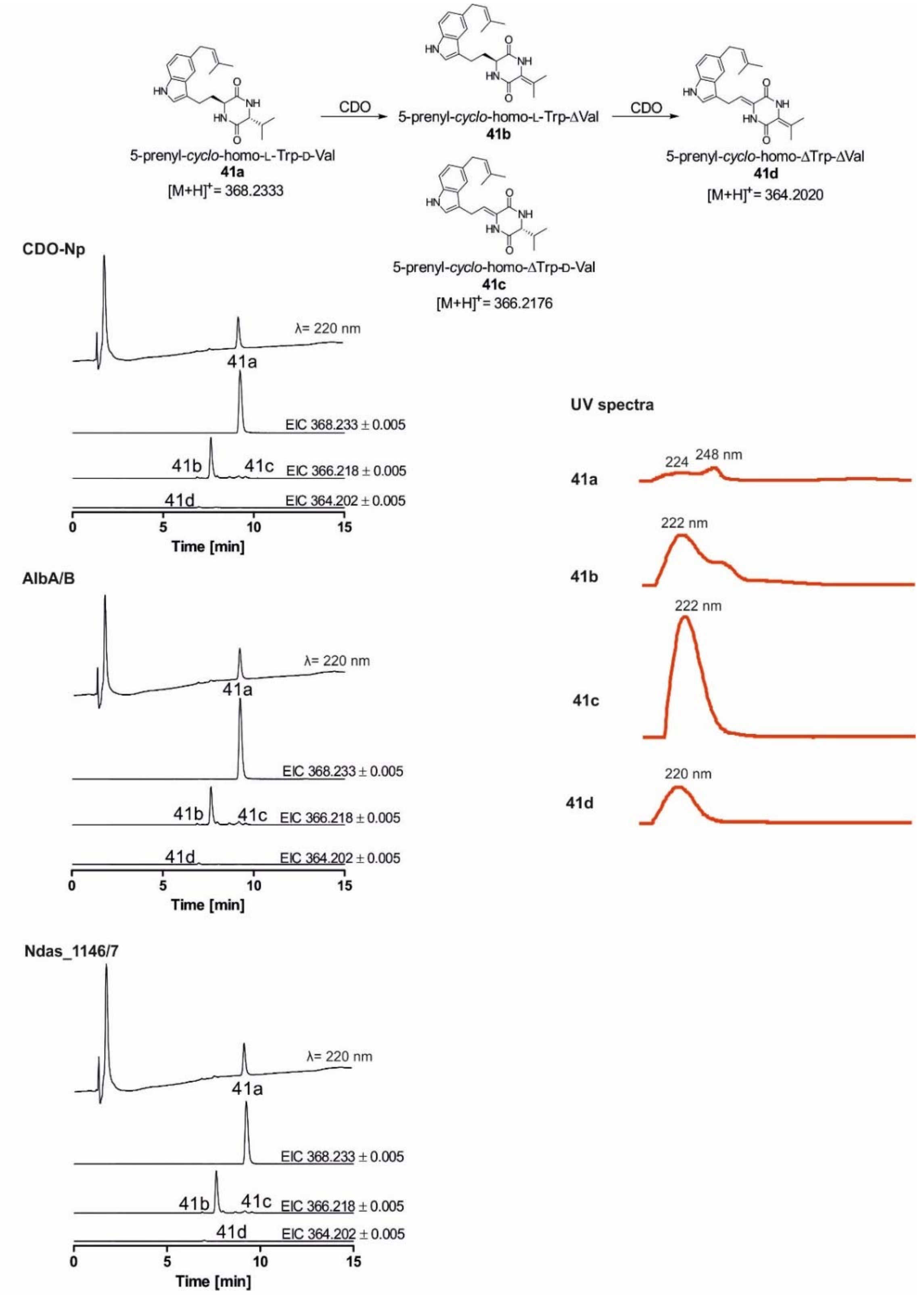

Figure S38: Overview of the conversion of **41a** to its dehydrogenated products by the 3 CDO-containing cell free extracts after 2h of incubation. The respective top chromatogram illustrates the UV absorption and the EICs for substrate (**41a**), di- (**41b** and **41c**), and tetradehydrogenated products (**41d**) are displayed below with a tolerance of ± 0.005. UV spectra of the substrate and products are placed on the right side.

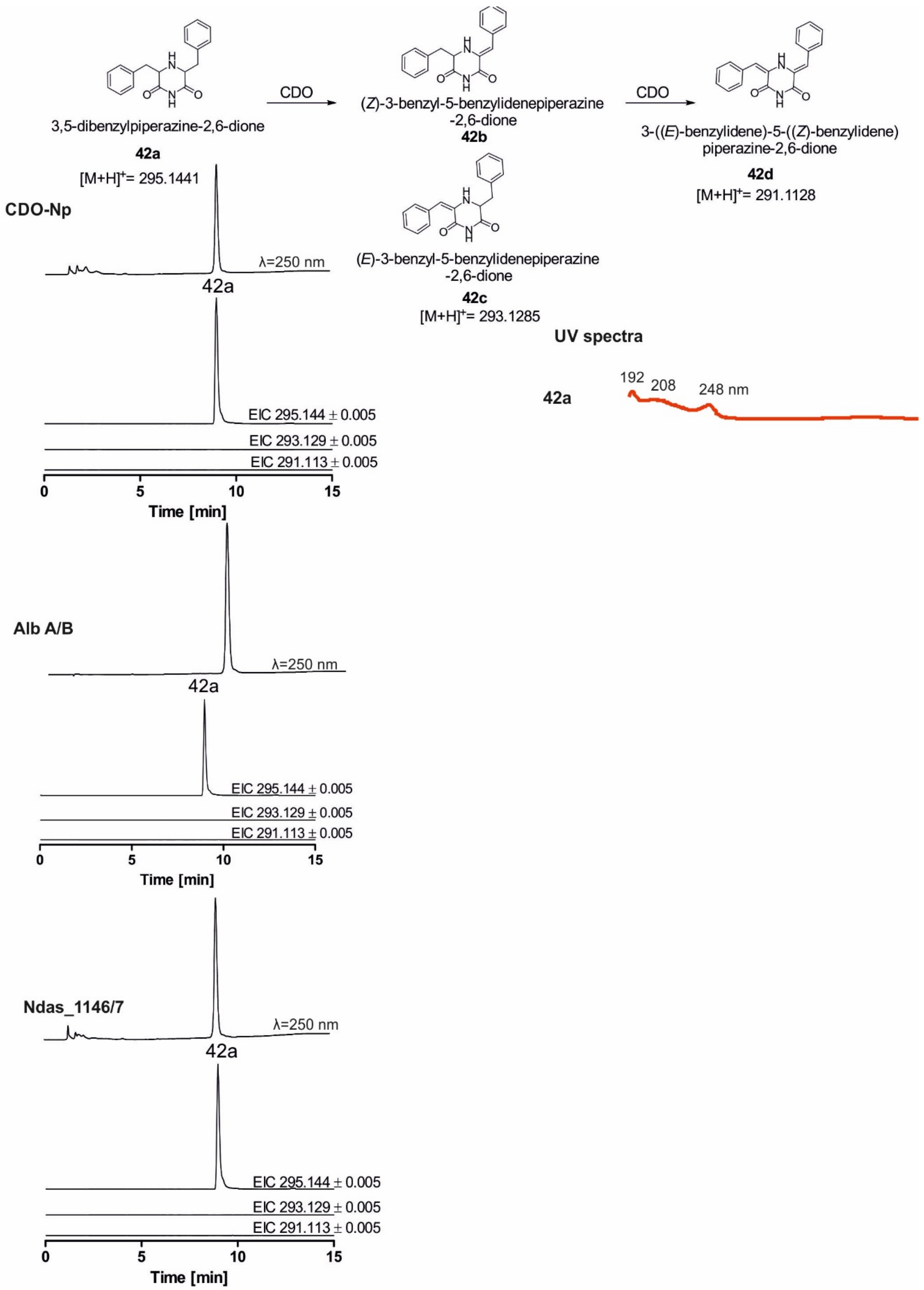

Figure S39: Overview of the conversion of **42a** to its dehydrogenated products by the 3 CDO-containing cell free extracts after 2h of incubation. The respective top chromatogram illustrates the UV absorption and the EICs for substrate (**42a**), di- (**42b** and **42c**), and tetradehydrogenated products (**42d**) are displayed below with a tolerance of ± 0.005. UV spectra of the substrate and products are placed on the right side.

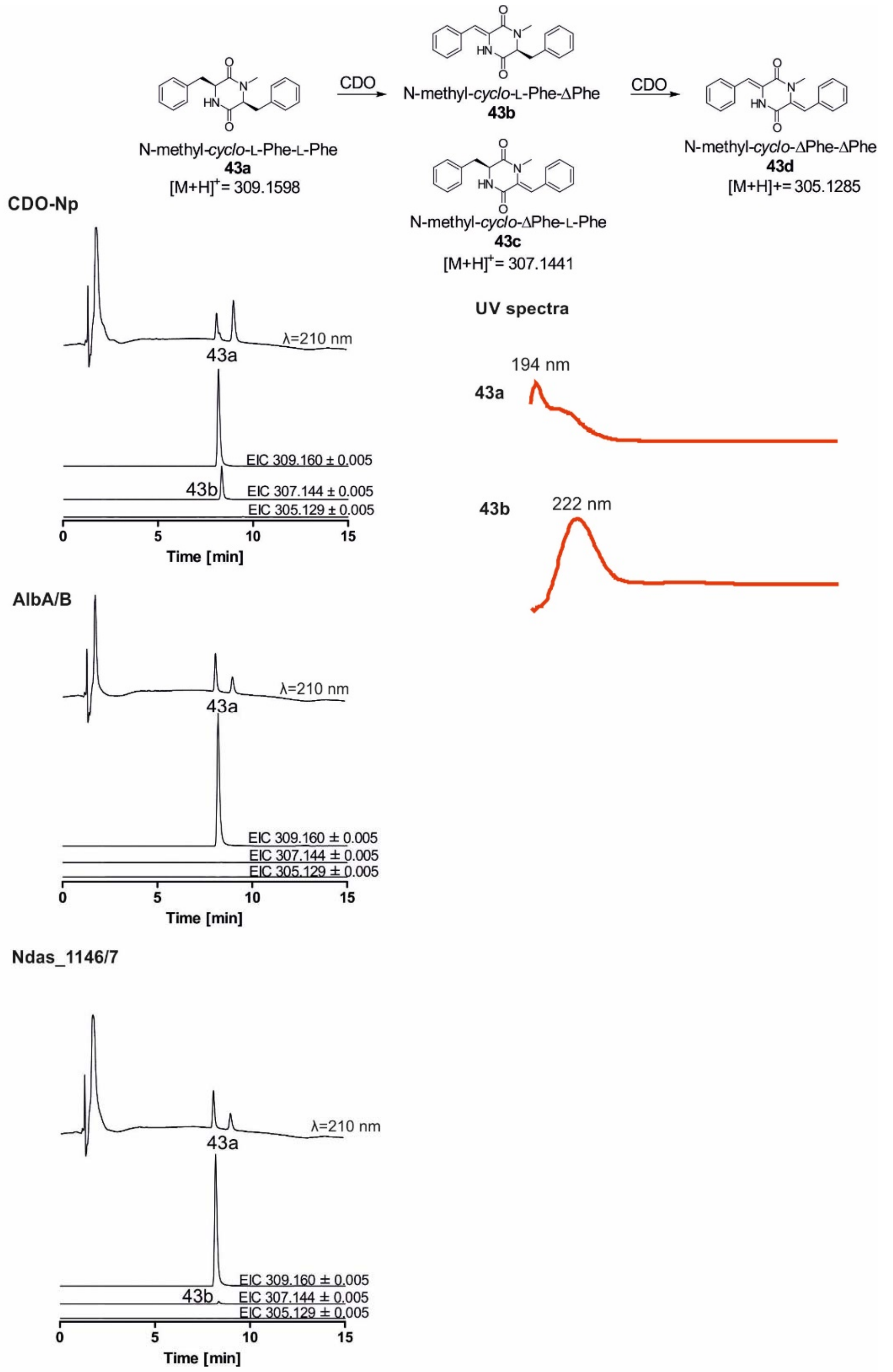

Figure S40: Overview of the conversion of **43a** to its dehydrogenated products by the 3 CDO-containing cell free extracts after 2h of incubation. The respective top chromatogram illustrates the UV absorption and the EICs for substrate (**43a**), di- (**43b** and **43c**), and tetradehydrogenated products (**43d**) are displayed below with a tolerance of ± 0.005. UV spectra of the substrate and products are placed on the right side.

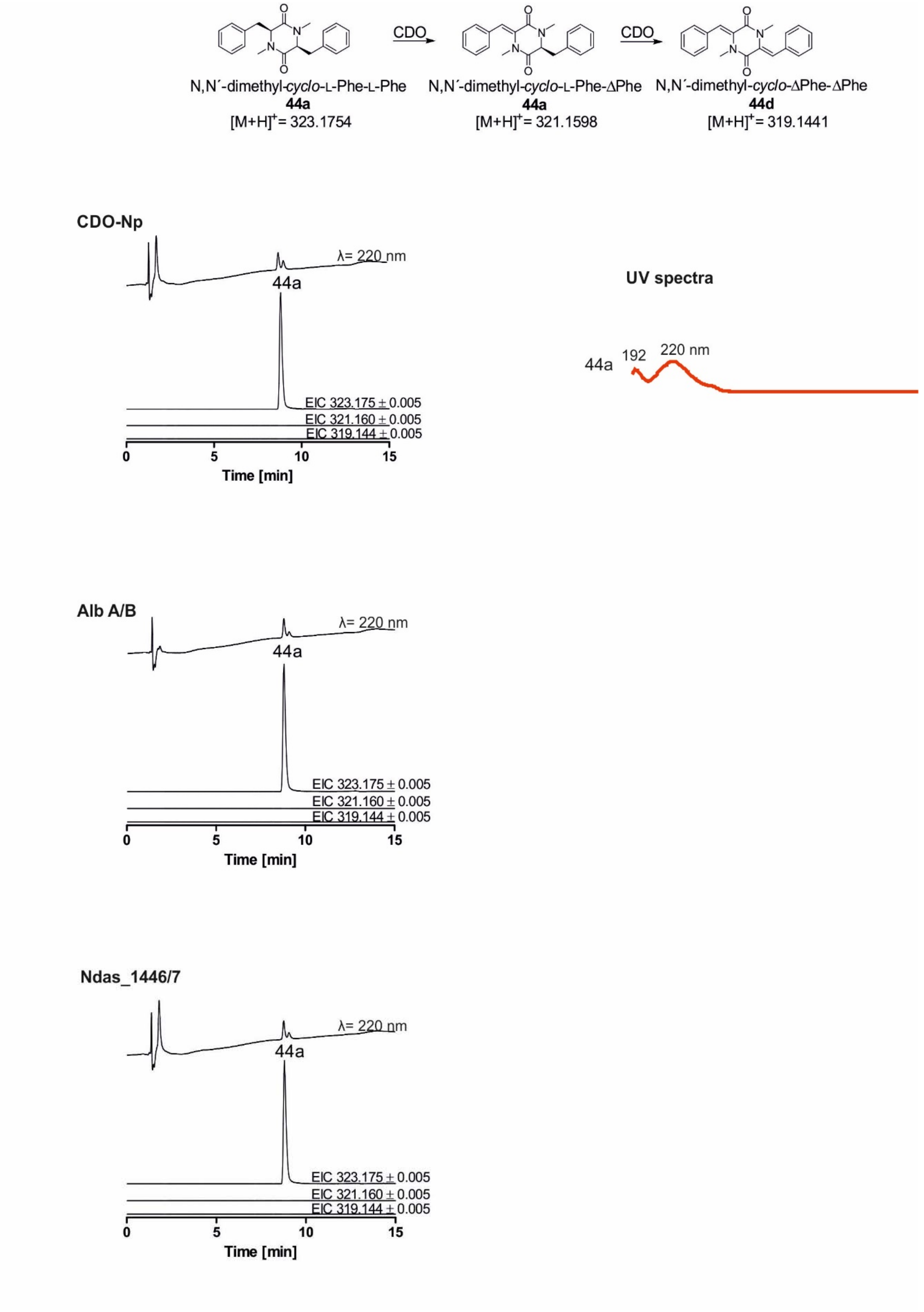

Figure S41: Overview of the conversion of **44a** to its dehydrogenated products by the 3 CDO-containing cell free extracts after 2h of incubation. The respective top chromatogram illustrates the UV absorption and the EICs for substrate (**44a**), di- (**44b** and **44c**), and tetradehydrogenated products (**44d**) are displayed below with a tolerance of ± 0.005. UV spectra of the substrate and products are placed on the right side.

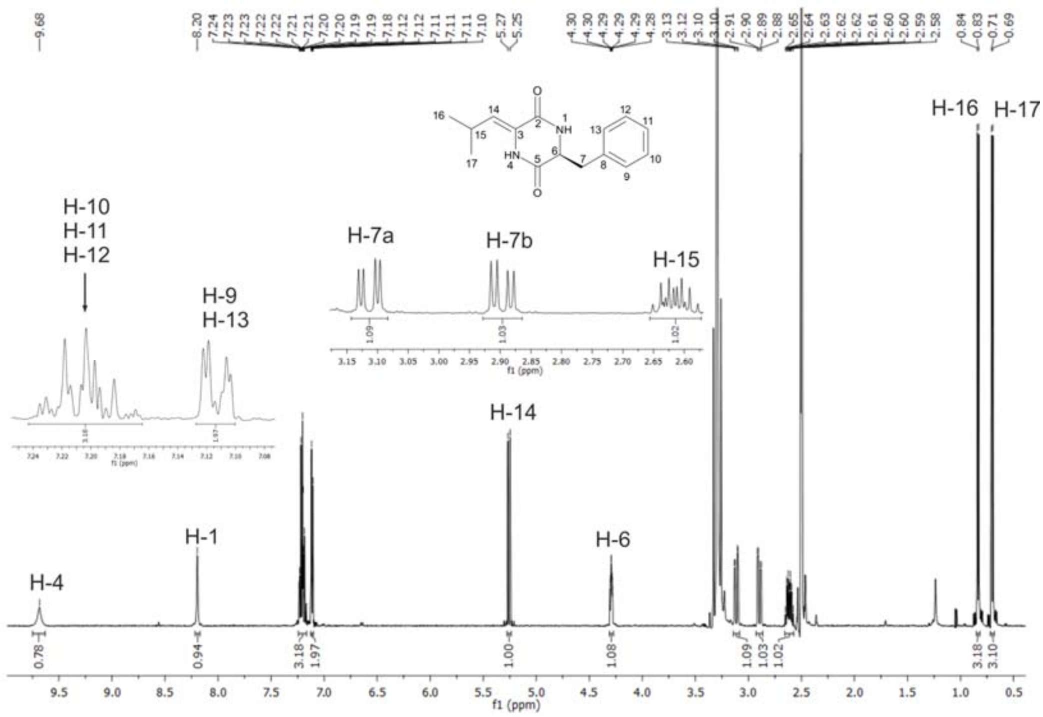

Figure S42: ^1H NMR spectrum of **1b** in DMSO-d_6 (500 MHz).

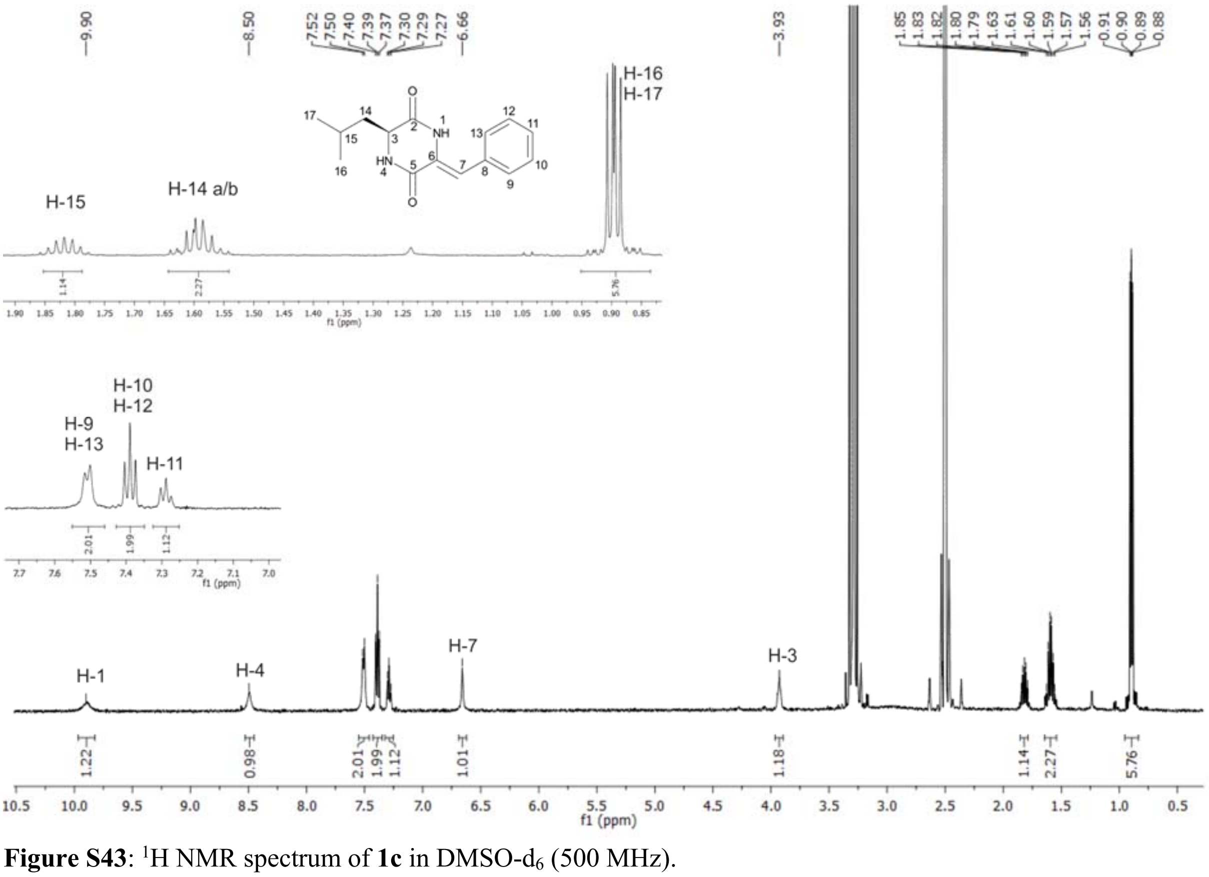

Figure S43: ^1H NMR spectrum of **1c** in DMSO-d_6 (500 MHz).

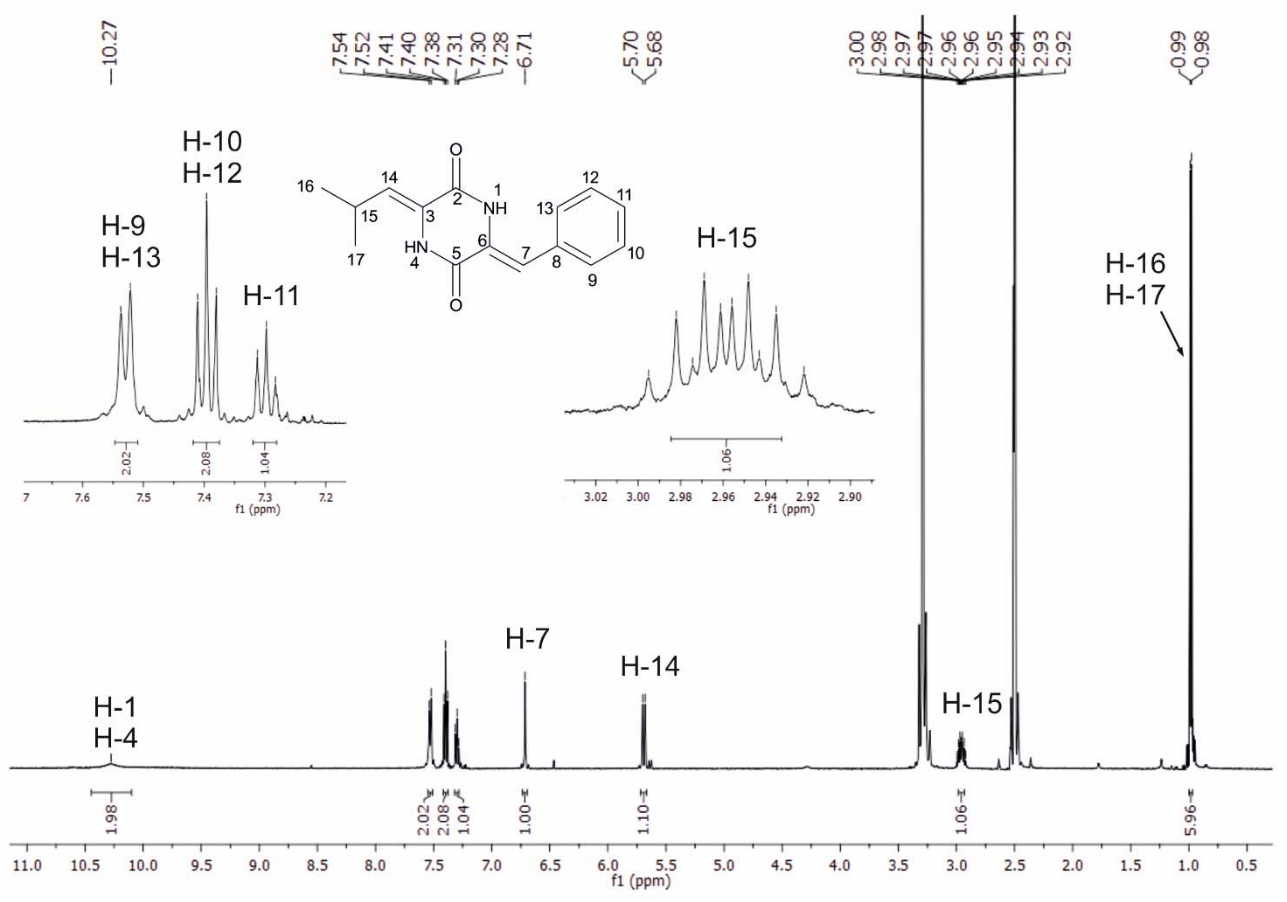

Figure S44: ^{1}H NMR spectrum of **1d** in DMSO-d_6 (500 MHz).

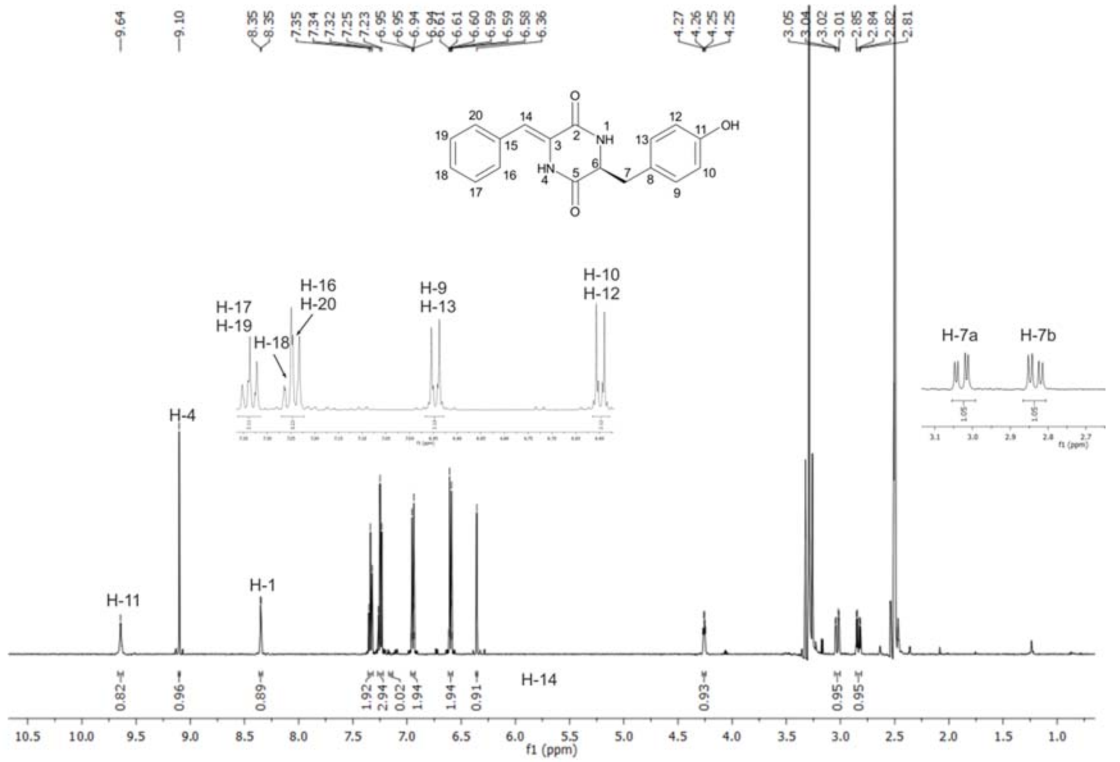

Figure S45: ^{1}H NMR spectrum of **2b** in DMSO-d_6 (500 MHz).

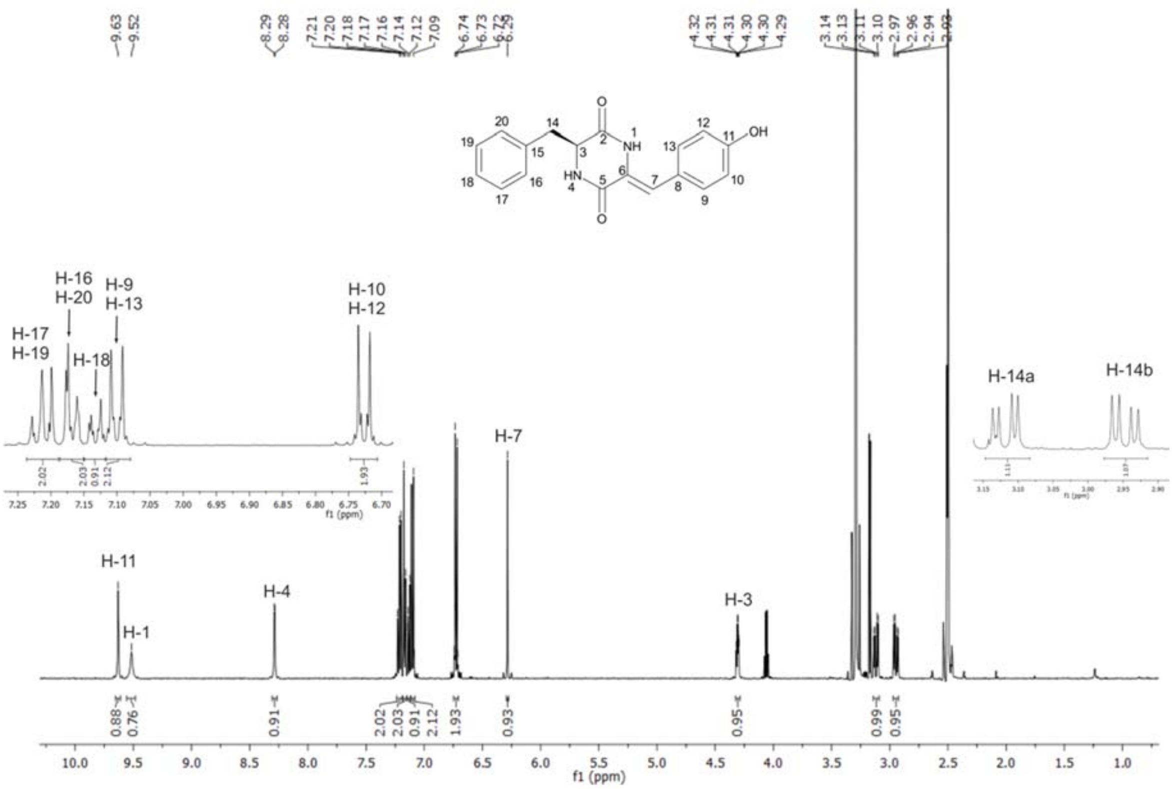

Figure S46: ^{1}H NMR spectrum of **2c** in DMSO-d$_6$ (500 MHz).

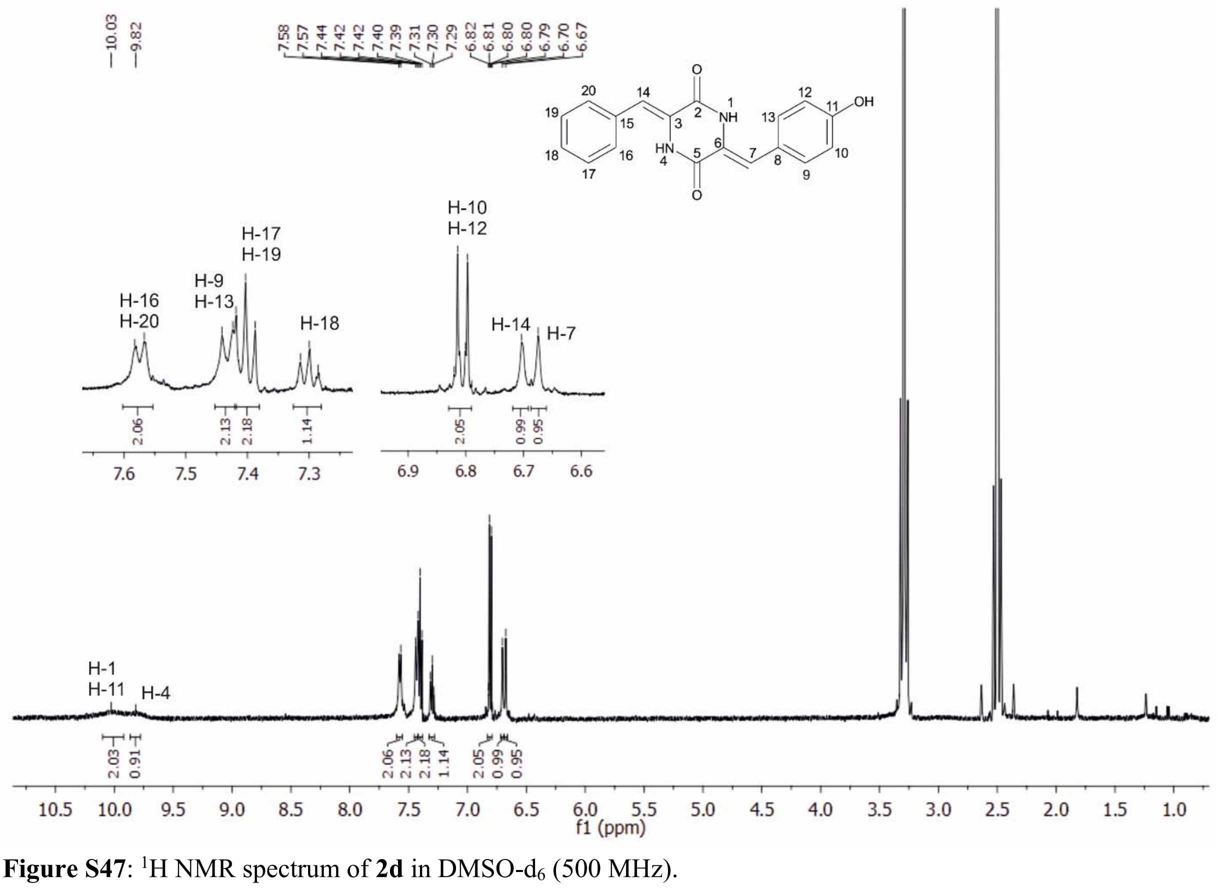

Figure S47: ^{1}H NMR spectrum of **2d** in DMSO-d$_6$ (500 MHz).

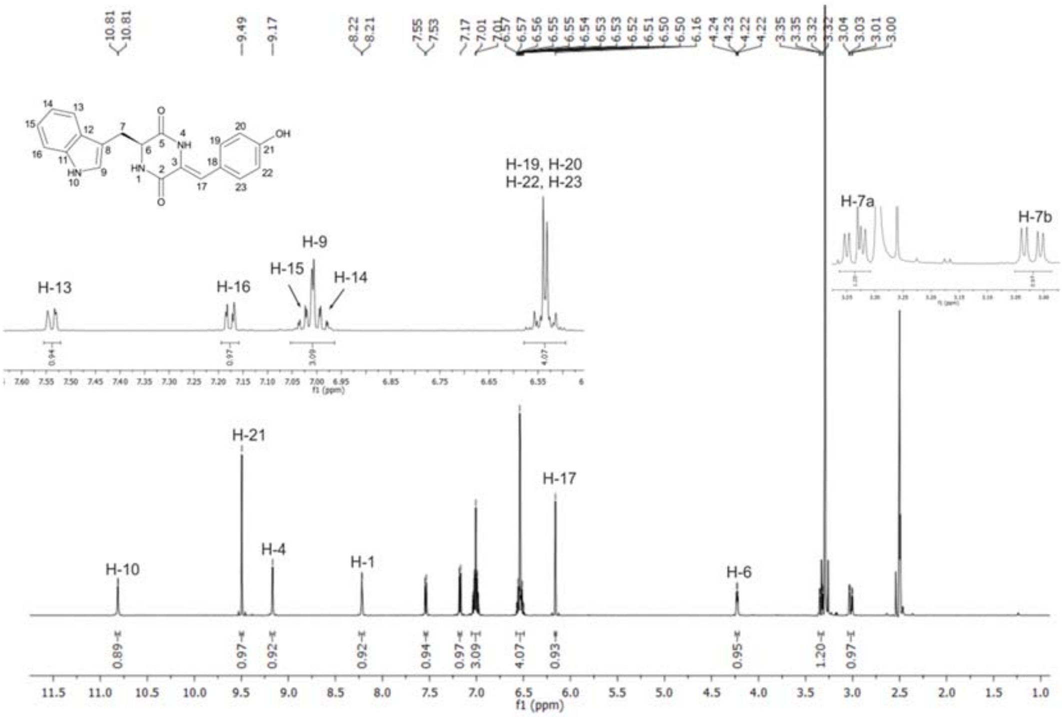

Figure S48: ^{1}H NMR spectrum of **3b** in DMSO-d$_6$ (500 MHz).

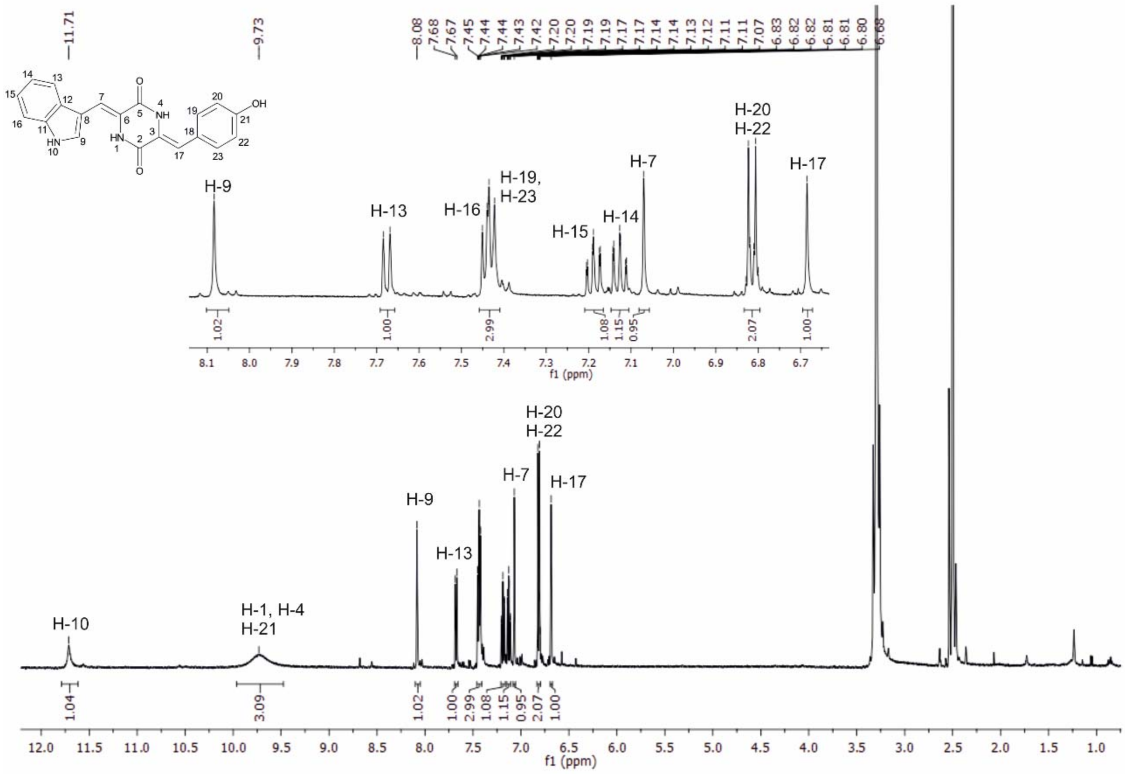

Figure S49: ^{1}H NMR spectrum of **3d** in DMSO-d$_6$ (500 MHz).

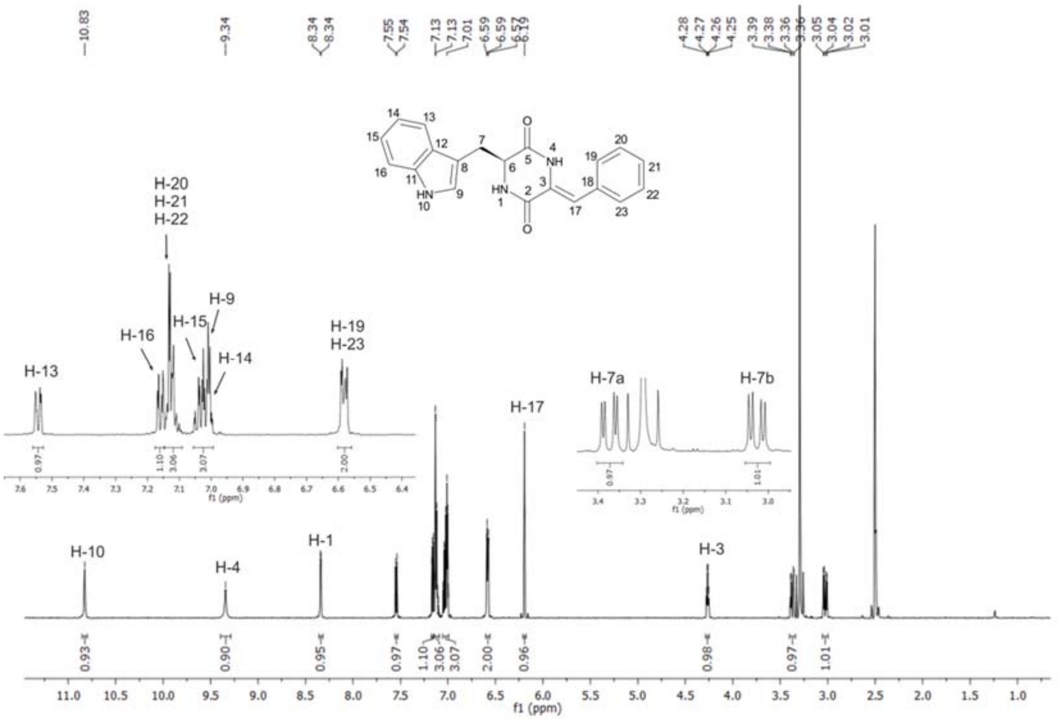

Figure S50: ^{1}H NMR spectrum of **5b** in DMSO-d$_6$ (500 MHz).

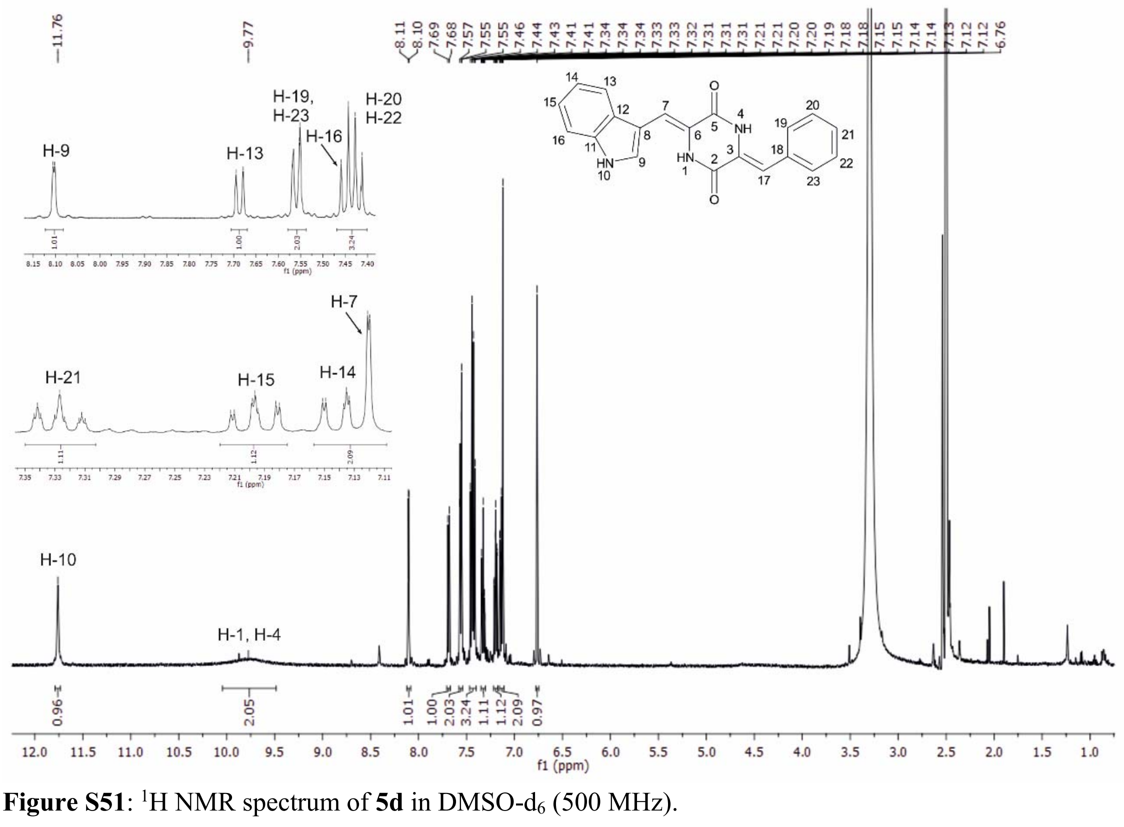

Figure S51: ^{1}H NMR spectrum of **5d** in DMSO-d$_6$ (500 MHz).

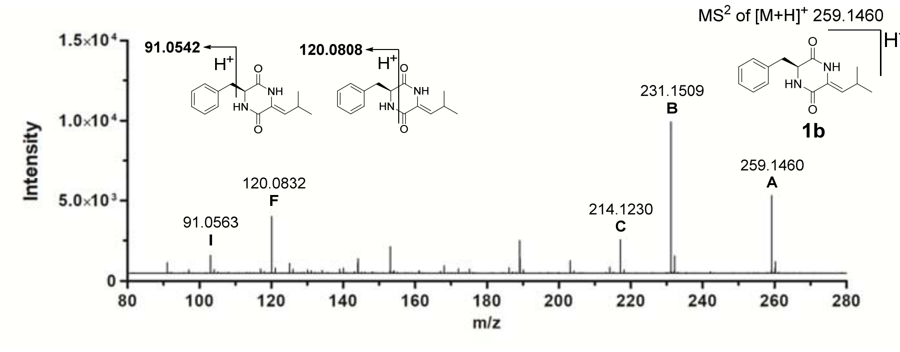

Figure S52: MS^2 spectrum of **1b**.

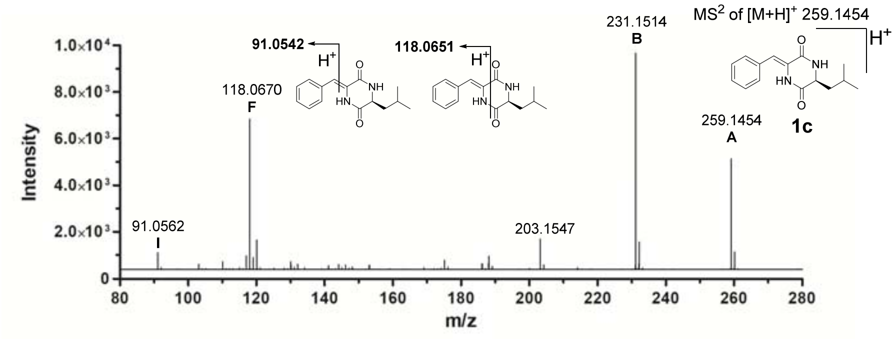

Figure S53: MS^2 spectrum of **1c**.

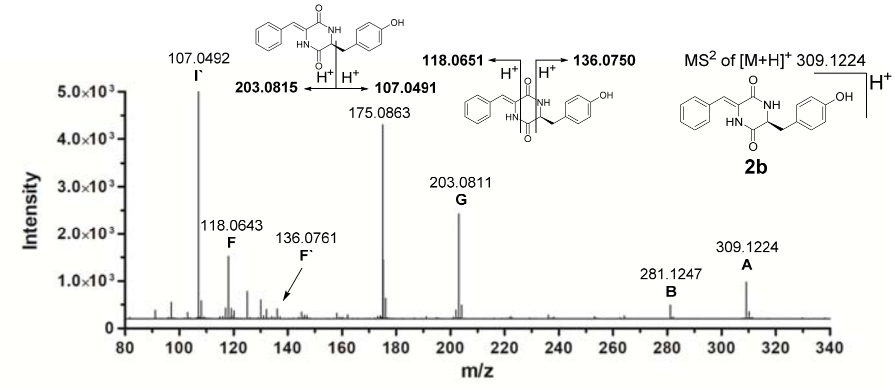

Figure S54: MS^2 spectrum of **2b**.

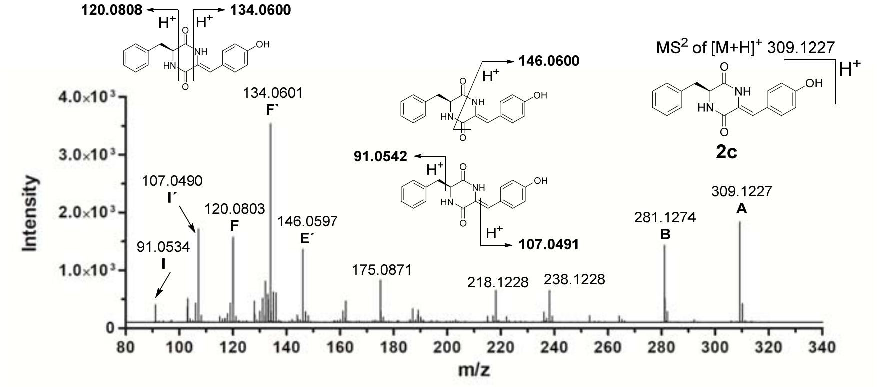

Figure S55: MS^2 spectrum of **2c**.

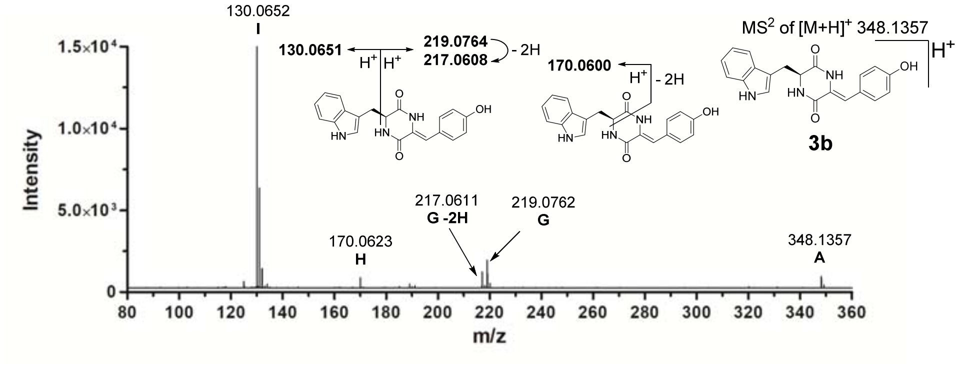

Figure S56: MS^2 spectrum of **3b**.

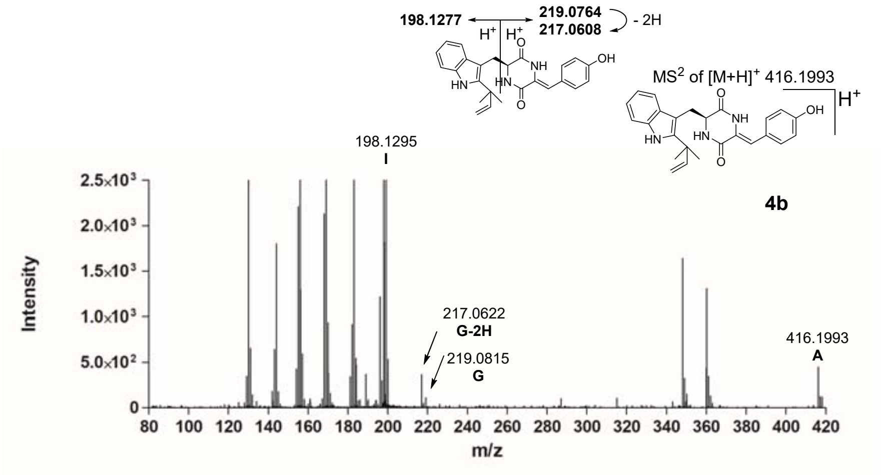

Figure S57: MS^2 spectrum of **4b**

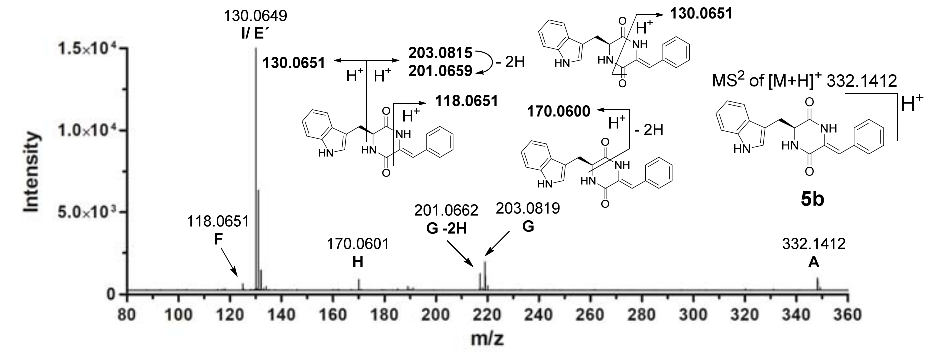

Figure S58: MS^2 spectrum of **5b**.

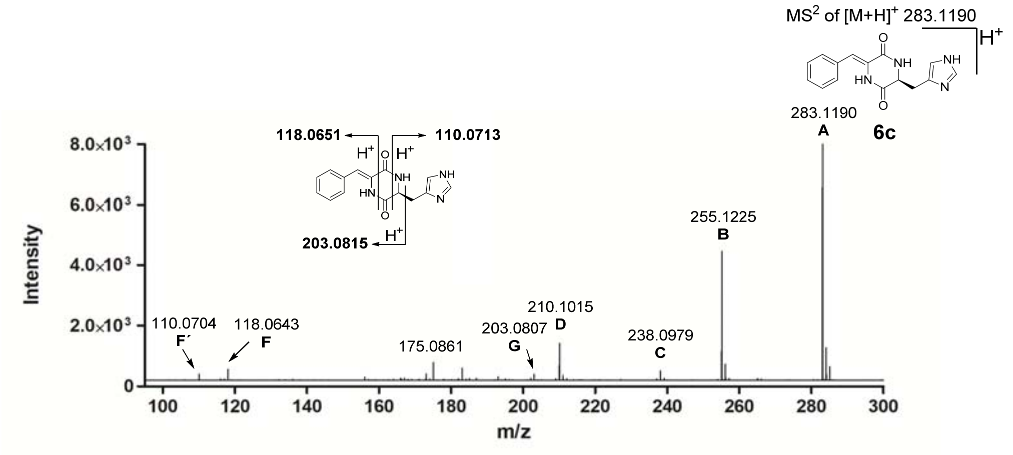

Figure S59: MS^2 spectrum of **6c**.

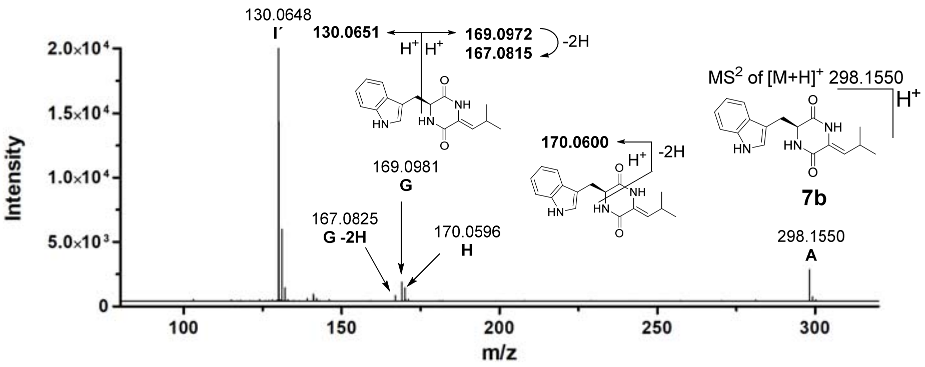

Figure S60: MS^2 spectrum of **7b**.

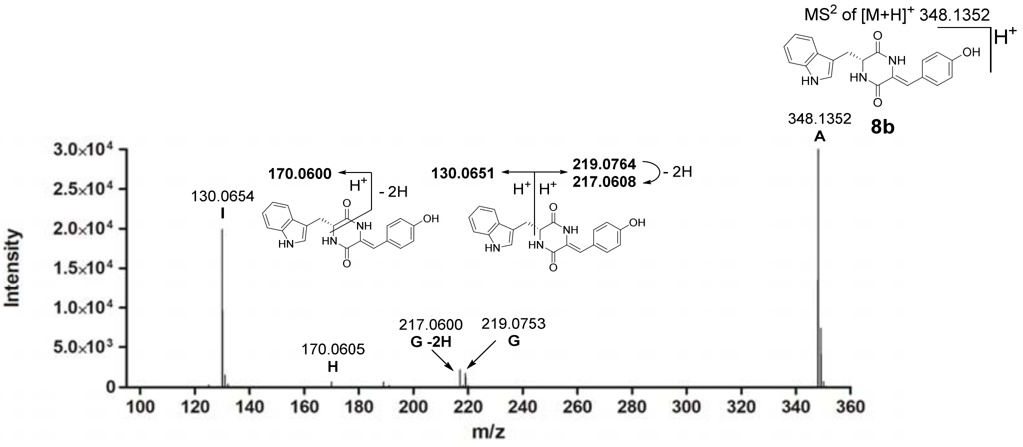

Figure S61: MS^2 spectrum of **8b**.

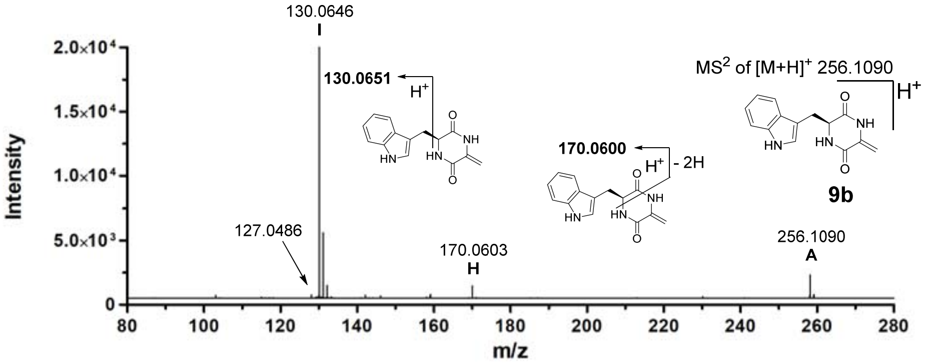

Figure S62: MS^2 spectrum of **9b**.

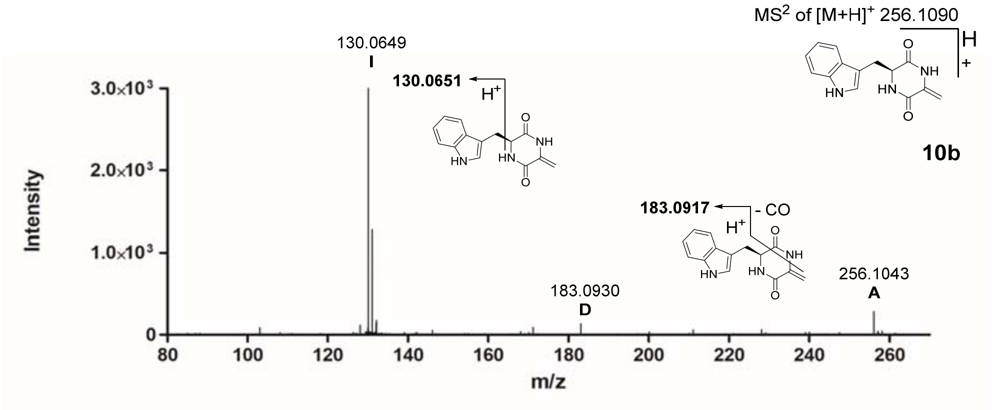

Figure S63: MS^2 spectrum of **10b**.

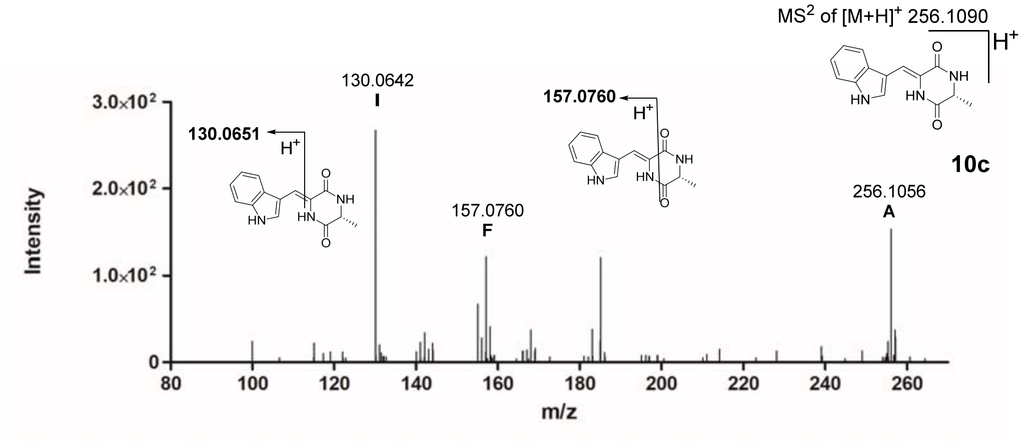

Figure S64: MS^2 spectrum of **10c**.

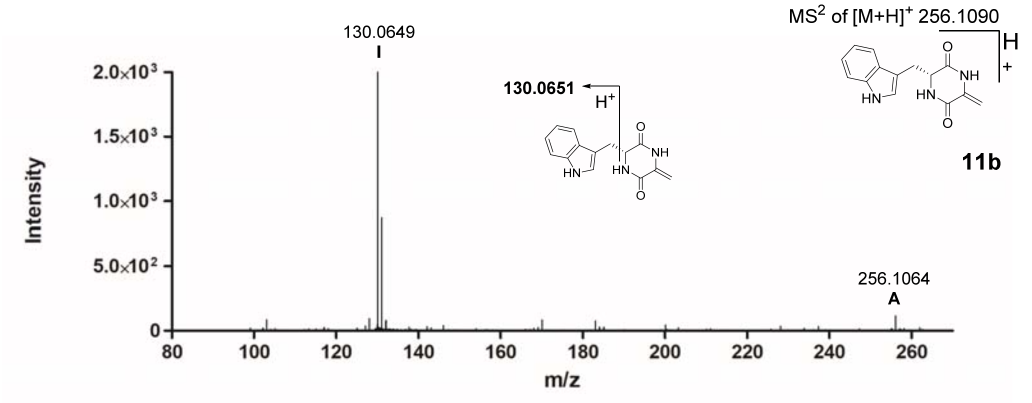

Figure S65: MS^2 spectrum of **11b**.

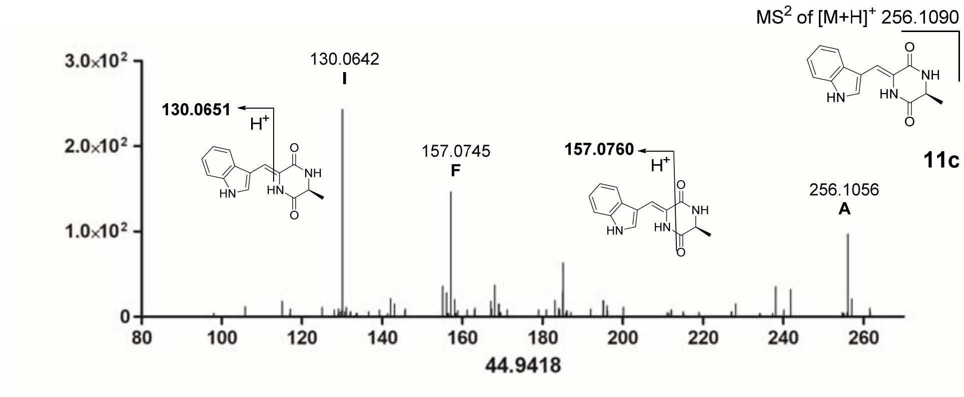

Figure S66: MS^2 spectrum of **11c**.

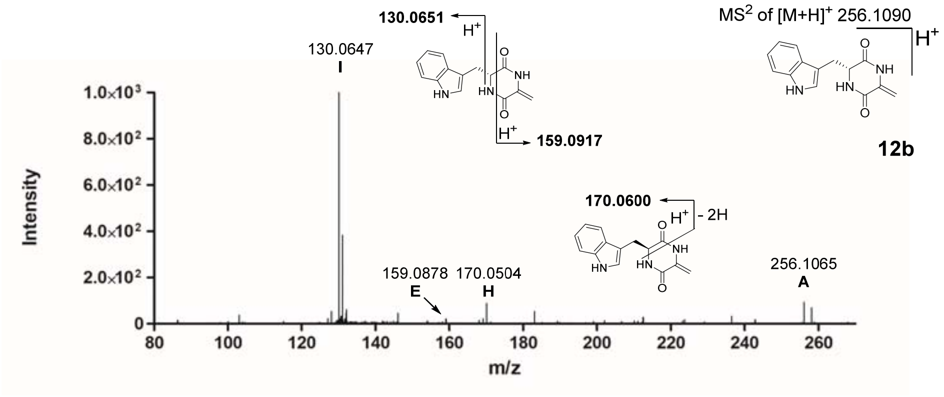

Figure S67: MS^2 spectrum of **12b**.

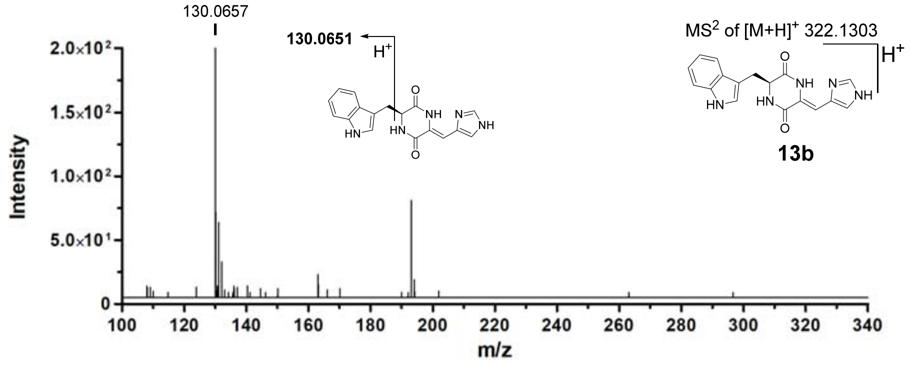

Figure S68: MS^2 spectrum of **13b**.

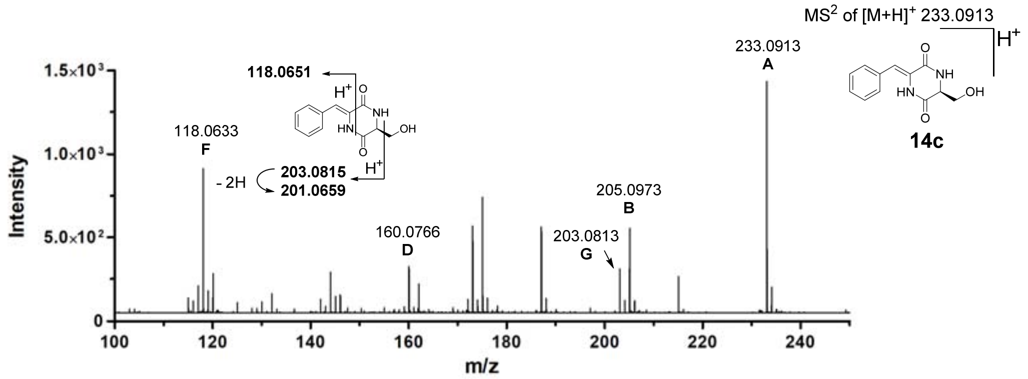

Figure S69: MS^2 spectrum of **14c**.

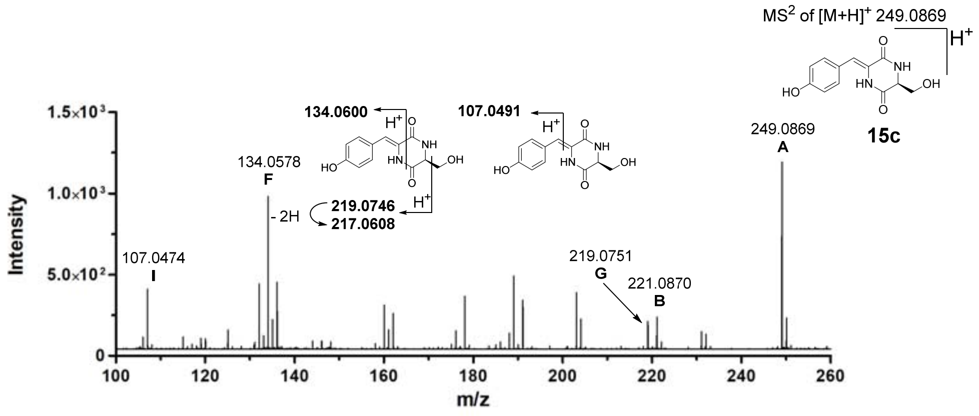

Figure S70: MS^2 spectrum of **15c**.

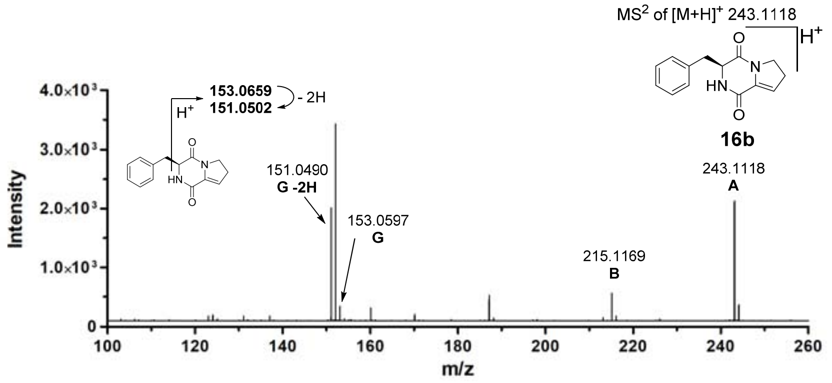

Figure S71: MS^2 spectrum of **16b**.

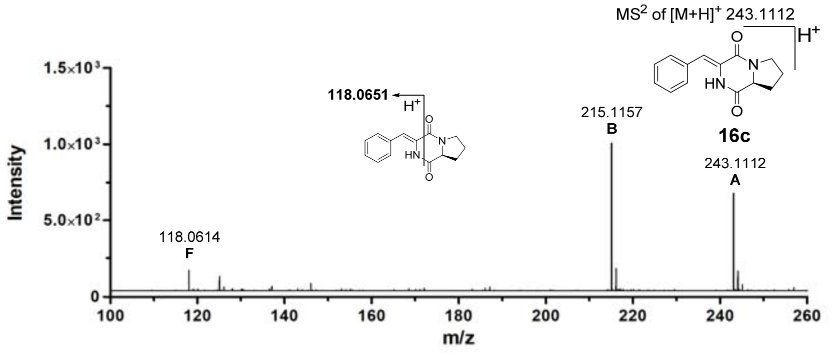

Figure S72: MS^2 spectrum of **16c**.

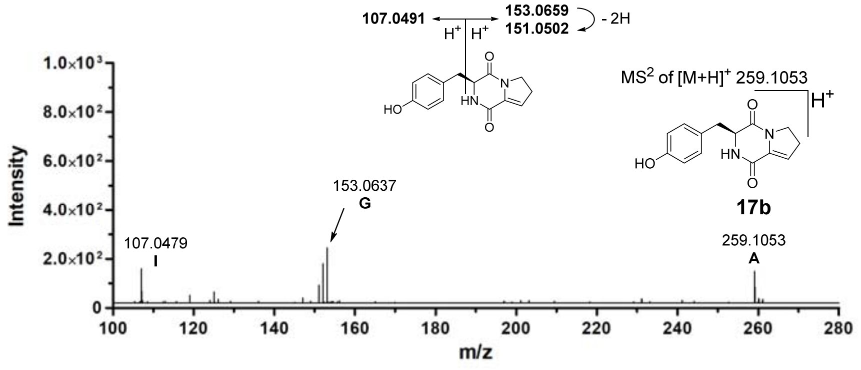

Figure S73: MS^2 spectrum of **17b**.

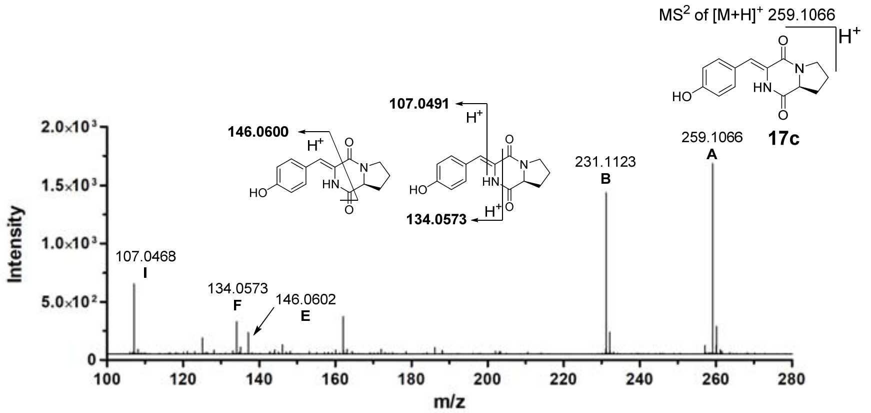

Figure S74: MS^2 spectrum of **17c**.

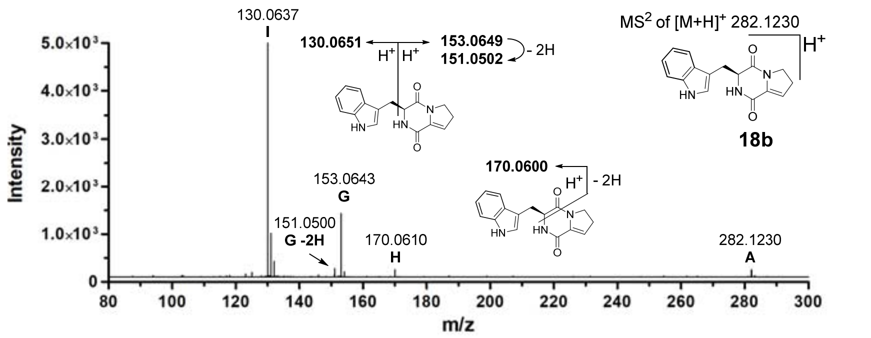

Figure S75: MS^2 spectrum of **18b**.

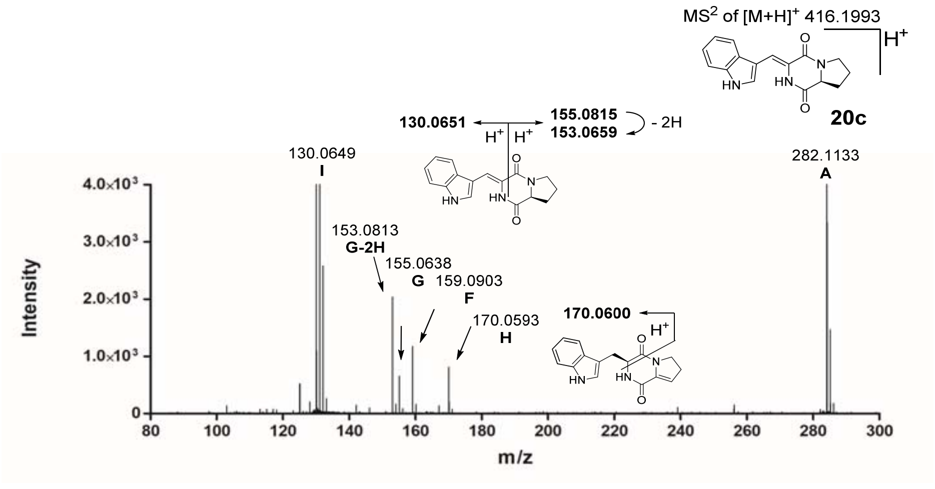

Figure S76: MS^2 spectrum of **20c**.

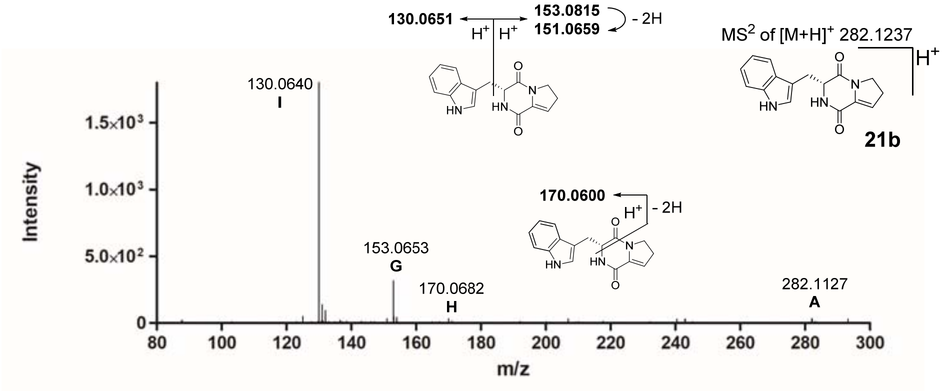

Figure S77: MS^2 spectrum of **21b**.

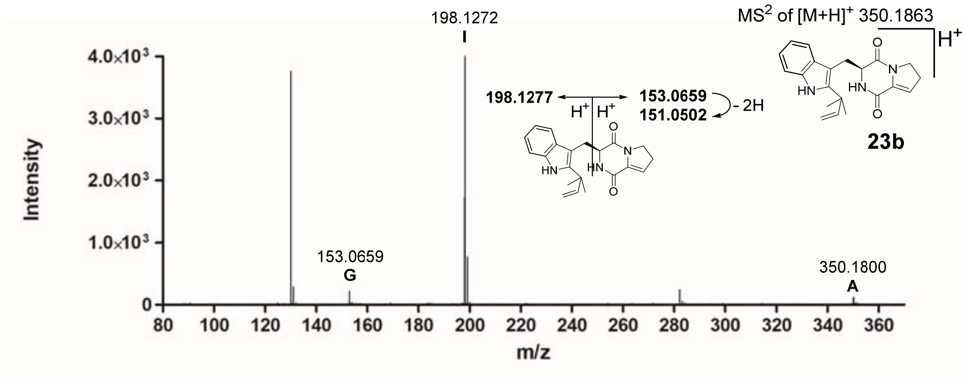

Figure S78: MS^2 spectrum of **23b**.

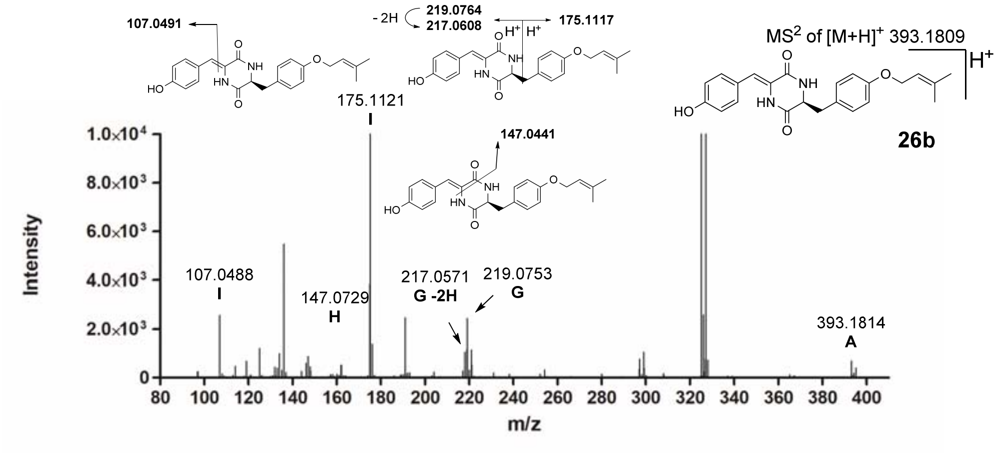

Figure S79: MS^2 spectrum of **26b**.

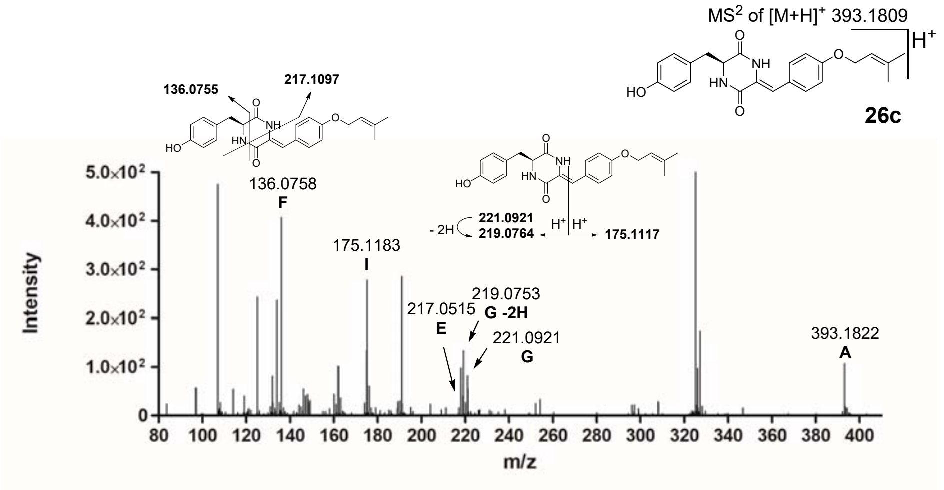

Figure S80: MS^2 spectrum of **26c**.

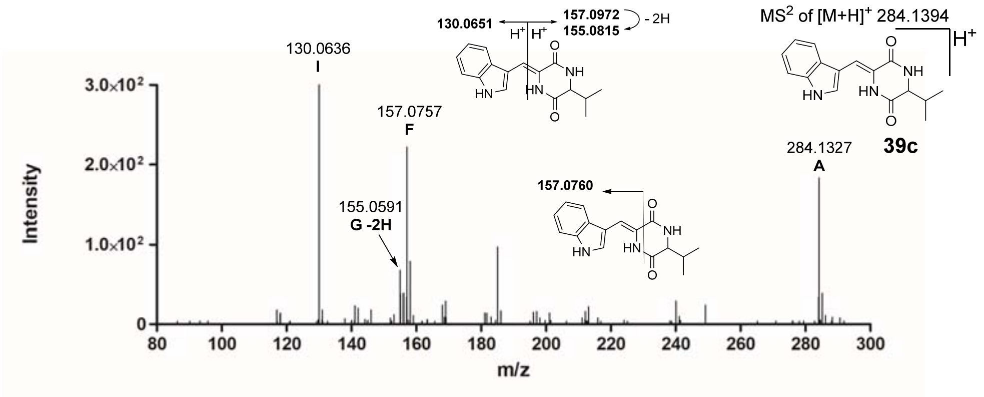

Figure S81: MS^2 spectrum of **39c**.

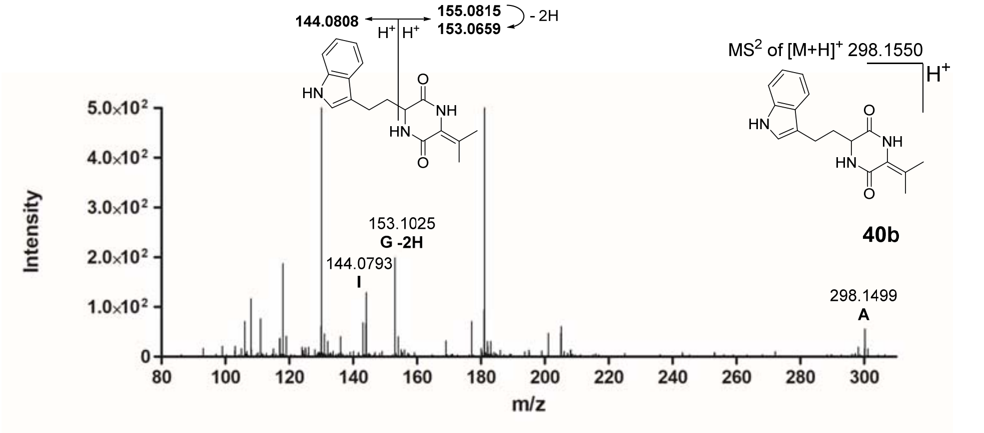

Figure S82: MS^2 spectrum of **40b**.

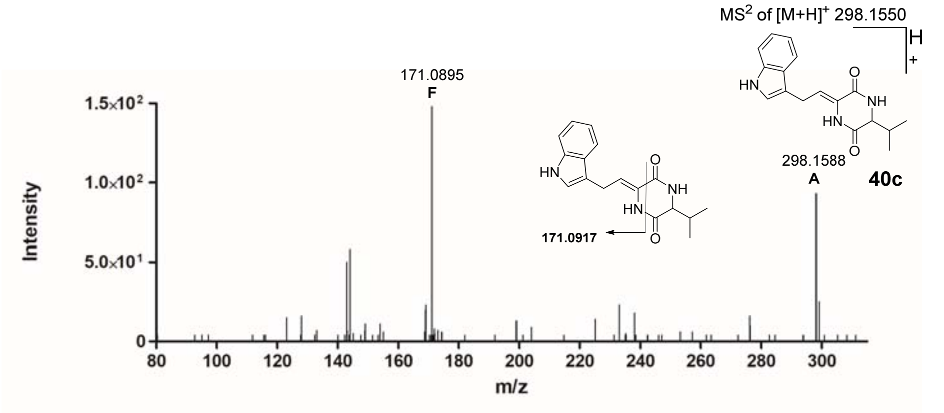

Figure S83: MS^2 spectrum of **40c**.

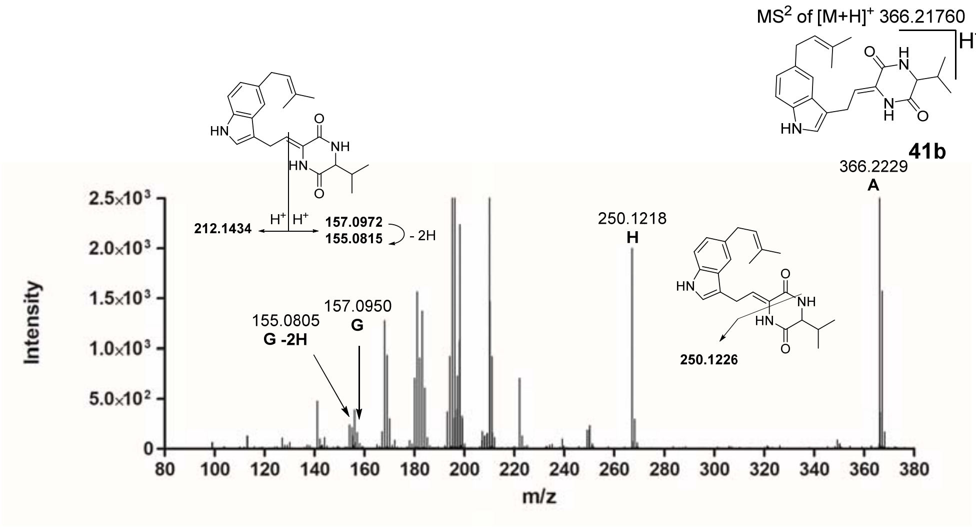

Figure S84: MS^2 spectrum of **41b**.

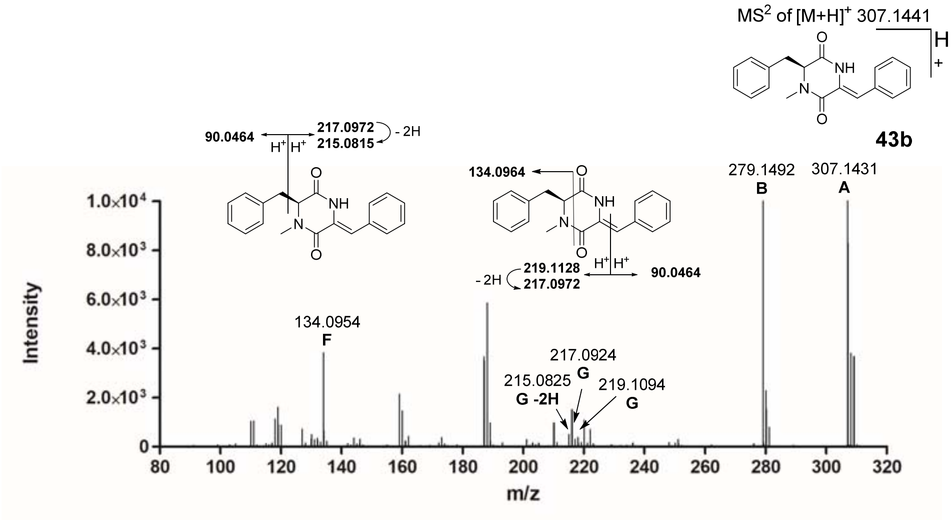

Figure S85: MS^2 spectrum of **43b**.

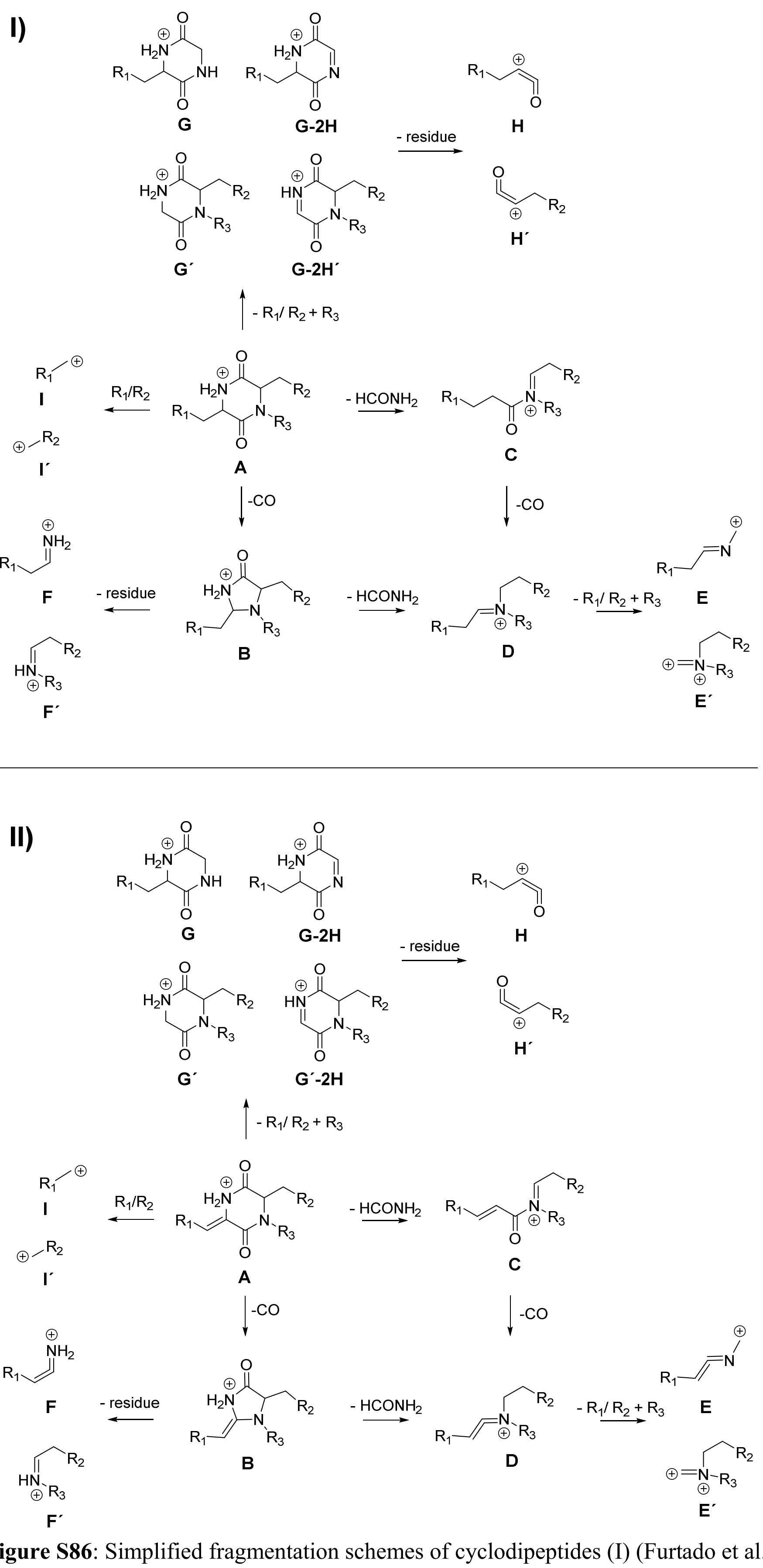

Figure S86: Simplified fragmentation schemes of cyclodipeptides (I) (Furtado et al. 2007; Guo et al. 2009) and didehydrogenated product (II). The product of the **b** series is illustrated as an example. Fragments **H´** and **I´** are not obtained from proline-containing CDPs.

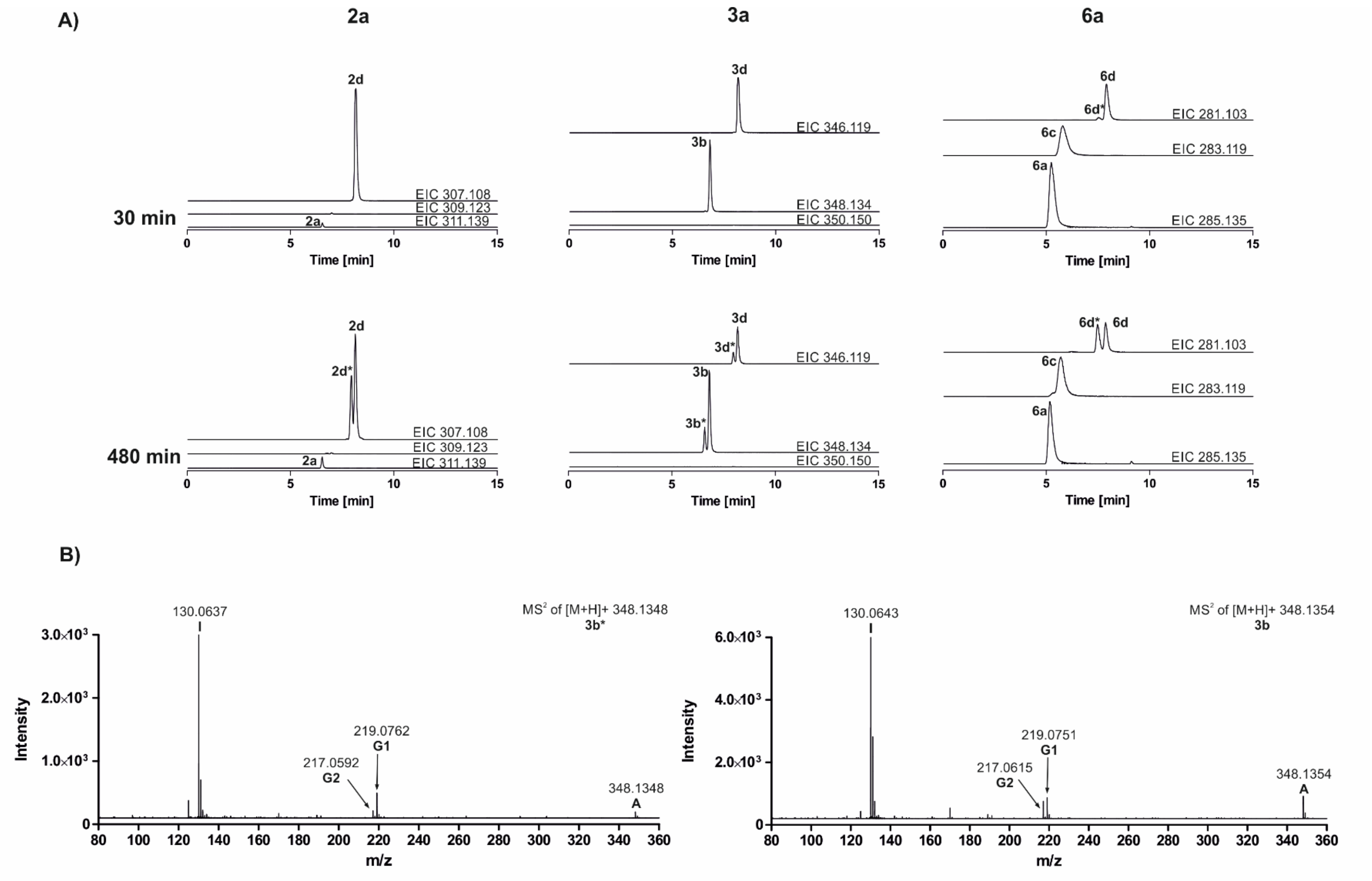

Figure S87: Extracted ion chromatograms of CDO-Np assays with a tolerance range of ± 0.005 after exposure to 22 °C and light for 30 min and 8 h (A). Assays were quenched and solved in methanol prior to the incubation at 22 °C and light. A second peak corresponding to the tetra-dehydrogenated product was formed after 8 h of incubation for all three cyclodipeptides. In the case of **3b**, a second peak corresponding to the di-dehydrogenated product was obtained as well after 8 h. Analysis of the fractionation pattern revealed that both compounds are *cyclo*-ΔTyr-L-Trp (B).

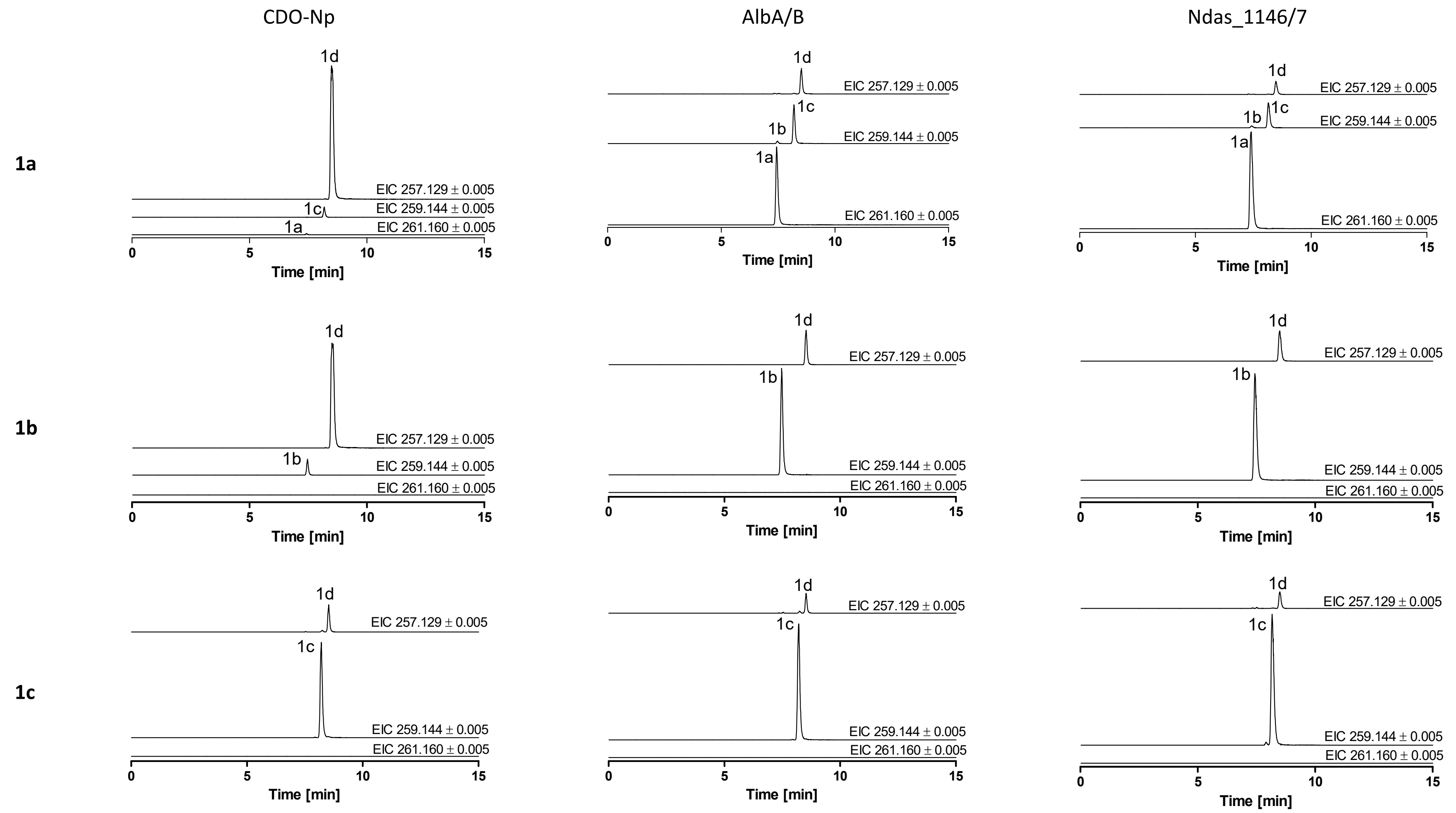

Figure S88: Incubation of the 3 CDOs with **1a**, **1b**, and **1c**. The reaction mixtures were incubated at 40 °C for 120 min.

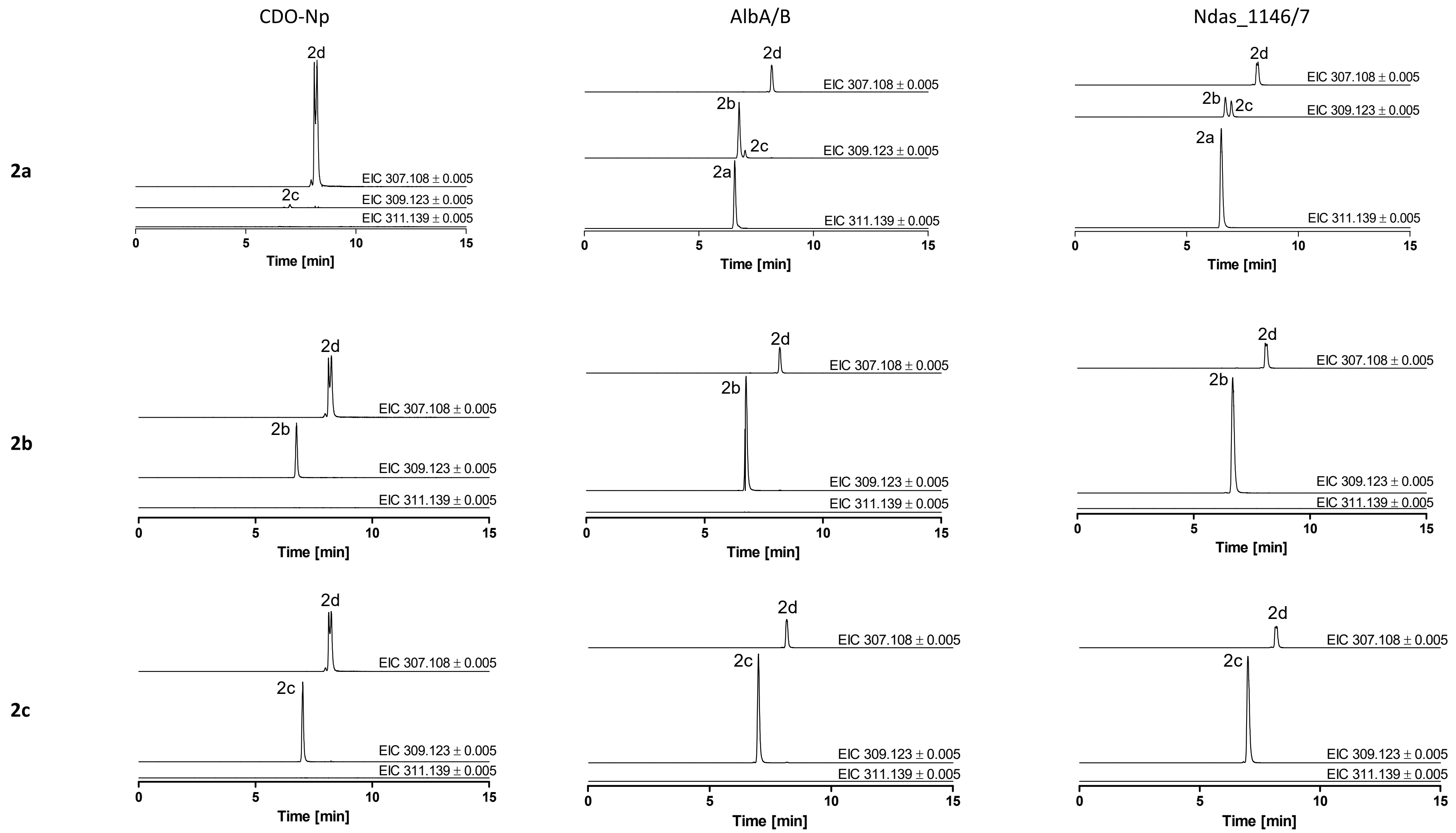

Figure S89: Incubation of the 3 CDOs with **2a**, **2b**, and **2c.** The reaction mixtures were incubated at 40 °C for 120 min.

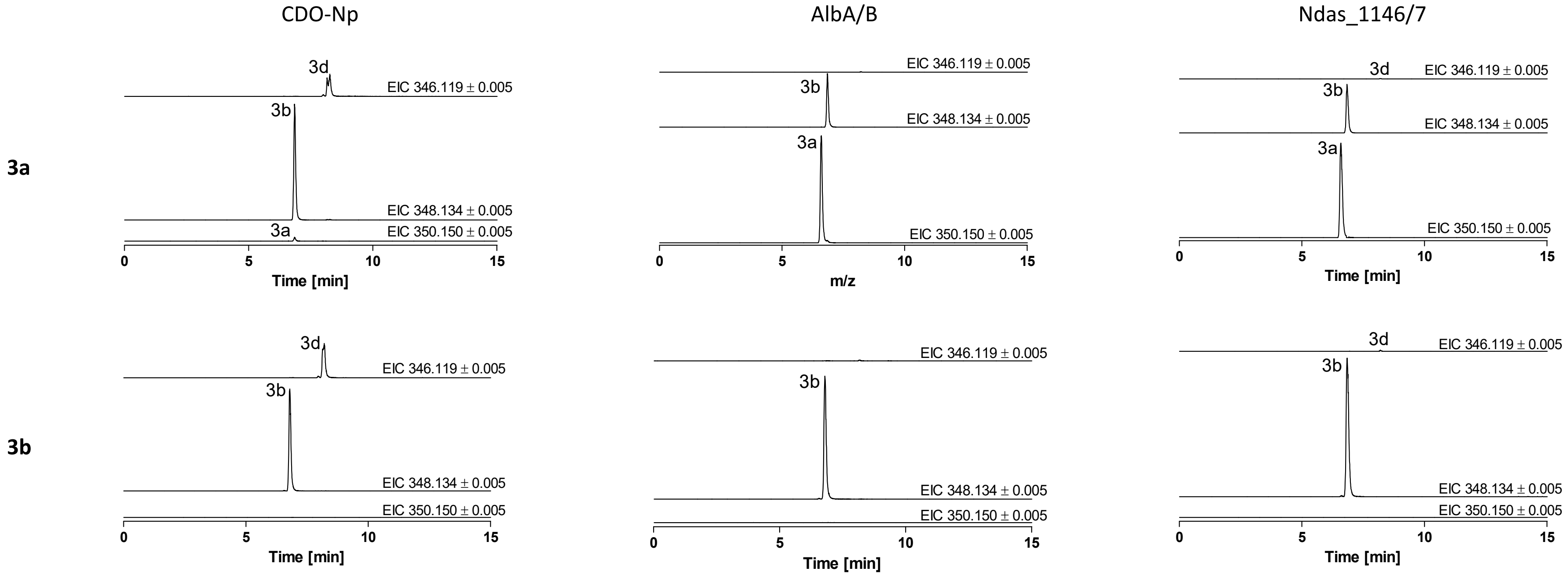

Figure S90: Incubation of the 3 CDOs with **3a** and **3b.** The reaction mixtures were incubated at 40 °C for 120 min.

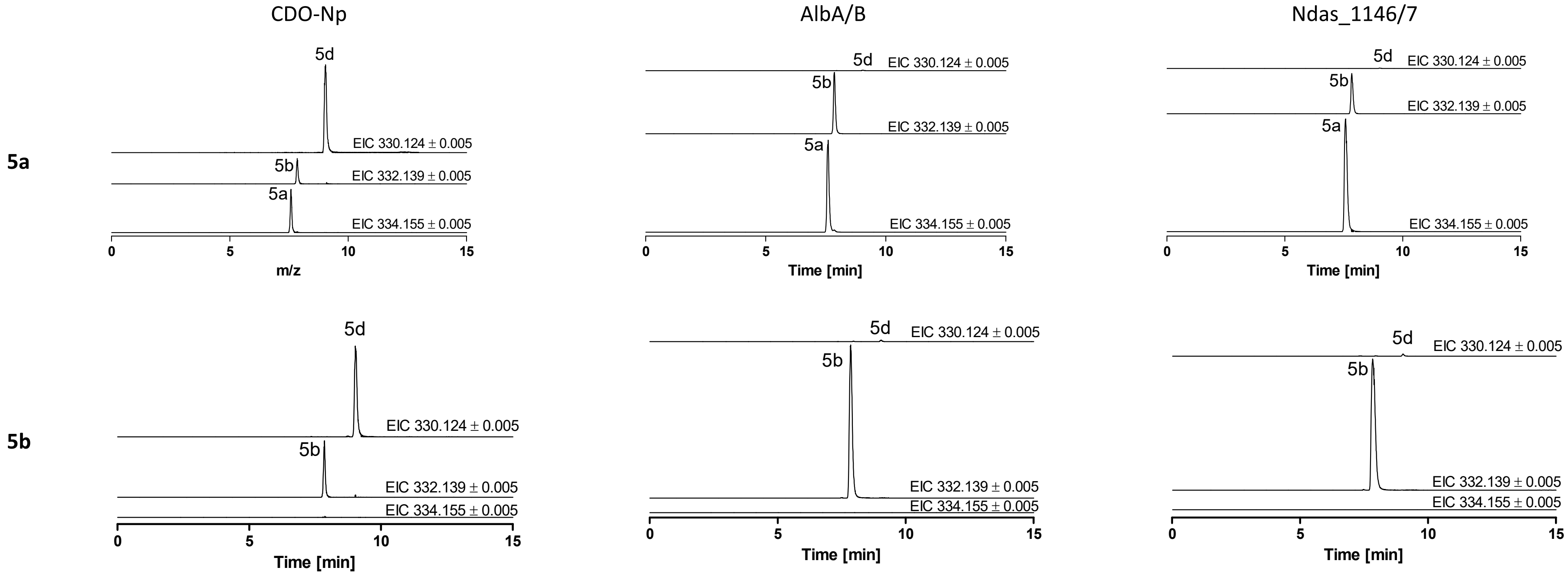

Figure S91: Incubation of the 3 CDOs with **5a** and **5b**. The reaction mixtures were incubated at 40 °C for 120 min.

References

Furtado NA, Vessecchi R, Tomaz JC, Galembeck SE, Bastos JK, Lopes NP, Crotti AE (2007) Fragmentation of diketopiperazines from *Aspergillus fumigatus* by electrospray ionization tandem mass spectrometry (ESI-MS/MS). J Mass Spectrom 42:1279-1286.

Guo Y-C, Cao S-X, Zong X-K, Liao X-C, Zhao Y-F (2009) ESI-MS^n study on the fragmentation of protonated cyclic-dipeptides. Spectroscopy 23:131-139.

Kanzaki H, Yanagisawa S, Nitoda T (2000) Biosynthetic intermediates of the tetradehydro cyclic dipeptide albonoursin produced by *Streptomyces albulus* KO-23. J Antibiot 53:1257-1264.
